# Critical evaluation of molecular tumour board outcomes following 2 years of clinical practice in a Comprehensive Cancer Centre

**DOI:** 10.1038/s41416-022-02120-x

**Published:** 2022-12-26

**Authors:** Alexander Scheiter, Frederik Hierl, Florian Lüke, Felix Keil, Daniel Heudobler, Sabine Einhell, Margit Klier-Richter, Nikola P. Konstandin, Florian Weber, Andrea Scheiter, Arne Kandulski, Sophie Schlosser, Lidia-Sabina Cosma, Hauke Tews, Andreas R. R. Weiss, Matthias Grube, Elisabeth Bumes, Peter Hau, Martin Proescholdt, Felix Steger, Anja Troeger, Sebastian Haferkamp, Lucas E. Reibenspies, Marco J. Schnabel, Christian Schulz, Konstantin Drexler, Maria E. Hatzipanagiotou, Stephan Seitz, Monika Klinkhammer-Schalke, Philipp Unberath, Diego F. Calvisi, Tobias Pukrop, Wolfgang Dietmaier, Matthias Evert, Kirsten Utpatel

**Affiliations:** 1 Institute of Pathology, University of Regensburg, 93053 Regensburg, Germany; 2 Bavarian Center for Cancer Research / BZKF, Regensburg, Bavaria Germany; 3 Department of Internal Medicine III, Hematology and Oncology, University Hospital Regensburg, 93053 Regensburg, Germany; 4 Fraunhofer-Institut für Toxikologie und Experimentelle Medizin ITEM-R, Abteilung für personalisierte Onkologie, 93053 Regensburg, Germany; 5 School of Engineering and Design, Chair of Ergonomics, Technical University of Munich, 85748 Garching, Germany; 6 Department of Internal Medicine I, University Hospital Regensburg, 93053 Regensburg, Germany; 7 Department of Surgery, University Hospital Regensburg, 93053 Regensburg, Germany; 8 Department of Neurology and Wilhelm Sander-NeuroOncology Unit, 93053 Regensburg University Hospital, 93053 Regensburg, Germany; 9 Department of Neurosurgery, University Hospital Regensburg, 93053 Regensburg, Germany; 10 Department of Radiotherapy, Regensburg University Medical Center, 93053 Regensburg, Germany; 11 Department of Pediatric Hematology, Oncology and Stem Cell Transplantation, University Hospital of Regensburg, 93053 Regensburg, Germany; 12 Department of Dermatology, University Hospital Regensburg, Regensburg, Germany; 13 Department of Urology, Caritas St. Josef Medical Center, University of Regensburg, 93053 Regensburg, Germany; 14 Department of Pneumology, University Hospital Regensburg, 93053 Regensburg, Germany; 15 University Medical Centre Regensburg, Department of Gynecology and Obstetrics, 93053 Regensburg, Germany; 16 Tumour Center—Institute for Quality Management and Health Services Research, University of Regensburg, 93053 Regensburg, Germany; 17 Friedrich-Alexander University Erlangen-Nuremberg, Chair of Medical Informatics, 91054 Erlangen, Germany

**Keywords:** Molecular medicine, Tumour biomarkers, Cancer genetics

## Abstract

**Background:**

Recently, molecular tumour boards (MTBs) have been integrated into the clinical routine. Since their benefit remains debated, we assessed MTB outcomes in the Comprehensive Cancer Center Ostbayern (CCCO) from 2019 to 2021.

**Methods and results:**

In total, 251 patients were included. Targeted sequencing was performed with PCR MSI-evaluation and immunohistochemistry for PD-L1, Her2, and mismatch repair enzymes. 125 treatment recommendations were given (49.8%). High-recommendation rates were achieved for intrahepatic cholangiocarcinoma (20/30, 66.7%) and gastric adenocarcinoma (10/16, 62.5%) as opposed to colorectal cancer (9/36, 25.0%) and pancreatic cancer (3/18, 16.7%). MTB therapies were administered in 47 (18.7%) patients, while 53 (21.1%) received alternative treatment regimens. Thus 37.6% of recommended MTB therapies were implemented (47/125 recommendations). The clinical benefit rate (complete + partial + mixed response + stable disease) was 50.0% for MTB and 63.8% for alternative treatments. PFS2/1 ratios were 34.6% and 16.1%, respectively. Significantly improved PFS could be achieved for m1A-tier-evidence-based MTB therapies (median 6.30 months) compared to alternative treatments (median 2.83 months; *P* = 0.0278).

**Conclusion:**

The CCCO MTB yielded a considerable recommendation rate, particularly in cholangiocarcinoma patients. The discrepancy between the low-recommendation rates in colorectal and pancreatic cancer suggests the necessity of a weighted prioritisation of entities. High-tier recommendations should be implemented predominantly.

## Background

In 2022, a total of 116 biomarker-dependent oncological therapeutic agents were listed by the Food and Drug Administration (FDA) [1], a result of remarkably dynamic development in recent years [2]. In parallel, next-generation sequencing techniques have entered the clinic, necessitating the implementation of multidisciplinary molecular tumour boards (MTB) to keep pace with these novel requirements.

While a few biomarkers have achieved tissue-agnostic approval by the FDA, such as tumour mutational burden (TMB) predictive for pembrolizumab [3] and neurotrophic tropomyosin-receptor kinase (NTRK) gene-fusions for entrectinib [4] and larotrectinib [5], most biomarker-drug combinations are tissue specific. Exemplarily, histologic context specificity has been demonstrated for alpelisib in phosphatidylinositol-4,5-biphosphate 3-kinase catalytic subunit alpha (PIK3CA)-mutated solid tumours [6], neratinib in HER2 and HER3 mutated [7], and vemurafenib in BRAFV600 altered cancers [8]. Furthermore, one should bear in mind that tissue-agnostic approvals remain controversial. Indeed, the seminal KEYNOTE-158 study, on which the FDA approved pembrolizumab in TMB-high tumours, excluded the common entity of colorectal cancer (CRC) by study design and did not recruit a single TMB-high cholangiocarcinoma [3]. Moreover, recent literature casts considerable doubt on the suitability of TMB as a predictive marker in CRC [9, 10].

These controversies exemplify the need for multidisciplinary case discussions in MTBs. Given that CRC is a frequent entity, this specific question has the potential to be addressed by future multicentric clinical trials. However, considering that a substantial proportion of cancers are rare cancers harbouring even more sporadic molecular alterations [11], structured evidence is hard to obtain. Therefore, new adaptive study designs [12] or multi-institutional collaborations through large consortia, such as the German DKTK MASTER programme [13], are mandatory. The latter programme impressed with the inclusion of 1310 patients, with 75.5% rare cancers, of whom 362 received molecularly informed therapies resulting in improved progression-free survival (PFS) compared to previous treatments (PFS2/PFS1 ratio >1.3 in 35.7% of evaluable patients) [13]. Similar clinical benefit in approximately one-third of patients has also been reported in a few additional larger-scale genomic medicine studies [12, 14]. At the same time, the objective response rates are generally low (11% in the MOSCATO-01 trial [14] and 0.9% in the ProfiLER trial [15]). Thus, despite a growing body of evidence, MTBs are often neither anchored in clinical routine (except for large cancer centres) nor represented in clinical guidelines. Major obstacles to generating more evidence include the following: late consideration of molecularly targeted therapies only at an advanced stage of disease, which can lead to non-implementation due to the patient’s death or deterioration of the general condition, and the difficulty in accessing recommended therapeutics [16]. In addition, health insurance companies play a critical role since they are responsible for the reimbursement of molecular diagnostics and the cost coverage of recommended off-label therapies.

Given the novelty of MTBs and the controversy surrounding their benefit in patients’ care, we established a longitudinal observational study at the Comprehensive Cancer Center Ostbayern (CCCO) with a large catchment area including rural regions. We present results from 2 years of expertise after MTB implementation to optimise the procedural, inclusion and outcome parameters.

## Methods

### Study design and patient population

The prospective observational study of the MTB of the CCCO was conducted following the Declaration of Helsinki and approved by the Ethics Committee of the University of Regensburg (protocol code 20-1682-101). This prospective registry study has been continuously recruiting since the end of 2019. All patients discussed in the MTB from 2019 to 2021 with available written consent were included in the current evaluation. For patient recruitment, the following clinical inclusion criteria have been defined: advanced disease, guidelines or standard therapy exhausted (patients should ideally just have begun the last-line therapy), sufficient life expectancy (6 months estimate), suitable tissue available, and open-mindedness to experimental treatments. In addition, rare oncological diseases lacking standard therapies were also accepted for MTB evaluation.

Additional procedural inclusion criteria for patient allocation to the MTB were: availability of the patient’s written consent; organ-specific tumour board decision stating the exhaustion of established therapeutic standards and recommending an MTB discussion; letter of referral and provision of comprehensive medical documentation (a most recent oncological record).

We held the MTB discussion weekly. Representatives from the affiliated departments and clinics were designated and actively involved in the case annotation and discussion based on a rotational schedule. The attendance of one trained specialist in oncology, one specialist in pathology, one molecular biologist with expertise in bioinformatics, and one rotational representative of the affiliated clinics and departments was the minimal requirement for the MTB meeting. In detail, the affiliated departments comprised the Department of Internal Medicine III (Hematology and Oncology), the Department of Surgery, the Department of Internal Medicine I (Gastroenterology), the Department of Neurology, the Department of Neurosurgery, the Department of Dermatology, the Department of Radiotherapy, the Department of Pediatric Hematology, Oncology and Stem Cell Transplantation, the Department of Urology, the Department of Gynecology, and the Department of Pneumology. The Institute of Human Genetics partly received selected case documents (with potential germline implications) before the discussions for additional screening without actively participating. Genetic counselling was suggested based on their assessment in addition to the criteria proposed by Mandelker et al. [17] for somatic sequencing and the input of two oncologists who had obtained a special qualification in genetic counselling. The stated aim of the MTB was to offer additional information to the treating physician to allow for genomically informed decisions. Thus, molecular and immunohistochemical analyses of the tumour were performed. The DKTK/NCT Master evidence grading was employed to stratify MTB recommendations (Supplementary Table S1) in conjunction with clinical parameters. An exception to the outlined workflow was the entity of lung cancer. Here, the thoracic cancers tumour board already discusses a broad array of molecular alterations as part of the clinical routine. Therefore, patients were referred to the MTB only in case of the detection of rare alterations without evidence-based therapeutic approaches. Molecular analysis was performed on the most relevant tissue reflecting the current state of disease progression (e.g., latest relapse or metastases). Exceptions to the defined inclusion criteria were occasionally made so that, in several cases, MTB diagnostics and discussion were conducted before the commencement of the last therapy line according to the guidelines. A decision from an organ-specific tumour board was necessary for this deviation from the protocol. Reasons for early inclusion were sparsity of tissue obtained from a biopsy (to avoid repeated cutting of the sample), an oncological assessment of a limited remaining life span, and an expected little benefit of the current or last-line therapy.

### Choice of analysis methods

The employed analysis methods comprised panel-based next-generation sequencing (NGS) for DNA and RNA, polymerase chain reaction (PCR)-based fragment-sizing for microsatellite instability (MSI), immunohistochemistry (IHC) for PD-L1, Her2, and mismatch repair (MMR) proteins MLH1, MSH2, MSH6 and PMS2. In addition, in the case of the detection of copy number variations, *MET* or *HER2* fluorescence in situ hybridisation (FISH) was performed as a confirmatory method. For most included patients, all methods mentioned above were used. The absolute numbers of employed methods are detailed in 'Results'.

### Documentation, follow-up and clinical reassessment

All MTB decisions were documented, including the most relevant literature citations on which the recommendations were primarily based. Structured follow-up data were collected from the attending physicians at prespecified time points 1, 3, 6, 12, 18 and 24 months after the MTB. The data collection period started on December 1, 2019 and was cut off on April 30, 2022. Structured follow-up comprised the following items: survival status, type of MTB-recommended therapy and timepoint of initiation, on/off-label therapy, reasons for non-implementation, other oncological therapies since the MTB discussion, response to MTB or alternative therapy and adverse effects. Clinical data were pseudonymized and documented primarily in Excel® version 16.0 (Microsoft Corporation, Redmond, WA, USA). For the final data assessment, clinical data were visualised in conjunction with genomic data (see below) in a local instance of cBioportal [18–21] provided by the MIRACUM consortium (https://github.com/buschlab/MIRACUM-cbioportal; last accessed August 4, 2022).

Responses were assigned to the categories progressive disease (PD), stable disease (SD), partial response (PR), mixed response (MR), and complete response (CR) based on radiological reports and clinical documentation for both MTB-recommended therapies and alternatively administered therapies (i.e., non-genomically informed therapies after an MTB discussion). MR was defined as a disease stabilisation or size reduction of a tumour lesion with concomitant progression at another location. This response class was included as it might indicate biological drug efficacy. Note that RECIST criteria were not employed for the retrospective evaluation of radiological responses. We determined the progression-free survival for the preceding therapeutic therapy line and the MTB-recommended or alternative therapies (time from MTB to progression or death). The PFS2/1-value indicates the ratio of the PFS of MTB or alternative treatment (PFS2) over the PFS of the preceding therapy (PFS1). PFS2/1 > 1.3 has been defined by previous precision oncology clinical trials to indicate intra-patient benefit and hence was adopted in the present study [13, 14]. In addition to the clinical follow-up, the patients’ overall survival (OS) relative to the date of the MTB was obtained from the cancer registry of the Regensburg Tumour Centre. Here clinical reports, death certificates issued by the local public health departments, and the registration offices of the respective residential districts are collected. Patients with no information on survival, patients lost to follow-up, or alive at the last medical visit were censored at the latest record date.

### Genomic, FISH, MSI, immunohistochemical and statistical analyses

The exact procedure is described in the Supplementary Methods section.

### Reporting summary

Further information on research design is available in the Nature Portfolio Reporting Summary linked to this article.

## Results

### Performed molecular analyses and patient population

This prospective registry study includes a total of 251 patients managed by the MTB of the CCCO over the indicated 2-year period.

For 262 case discussions of 251 patients, 147 biopsies (56.1%), 92 primary resection specimens (35.1%), 9 cytologies (3.4%), 6 pooled samples (2.3%) were analysed. In eight cases (3.1%), a denotation as either biopsy or resection is not applicable. In total, 149 analyses were performed on metastatic tumour samples (56.6%), 96 on primary tumour tissue (36.6%), 5 on pooled metastatic and primary tumour tissue (1.9%), and for 12 cases a distinction was not possible (4.6%, e.g., carcinoma of unknown primary [CUP]). The median age of analysed tumour specimens respective to the time of enrolment was 89 days (minimum age 0 days, maximum age 2931 days).

Overall, 237 out of 257 cases (92.2%) were evaluable for DNA and RNA alterations. Initially, in 22 cases, panel sequencing had failed and not reached sufficient coverage for analysis, 13 of which had been repeated, resulting in 2 additional evaluable cases.

In absolute numbers, the TSO500® panel was used in 214 (81.7%), the QIAseq Tumour Mutational Burden Panel® in 17 (6.5%), the Human Actionable Solid Tumour Panel Kit® in 12 (4.6%), a combination of the latter two panels in 3 (1.2%), the nNGMv2 custom panel in 5 (1.9%) and other DNA panels in 4 (1.5%) out of a total of 262 case discussions, while DNA panel sequencing was unavailable in 7 cases (2.7%).

For RNA panel sequencing, the TSO500® panel was used in 213 (81.3%), the FusionPlexLung-panel® in 37 (14.1%), a combination of the latter two panels in 1 (0.4%), and other RNA panels in 3 (1.2%) out of 262 case discussions.

Her2 IHC was available in 171 (65.5%) and confirmatory FISH analyses in 24 (9.2%) case discussions. MMR enzymes were assessed 173 times (66.0%) and MSI by PCR-based fragment-sizing 143 times (54.6%). PD-L1 scores (TPS, ICS and CPS) were determined in 197 cases (75.2%).

Across the entire cohort, 125 patients (49.8%) received treatment recommendations categorised into tiers according to the evidence of the given medication/ genetic alteration. Across all tiers, a total of 219 recommendations were given. 62 patients obtained more than one treatment recommendation (mean 1.74; maximum 4). Among all 251 patients, 47 (18.7%) received a genomically justifiable therapy. In contrast, 53 patients (21.1%) received alternative treatments, which comprised any systemic therapy administered without considering MTB recommendations (including in-label and off-label treatments) (Table 1). The remaining patients did not receive additional anticancer therapy. The alternative treatment cohort was a reference cohort to compare the patients receiving an MTB-recommended therapy. Gender and mean age of the patients and ECOG performance status at the MTB time revealed no differences between the two groups. The frequency of MTB recommendations was 35.9% for patients receiving alternative therapies. In the whole cohort, the highest evidence level of m1A was most frequent (24.8%), followed by m1C, (18.2%), m1B (16.5%), m2A (14.9%), and m2C (10.7%). m3 (5.8%), m2B (5.0%) and m4 (4.1%) were comparatively rare. In the subcohort analysis, m1A-evidence recommendations were more abundant in the MTB therapy cohort than in the alternative therapy cohort (36.2% versus 5.3%; *P* = 0.0133). Thus, recommendations with this high evidence level are implemented comparatively more often. In contrast, no significant difference could be demonstrated for recommendation levels below m1A.

**Table 1:** Patient characteristics.

	All patients	MTB therapy	Alternative therapy	*P* value
Population (*n*, %)	251 (100.00)	47 (18.73)	53 (21.12)	
Mean age, standard deviation (years)	59 ± 12.00	56.19 ± 14.04	55.87 ± 11.69	0.7608^a^
Sex				0.6854^b^
Male (*n*, %)	144 (57.37)	29 (61.7)	30 (56.7)	
Female (*n*, %)	107 (42.63)	18 (38.30)	23 (43.40)	
ECOG PS at MTB				
0	62	14	15	>0.9999^b^
1	55	13	13	>0.9999^b^
2	4	0	1	>0.9999^b^
5	8	0	0	
Recommendations (*n*, %)	125 (49.80)	47 (100.00)	19 (35.85)	
Evidence-level primary recommendation (*n*, %)				
m1A	30 (24.79)	17 (36.17)	1 (5.26)	0.0133^b^
m1B	20 (16.53)	8 (17.39)	4 (21.05)	0.735^b^
m1C	22 (18.18)	7 (15.22)	3 (15.79)	>0.9999^b^
m2A	18 (14.88)	6 (12.77)	5 (26.32)	0.2753^b^
m2B	6 (4.96)	1 (2.17)	1 (5.26)	0.5024^b^
m2C	13 (10.74)	3 (6.38)	2 (10.53)	0.6208^b^
m3	7 (5.79)	2 (4.35)	3 (15.79)	0.1444^b^
m4	5 (4.13)	3 (6.52)	0 (0.00)	0.5498^b^
Previous lines (median, range)	2, 0–10	2, 0–6	2, 1–9	0.9166^a^
Entity (*n*, %)				
CRC	36 (14.40)	1 (2.13)	6 (11.11)	0.1182^b^
iCCA	33 (13.20)	7 (14.89)	5 (9.26)	0.5395^b^
PDAC	18 (7.20)	0 (0.00)	3 (5.56)	0.2461^b^
Gastric Ca	16 (6.40)	5 (10.64)	3 (5.56)	0.4671^b^
Sarcoma	14 (5.60)	6 (12.77)	5 (9.26)	0.7507^b^
CUP	13 (5.20)	3 (6.38)	4 (7.41)	>0.9999^b^
pCCA	10 (4.00)	3 (6.38)	0 (0.00)	0.0973^b^
Breast Ca	10 (4.00)	0 (0.00)	3 (5.56)	0.2461^b^
eCCA	9 (3.60)	2 (4.26)	3 (5.56)	>0.9999^b^
Gallbladder Ca	8 (3.20)	2 (4.26)	0 (0.00)	0.2141^b^
NSCLC	8 (3.20)	2 (4.26)	1 (1.85)	0.5964^b^
Salivary gland Ca	8 (3.20)	3 (6.38)	2 (3.70)	0.6615^b^
Urothelial Ca	7 (2.80)	1 (2.13)	4 (7.41)	0.3685^b^
HNSCC	6 (2.40)	0 (0.00)	3 (5.56)	0.2461^b^
HGSC	5 (2.00)	0 (0.00)	2 (3.70)	0.4974^b^
Prostatic Ca	5 (2.00)	0 (0.00)	2 (3.70)	0.4974^b^
Lymphoma	4 (1.60)	2 (4.26)	1 (1.85)	0.5964^b^
Melanoma	4 (1.60)	1 (2.13)	0 (0.00)	0.4653^b^
HCC	4 (1.60)	0 (0.00)	1 (1.85)	>0.9999^b^
NEC	4 (1.60)	1 (2.13)	0 (0.00)	0.4653^b^
Oesophageal Ca	4 (1.60)	0 (0.00)	2 (3.70)	0.4974^b^
Others (*n* ≤ 3 in all patients)	24 (9.60)	8 (17.02)	4 (7.41)	0.2169^b^

The *P* values were calculated as follows (comparison MTB and Alternative therapy columns): ^a^Mann–Whitney *U*, ^b^Fisher’s exact test.

The median line of preceding therapies was identical between groups (*n* = 2).

The overall distribution of cancer types was similar between the MTB and alternative therapy group, without significant differences among entities. However, on descriptive evaluation, CRC, pancreatic ductal adenocarcinoma, and breast cancer appeared more frequently in the alternative therapy group.

### Qualitative and quantitative analysis of MTB recommendations

**Fig. 1:**
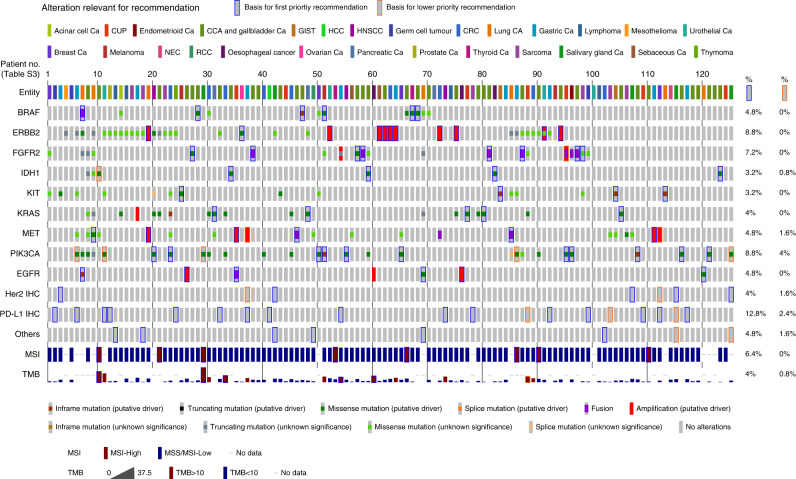
Alterations as a foundation for MTB recommendations. Oncoprint representation of alterations bearing relevance for MTB recommendations in ≥3.2% of patients (see Supplementary Fig. S1 for all alterations). Blue frames highlight individual alterations on which MTB recommendations were based for first-priority recommendations and orange frames for lower-priority recommendations. The percentage of first- and lower-priority recommendations is given on the right. The order of patients can be translated to PatID using Supplementary Table S3. The row “others” subsumes individual cases with BCL2 loss by IHC, androgen receptor expression, and FGF CNV. MSI microsatellite stability status, TMB tumour mutational burden in mutations per megabase, CUP carcinoma of unknown primary, Ca cancer, CCA cholangiocarcinoma, GIST gastrointestinal stromal tumour, HCC hepatocellular carcinoma, HNSCC head and neck squamous cell cancer, CRC colorectal cancer, NEC neuroendocrine carcinoma, RCC renal cell cancer.

We assessed the frequency of a given biomarker that had been used for molecular evidence-level- assigned treatment recommendations (Fig. 1). The highest percentage of tier 1 recommendations was based on PD-L1 immunohistochemistry (12.8% of patients with tier 1/2.4% lower tier recommendation), i.e., immune-oncological therapies with PD-L1/PD-1 inhibitors. This was followed by recommendations based on the biomarkers *PIK3CA* (8.8% of patients with tier 1/4% lower tier recommendations), molecularly confirmed *ERBB2* alterations (8.8%/0%), and *FGFR2* (7.2%/0%). Note that one patient with a novel FGFR2-NDC80 fusion, who received a therapy recommendation for FGFR inhibitors, has been described previously as a case study [22]. MSI (also comprising two patients with only dMMR, in whom MSI PCR could not be conducted due to the unavailability of normal control tissue) was the next most frequently underlying biomarker for treatment recommendations (6.4%/0%). *MET* (4.8%/1.6%), *EGFR* (4.8%/0%), *BRAF* (4.8%/0%), *KRAS* (4%/0%), *IDH1* (3.2%/0.8%), and *KIT* (3.2%/0%) were used for recommendations in ≥4 patients each. Importantly, also Her2 IHC was relevant for recommendations (without confirmed concomitant molecular alterations) (4%/1.6%), and a high TMB (defined as >10 mutations/MB) was occasionally used as a basis for MTB recommendations (4%/0.8%). Finally, many more recommendations in individual patients were based on biomarkers that occurred with low frequency (Supplementary Fig. S1). A detailed list of individual recommendations (in the order of patients in Fig. 1) can be found in Supplementary Table S3. The complete information on clinically reported underlying mutations is depicted in Supplementary Table S4, on reported CNVs in Supplementary Table S5, and on detected genetic rearrangements in Supplementary Table S6.

Corresponding to these biomarkers, the most commonly recommended drug classes were: immune checkpoint inhibitors (38/219 [17.4%]), HER2 inhibitors (31/219 [14.2%]), combination therapies (21/219 [9.6%]), PARP inhibitors (20/219 [9.1%]), FGFR inhibitors (17/219 [7.8%]), PI3K inhibitors (13/219 [5.9%]), multi tyrosine kinase inhibitors (13/219 [5.9%]), MEK inhibitors (8/219 [3.7%]), MET inhibitors (8/219 [3.7%]), ALK inhibitors (6/219 [2.7%]), EGFR inhibitors (6/219 [2.7%]), IDH1 inhibitors (5/219 [2.3%]), mTOR inhibitors (5/219 [2.3%]), CDK4/6 inhibitors (4/219 [1.8%]), androgen blockade (3/219 [1.4%]) and others (21/219 [9.6%]).

**Fig. 2:**
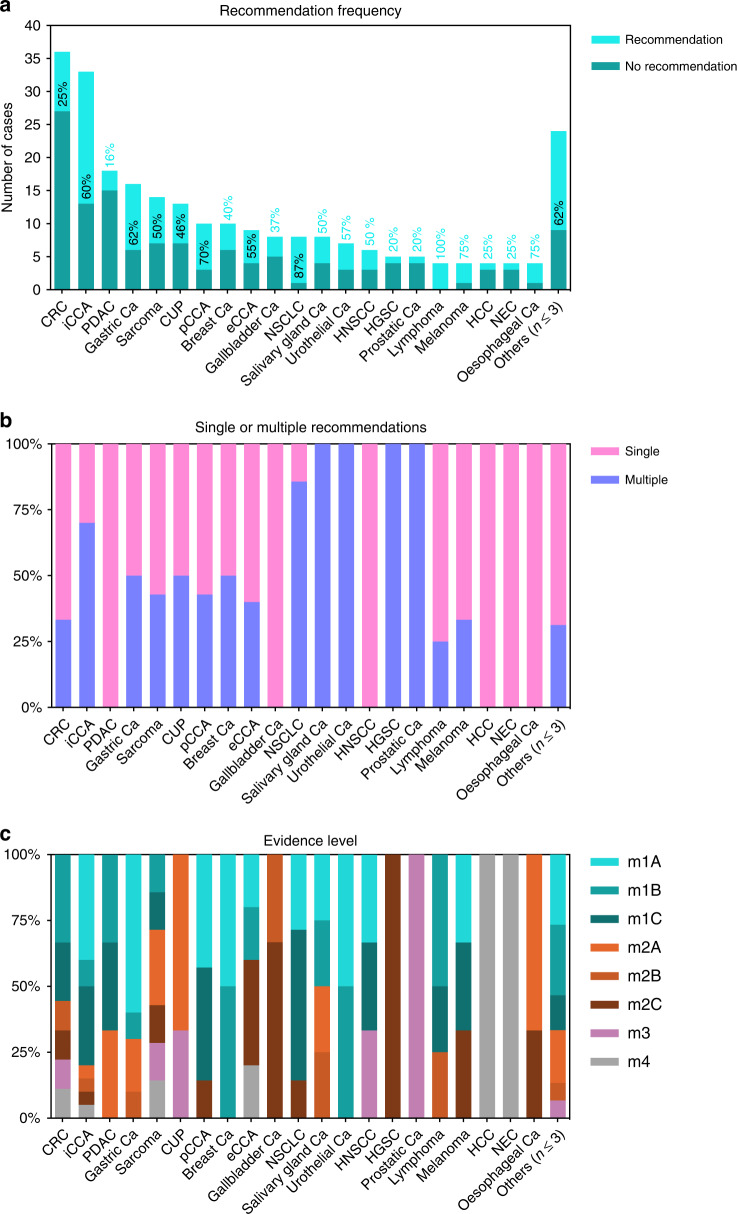
Comparison of MTB recommendations by the entity. **a** Recommendation frequency by cancer type. The percentage of patients receiving treatment recommendations by the MTB is indicated. **b** Percentage of patients receiving either single or multiple MTB recommendations. **c** Evidence level of primary treatment recommendation by entity as NCT/DKTK grade. CRC colorectal cancer, iCCA intrahepatic cholangiocarcinoma, PDAC pancreatic ductal adenocarcinoma, Ca cancer, CUP carcinoma of unknown primary, pCCA perihilar cholangiocarcinoma, NSCLC non-small cell lung cancer, HNSCC head and neck squamous cell cancer, HGSC high-grade serous carcinoma, HCC hepatocellular carcinoma, NEC neuroendocrine carcinoma.

We compared the recommendation rates between entities to address whether certain cancer types benefit more from MTB discussions (Fig. 2a). Focusing on the ten most abundant entities in our cohort, we can demonstrate that colorectal cancer (CRC) and pancreatic ductal adenocarcinoma (PDAC) yielded only low-recommendation rates of 25% and 16%, respectively. Intermediate-recommendation frequencies were observed for sarcoma (50%), carcinoma of unknown primary (CUP) (46%), breast cancer (40%), and gallbladder carcinoma (37%). In contrast, comparatively high-recommendation rates were achieved for intrahepatic cholangiocarcinoma (iCCA; [60%]), perihilar cholangiocarcinoma (pCCA, [70%]), extrahepatic cholangiocarcinoma (eCCA; [55%]), and gastric cancer (62%). Concerning the less frequently included entities, the high-recommendation frequency for NSCLC should be interpreted cautiously, since only selected cases were discussed in our MTB. It is noteworthy that a high frequency of therapy recommendations (62%) was yielded for rarely included entities summarised as “others” with *n* ≤ 3. Concordantly, the entities with higher recommendations frequencies were also more likely to obtain multiple recommendations per case discussion (Fig. 2b), and the distribution of evidence levels (Fig. 2c) favoured higher evidence levels in the entities with high- and intermediate-recommendation frequencies (except for CUP, where m1 evidence is not possible per definition).

### Clinical outcome of MTB-guided versus alternative therapies

In total, 47 patients (18.73%) received therapies recommended by the MTB, while 53 (21.12%) received alternative treatments following the MTB discussion. An assessment by a board-certified oncologist demonstrated that MTB-guided treatments were off-label in 96.6%. (according to the European Medicines Agency [EMA] approval status) at the time of the MTB decision. In contrast, only 39.6% of the alternatively administered therapies were off-label at the latter timepoint (see Table 2 for details on MTB therapies and Supplementary Table S7 for alternative therapies). The analysis of drug classes yields the following distribution for MTB-based treatments: immune checkpoint inhibitors were predominantly employed (14/47 [29.8%]), followed by combination therapies (6/47 [12.8%]), Her2 inhibitors (4/47 [8.5%]), FGFR inhibitors and inhibitors of multiple tyrosine kinases (3/47 [6.4%] each), ALK inhibitors, androgen blockade, CDK4/6 inhibitors, EGFR inhibitors and IDH1 inhibitors (2/47 [4.3%] each) as well as a single case of administration of cytostatic chemotherapy, a JAK inhibitor, a MEK inhibitor, a nucleoside analogue, a PARP inhibitor, a PI3K and a RET inhibitor (1/47 [2.1%] each). Alternatively, administered therapies were very heterogenous, including immune checkpoint inhibitors and classical chemotherapy (see Supplementary Table S7 for details).

**Table 2:** Overview of patients receiving MTB-recommended therapies.

PatID	Cancer type	Board recommendation	Rationale	EL	L (time of MTB/ EMA)	R	Side effects	Sequential MTB therapy	Comment
5	T-PLL	Ruxolitinib	JAK2 gain of function mutation, STAT5B mutation	m3	off	PR			Potent reduction of leucocytosis, however, death due to persistent neutropenia and mycotic pneumonia
19	Lung adenocarcinoma	Mobocertinib	EGFR exon 20 insertion	m1A	off	PR	Mucositis, increase of renal retention parameters		
22	iCCA	Pemigatinib	FGFR2-BICC fusion	m1A	off	PR			
28	CUP (adenocarcinoma, pelvic and hepatic)	Binimetinib + trametinib + cetuximab	BRAF V600E gain	m2A	off	PR			
29	Gastric adenocarcinoma, diffuse type	Pembrolizumab	MMRd and PD-L1 (CPS 15)	m1A	off	PR			
31	Malignant peritoneal mesothelioma	Crizotinib	STRN-ALK	m1C	off	PR		Brigatinib	Sequential therapy based on the detection of p.L1196M ALK resistance mutation, mixed response [30]
43	Adenocarcinoma of the esophagogastric junction	Trastuzumab-deruxtecan	Her2 expression (DAKO score 3 +)	m1A	off	PR			Following progression on CF-trastuzumab
42	Mucosal melanoma	Imatinib	KIT gain	m1A	off	MR		Nilotinib	PD under sequential therapy
1	Lung adenocarcinoma	Afatinib + paclitaxel	ERBB2 gain	m1C	off	SD	Haematological + gastrointestinal toxicity	Trastuzumab-Deruxtecan	
7	iCCA	Ivosidenib	IDH1 gain	m1A	off	SD	Acute renal failure		Disease stabilisation on CT shortly after termination of treatment
9	Uterine leiomyosarcoma	Pembrolizumab	POLE loss, PD-L1 (CPS 8)	m2C	off	SD			
10	Salivary duct carcinoma	Bicalutamide + trenantone	AR expression (IRS 12)	m1A	off	SD			
16	iCCA	Olaparib	BRCA2 loss	m1C	off	SD	Pancytopenia		Dose reduction required
21	Gastric adenocarcinoma	Trastuzumab-deruxtecan	ERBB2-amplification (IHC2 +/FISH +), simultaneous MET amplification	m1A	off	SD			MET amplification as a putative mechanism of resistance to previous trastuzumab therapy
26	iCCA	Ivosidenib	IDH1 gain	m1A	off	SD			
33	pCCA	Pembrolizumab + lenvatinib	MSI-High, MMRd, PD-L1 (CPS 4)	m1A	off	SD	Eczema		
36	GIST	Regorafenib	KIT gain	m1A	on	SD	Gastrointestinal toxicity, hand-foot syndrome	Imatinib	
38	pCCA	Pembrolizumab	MSI-High, MMRd, PD-L1 (CPS 8)	m1A	off	SD			
40	Type B3 thymoma	Pembrolizumab	PD-L1 (CPS 91)	m1B	off	SD			
2	Oesophageal adenocarcinoma	Pembrolizumab	PD-L1 (CPS 20)	m1A	off	PD	Loss of appetite		Clinical deterioration
4	Papillary thyroid carcinoma	Pembrolizumab	MSI-high, MMRd, MSH2 mutation, TMB 7.8 mut/Mb, and PD-L1 (TPS 15)	m1A	off	PD			
6	Dedifferentiated liposarcoma	Palbociclib	CDK4-CNG, MDM2-CNG, CDK4-CNOT2 fusion of uncertain significance	m1B	off	PD			
8	dCCA	Dabrafenib + trametinib	BRAF gain	m2C	off	PD			
11	Leiomyosarcoma	Selpercatinib	RET gain	m2A	off	PD	Acute on chronic renal failure		
12	Dedifferentiated chondrosarcoma	Pembrolizumab	PD-L1 (CPS 25)	m1C	off	PD			
14	Salivary adenocarcinoma, NOS	Pembrolizumab	MSI-High + TMB (37.5 mut/Mb)	m2A	off	PD			
15	Nonseminomatous Germ cell tumour	Pembrolizumab	PD-L1 (CPS 45)	m1B	off	PD			
17	iCCA	Pembrolizumab	POLD1	m1B	off	PD	Haematological toxicity, dyspnoea, fatigue		
18	Gallbladder adenocarcinoma	Pembrolizumab + lenvatinib	POLE	m2C	off	PD	Exacerbation of arterial hypertension		
20	Endometrioid carcinoma	Alpelisib + fulvestrant	PIK3CA gain	m2A	off	PD	Hyperglycaemia		
23	Lung adenocarcinoma	Afatinib	EGFR kinase domain duplication (exon 18–25)	m1C	on	PD	Gastrointestinal toxicity		
24	eCCA	Copanlisib + cisplatin + gemcitabine	PIK3CA gain	m4	off	PD			Scheme modified from NCT01460537 study. Autopsy: 60% regression in hepatic tumour manifestation. Death due to septic pneumonia.
30	iCCA	Pemigatinib	FGFR2-MYH12 fusion	m1A	off	PD			
32	Breast neuroendocrine carcinoma	Palbociclib + fulvestrant	SMARCA4 Mutation	m4	off	PD	Neutropenia		
34	Adeno-CUP	Palbociclib	SMARCA4 mutation	m3	off	PD			
35	Salivary adenocarcinoma, NOS	Bicalutamide + trenantone	AR expression (IRS 6)	m1C	off	PD		Pembrolizumab	Based on PD-L1 (CPS 30, m1B), PFS > 63d, PR
37	Primary mediastinal germ cell tumour	Pembrolizumab	PD-L1 (CPS 72)	m1B	off	PD			
39	Extragonadal germ cell tumour	Alectinib	DHX57 (exon1)-ALK (exon2) fusion	m2A	off	PD	Acute on chronic renal failure		
3	SFT	Pazopanib	FGF1,2,10 CNG	m4	on	NA			Change of diagnosis, NAB2-STAT6 fusion detection
13	pCCA	Binimetinib + capecitabin	NRAS	m1C	off	NA	Gastrointestinal toxicity		
25	Peripheral T-cell lymphoma	Azacitidine + ascorbate	TET2 mutation	m1B	off	NA			
27	iCCA	Pemigatinib	FGFR2-PHGDH Fusion	m1A	off	NA	Ocular toxicity		
41	Adenocarcinoma of the esophagogastric junction	VESTIGE Study, NCT03443856 (control arm)	PD-L1 (CPS 17)	NA	off	NA			
44	Gallbladder adenocarcinoma	Pembrolizumab	PD-L1 (CPS 41)	m1A	off	NA			
45	CUP (undifferentiated carcinoma)	Axitinib + pembrolizumab	PD-L1 (CPS100)	m2A	off	NA			
46	Colorectal cancer	Trastuzumab-deruxtecan	*ERBB2-* amplification (CNG 19.581-fold change/IHC3 + / FISH + )	m1B	off	NA			
47	Urothelial carcinoma	Pertuzumab + trastuzumab + taxol	*ERBB2-* amplification (CNG 2.342-fold change/IHC2 + / FISH + )	m1B	off	NA			

*EL* evidence level (ZPM scheme), *R* response, *L* label, *NA* not applicable, *iCCA* intrahepatic cholangiocarcinoma, *pCCA* perihilar cholangiocarcinoma, *dCCA* distal cholangiocarcinoma, *T-PLL* T-cell prolymphocytic leukaemia, *GIST* gastrointestinal stromal tumour, *SFT* solitary fibrous tumour, *CUP* carcinoma of unknown primary, *SD* stable disease, *PD* progressive disease, *PR* partial remission, *MR* mixed response, *MSI* microsatellite instability, *CNG* copy number gain, *IHC* immunohistochemistry, *FISH* fluorescence in situ hybridisation, *gain* gain of function mutation, *loss* loss of function mutation.

**Fig. 3:**
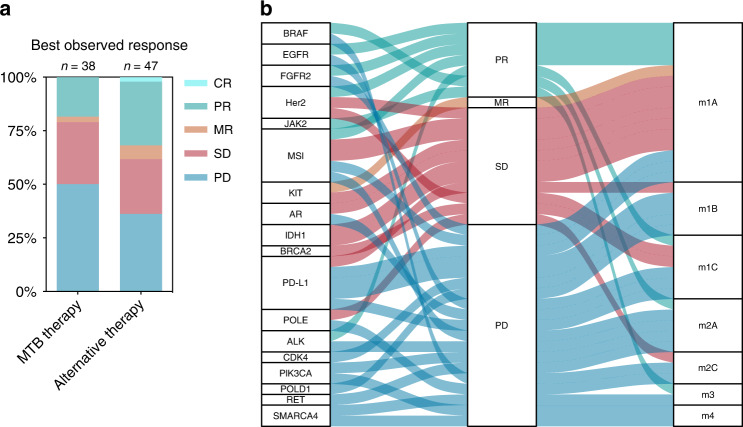
Overview of best-observed responses. **a** Stacked bar chart of best-observed responses to MTB-recommended therapies as opposed to alternative therapies. **b** Alluvial plot displaying the best-observed responses in relation to alteration type and NCT/DKTK evidence level.

A total of 38 MTB-based treatments and 47 alternative therapies were clinically evaluated for response (Fig. 3a). The clinical benefit rate (defined here as complete response [CR] + partial response [PR]+ mixed response [MR]+ stable disease [SD]) was 50.0% for MTB and 63.8% for alternative treatments. In detail, 7 PR (29.8%), 1 MR (2.6%), 11 SD (29.0%), and 19 PD (50.0%) were observed for MTB-based therapies, while 1 CR (2.1%), 14 PR (29.8%), 3 MR (6.4%), 12 SD (25.5%) and 17 PD (36.2%) were described for alternative therapies. Next, the distribution of responses concerning the biomarkers underlying the recommendations was analysed (Fig. 3b). PR were obtained for treatments based on *BRAF*, *EGFR*, Her2, *JAK2*, *ALK* and MSI. The MR was established on an activating KIT mutation, and SD were yielded by therapies founded on Her2, MSI, *KIT*, AR, *IDH1*, *BRCA2*, PD-L1 and *POLE*. Treatments established on *CDK4*, *PIK3CA*, *POLD1*, and *RET* alterations led to PD. Interestingly, among the patients experiencing clinical benefit from MTB therapies, 63.2% had evidence levels of m1A (12/19), 15.8% had m1C evidence (3/19), and 5.3% had m1B, m2A, m2C and m3 evidence (1/19 each). Altogether, 84.2% of patients (16/19) had m1 evidence from the same entity. This distribution differs among patients who did not experience clinical benefit from MTB-recommended therapies, where only 52.6% of patients (10/19) had m1 evidence, while 47.4% (9/19) had evidence level of m2 or below (Fig. 3b).

**Fig. 4:**
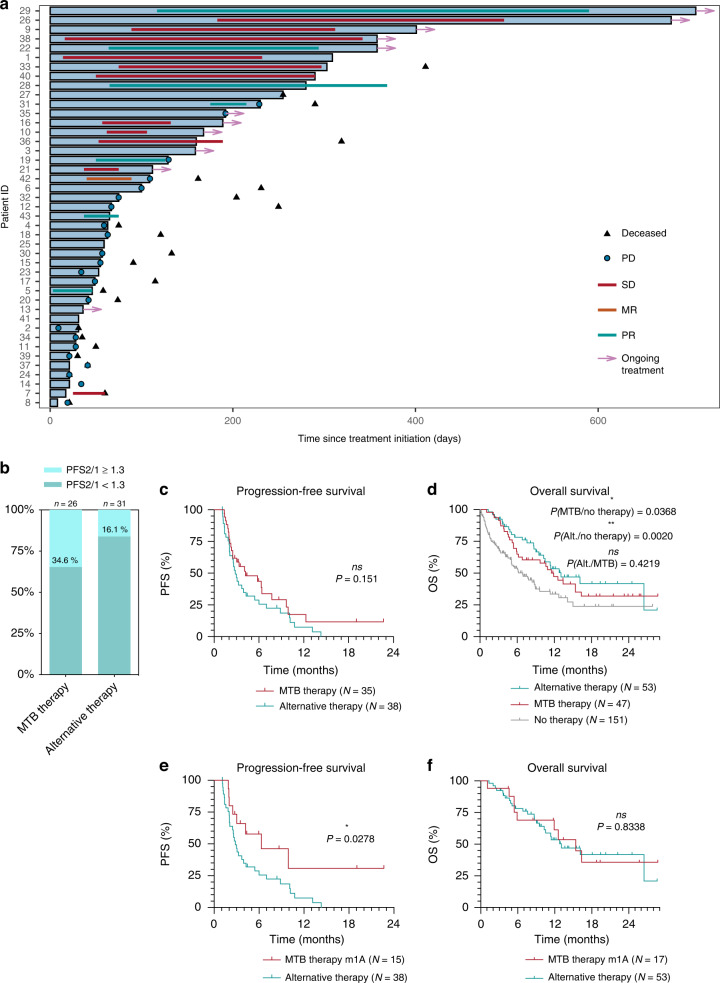
Survival outcome measures. **a** Swimmer plot of 43 patients receiving an MTB-recommended therapy with available follow-up data. Blue bars indicate the duration of MTB-recommended therapy. **b** Comparison of the frequency of PFS2/1 ratios ≥1.3 and <1.3 between MTB-recommended and alternative therapies in evaluable patients. Progression-free survival (**c**) and overall survival (**d**) over the entire cohort comparing patients receiving MTB-recommended therapies, alternative therapies after the MTB, and patients without further therapies. Progression-free survival (**e**) and overall survival (**f**) achieved by tier m1A-based MTB therapies as opposed to all alternative non-MTB-recommended therapies. The *P* value was calculated by the log-rank test.

To further analyse the efficacy of MTB-recommended therapies, we looked at treatment durations (Fig. 4a). In total, 11 patients received MTB-recommended treatments for more than 200 days. Among these, 10 had documented responses (3 PR and 6 SD), indicating that a certain proportion of MTB patients can benefit significantly from the recommended therapies. The recommendations for these patients were based on MSI and *FGFR2*-fusions (two cases each), *IDH1*, *POLE*, *ERBB2*, *BRAF* mutations, and PD-L1 expression. Also, ten treatments were still ongoing at the data cut-off.

Due to the heterogeneity of the cohort, PFS2/1 ratios were studied for intra-patient comparisons. 26 patients were evaluable for PFS2/1 in the MTB cohort and 31 in the alternative treatment cohort. PFS2/1 ratios over 1.3 were 34.6% and 16.1%, respectively (Fig. 4b; Supplementary Tables S8 and S9 for details).

Altogether, a median PFS of 4.20 months was obtained for MTB therapies as opposed to 2.83 months for alternative treatments, which did not reach statistical significance (95% CI of ratio 0.8762 to 2.508; *P* = 0.1509; Fig. 4c). An encouraging difference in OS for the MTB therapy (median 11.90 months) compared to no treatment (median 6.90 months; 95% CI of ratio 1.1114 to 2.671; *P* = 0.0368; Fig. 4d) was detectable. A significant difference could be observed in the OS of alternative therapy (median 13.07 months) versus no treatment (median 6.90 months; 95% CI of ratio 11.200 to 2.987; *P* = 0.0020; Fig. 4d), while MTB therapy and alternative therapy were not significantly different in terms of OS (*P* = .4219; Fig. 4d). Given that clinical benefit was rather achieved in the case of higher evidence levels, we also compared m1A-based MTB therapies to alternative therapies. For this selected group, PFS was significantly longer for m1A-MTB therapies (median 6.30 months) than alternative therapies (median 2.83 months; 95% CI of ratio 1.027 to 4.814; *P* = 0.0278; Fig. 4e). Despite the difference in PFS, OS was not different between these two groups (Fig. 4f).

OS and PFS were also compared in the CCA cohort, since a comparatively high number of MTB therapies had been administered in this entity, and a particular emphasis was placed on CCA by the ESMO guideline NGS recommendation [23] (Supplementary Fig. S2). While these comparisons were not statistically significant (median OS MTB_CCA_ 12.57 months; median OS alternative_CCA_ 11.33 months, median OS no therapy_CCA_ 8.43 months), a trend was detected for PFS in favour of the MTB therapy in CCA (median PFS MTB_CCA_ 9.90 months, median PFS alternative_CCA_ 2.70 months; 95% CI of ratio 1.06 to 12.67; *P* = 0.1170). In this analysis, a CCA harbouring an IDH1 mutation with long-term survival of >22 months is particularly interesting (PatID 26). However, due to the low sample size and the heterogeneity of cohorts, propensity score matching between cohorts was not possible for all survival analyses. Thus, the results should be interpreted cautiously, since confounders cannot be controlled.

## Discussion

In the presented MTB observational study, 49.8% of the 251 included patients received treatment recommendations based on IHC and NGS results. This number per se indicates that the methods employed are sufficient to detect relevant alterations for the intention of off-label treatment in a cohort of patients with advanced cancers. In 18.7% of patients, the treatment recommendation was implemented. Different factors influence this figure: due to the observational cut-off, several patients remained under last-line therapy and did not yet continue with the MTB-recommended therapy, treating physicians opted against the treatment administration, health insurers denied therapy cost coverage, or the patients deceased soon after the MTB. When comparing treatment recommendation and implementation frequencies with other genomically informed trials, similar results were obtained: the MTB in Baltimore reached 24% and 16% [24], in Vienna 54% and 23% [25], in Cleveland 49% and 11% [26], and 54% and 28% in Freiburg [16]. Of note, the DKTK/MASTER trial yielded much higher recommendation rates of 88%, while the implementation frequency was 31.8% [13]. A lower threshold could partly explain these high-recommendation rates. In the latter study, 45.3% of recommendations were based only on preclinical data (m3) and theoretical reasoning (m4), contrasting to 9.9% in our study. Moreover, in the DKTK/MASTER trial, more complex diagnostic methods were employed, such as whole genome/ exome and RNA sequencing, which could account for a higher detection rate of alterations. Nonetheless, the authors purport that our study’s targeted DNA and RNA sequencing methods were well suited to uncover off-label treatment options for about half of the recruited patients while being comparatively affordable methods. Of note, the addition of IHC for Her2, MMR proteins and PD-L1 accounted for 17.60% of primary recommendations, and we thus consider them an integral part of our MTB diagnostics. Moreover, MSI PCR yielded another 4.8% of primary recommendations, and in conjunction with MMR proteins, MSI was detected in 6.4% of patients. The authors advise against relying exclusively on panel-based MSI analysis. Not only did the NGS-based MSI analysis commonly fail (unevaluable in 78 out of 214 TSO panels [36.5%]), but also two cases of panel MSI were false negative, and one was false positive compared to PCR MSI. Beyond MSI/MMR, the addition of PD-L1 remains disputable. While 12.8% of primary recommendations were based on PD-L1 scoring (TPS, ICS and CPS), only one of five patients (PatID 40; a thymoma with a CPS of 91) reached SD on single agent immune checkpoint blockade, while the other four were progressive. While in some centres, PD-L1, MMR proteins, and Her2 immunohistochemistry are considered routine diagnostic, the authors believe these analyses an essential part of MTBs given the dynamic development of clinical studies and the fact that the resulting therapies are often off-label and should therefore be closely monitored in a study setting.

In addition to these insights on the chosen methods, we also noted different recommendation frequencies depending on the cancer type. This finding could help develop a weighted prioritisation system based on the entity in the future to optimise precision oncology workflows. This is highlighted by the relatively low-recommendation frequencies for CRC, breast cancer and PDAC, whose incidences are high. Other relatively frequent tumours like biliary tract and gastric cancer have high-recommendation rates and are thus more suited for comprehensive molecular analysis. Our real-world data align with observations made in the DKTK cohort [13] and MOSCATO-01 trial [14], emphasising the utility of molecularly informed therapies in selected, mostly rare, tumour entities. The 2020 ESMO guidelines also favour NGS in CCA, which is clearly mirrored by our cohort, while the use for gastric cancer is discouraged [23]. Regarding gastric cancer, our cohort yielded high-priority target recommendations for targeted therapy, such as MSI, Her2 expression, and PD-L1, while also uncovering rare but promising alterations such as ROS1 fusion and MET-amplifications. Although some of these alterations are already part of the standard therapy for gastric cancer, we consider a holistic reassessment of these parameters throughout the disease as reasonable and would assign a high priority to this entity. NGS for CRC and PDAC should only be implemented for research purposes according to the ESMO guidelines [23], a conclusion supported by this study. Furthermore, the ESMO guidelines recommend the routine use of NGS for lung cancer, CUP and prostate cancer. Our cohort offers limited insight into these entities due to low case numbers. However, we found it fruitful to refer selected cases that showed non-standard genetic aberrations from our thoracic tumour board to the MTB. The MTB offers an additional discussion platform for potentially actionable alterations in lung cancer patients and, due to its nature, can dedicate more time to the individual patient. This allows for comprehensive therapy recommendations such as concomitant *HER2*/*MET*-amplifications as a potential primary resistance mechanism and *MET* and *BRAF* fusions that cannot be as easily assessed in the thoracic tumour board. In addition, our data show that recommendations with high evidence levels were implemented more often and had better chances of attaining clinical responses. Thus, lower-priority evidence recommendations should be regarded with caution, because success rates are low and often clinically challenging to realise. Clinical benefit could more often be obtained from high evidence levels in the same entity (m1) and not from evidence derived from other entities or preclinical studies (m2 or below). On top of that, PFS was significantly longer for tier m1A-based MTB therapies than for alternative non-MTB therapies.

Regarding outcome measures, our study reports a clinical benefit rate (CBR; CR + PR + MR + SD) of 50.0% for MTB-recommended and 63.8% for alternatively administered treatments. In contrast, the PFS2/1 ratios were 34.6% and 16.1% for MTB and alternative treatments, thus favouring the MTB cohort. This discrepancy between CBR and PFS ratios might partly be explained by the fact that MTB-recommended therapies were off-label in 96.6%, while alternative therapies were off-label only in 39.6%. This distribution implies that in several cases, available in-label treatments were still available, so the MTB was not always initiated after the last-line treatment had begun. This contradicts the defined inclusion criteria and reflects that strict criteria cannot always be sufficiently met in clinical reality. Possible reasons include fast progression and considerable processing times for molecular diagnostics from enrolment until the MTB discussion (median 28 days).

The current observational study achieved an objective response rate (ORR) of 2.8% over the entire cohort (7 PR among 251 patients analysed) or 14.9% considering only those patients who were administered an MTB therapy (7/47). Again, this figure could potentially improve after a longer observation time since several patients were not yet evaluable for response at the data cut-off. While we acknowledge that an ORR of 14.9% is low, one should consider that this has been achieved in a heavily pretreated cohort. Moreover, disease control is sometimes just as beneficial for these incurably ill patients. This assumption is highlighted by a patient with CCA (PatID 26) who achieved SD under ivosidenib therapy for 22 months.

In the meantime, EMA has approved several MTB-recommended therapies in the respective genomic contexts, such as pembrolizumab for microsatellite instable [27] or pemigatinib for FGFR2 fusion-positive CCA [28]. With the benefit of hindsight, this justifies several MTB recommendations. Moreover, these cases clearly indicate that MTBs can facilitate allocating promising new unapproved therapeutic options to patients.

In our study, we observed significantly improved OS for MTB therapies compared to no treatment, which was also the case for alternative therapies, while PFS slightly favoured MTB therapies. We consider this additional encouraging evidence to use the MTB as a valuable clinical instrument for treatment allocation beyond the last-line therapy. At the same time, these results should be interpreted with caution due to the heterogeneity of cohorts. Finally, our study demonstrates the feasibility of establishing an MTB with a diffuse rural catchment area [29], providing its benefits also to spatially dispersed patients in Eastern Bavaria.

## Supplementary information

Supplementary methods

Supplementary Figures 1 and 2 captions

Supplementary tables

Figure S1

Figure S2

Reporting summary checklist

STROBE Checklist

## Data Availability

The datasets generated during and/or analysed during the study are not publicly available due to restrictions of the research ethics protocol but are available (in anonymized form) from the corresponding author upon reasonable request.
