# Supplementary material for: Critical evaluation of molecular tumour board outcomes following 2 years of clinical practice in a Comprehensive Cancer Centre
Source: Br J Cancer. 2022 Dec 26;128(6):1134–47. doi: 10.1038/s41416-022-02120-x (PMC10006213; doi:10.1038/s41416-022-02120-x)
Supplement: Supplementary file 1 — Supplementary methods [file 41416_2022_2120_MOESM1_ESM.docx]

**Supplementary Methods**

**2.4.1 Genomic analyses**

The employed standard panel transitioned from the 486-gene containing QIAseq Tumour Mutational Burden Panel® (QIAGEN N.V., Venlo, Netherlands) to the 523-gene containing TruSight Oncology 500® panel (TSO500®; llumina Inc., San Diego, CA, USA) over the study period. Initially, RNA was analysed separately using the FusionPlexLung-panel® (ArcherDx Inc., Boulder, CO, USA), while later, the RNA analysis was included in the TSO500 panel. Occasionally, when only limited sample DNA could be retrieved, the Human Actionable Solid Tumour Panel Kit (QIAGEN N.V.) was utilized. In addition, lung cancer samples have often been analysed outside the MTB context, so results from the nNGMv2 custom panel (Nationales Netzwerk Genomische Medizin, Germany) or the Human Actionable Solid Tumour Panel Kits (QIAGEN N.V.) were available.

In detail, H&E stained slides were assessed by a board-certified pathologist, and the position of the tumorous and surrounding tissue (if present) was marked. The percentage of tumour cells was visually estimated. For DNA and RNA extraction, paraffin sections were prepared and deparaffinized through a gradient of xylol and ethanol. Using a 0.01% methylene blue counterstain, the marked areas were selectively scratched off using a scalpel. The extraction was performed automatically on a Maxwell® RSC instrument (Promega Corporation, Madison, WI, USA) according to the manufacturer’s instructions. The resulting DNA and RNA concentrations were evaluated either on a Qubit 2.0 device (Invitrogen, Waltham, MA, USA) or a TapeStation (Agilent Technologies, Santa Clara, CA, USA).

RNA Analyses were carried out following the Archer® FusionPlex® Protocol (Version LA135.1, release 23^rd^ July 2020; https://dlongwood.com/wp-content/uploads/2019/12/Protocolo-FusionPlex®-Reactivos-para-Illumina®_pdf.pdf; last accessed on 5th August 2022). Briefly, the following steps were performed: random priming, first strand cDNA synthesis, second strand cDNA synthesis, PreSeq RNA QC assay, end repair, ligation, MBC adapter incorporation, ligation step 2, first and second PCR, library quantification on TapeStation (Agilent Technologies), preparation of libraries for MiSeq-loading by adaptation to 4 nM concentration, Sequencing on MiSeq (Illumina). Archer Suite Analysis v5.1.3 software (ArcherDX) was used for the computational analysis. Breakpoints were assessed and assigned to exonic or intronic positions assisted by the Integrative genomics viewer (Broad Institute and the Regents of the University of California; version IGV 2.13.x) [1]. A manual assessment of the relevance of confirmed genetic rearrangements was based on literature research and evaluation of the presence of relevant protein domains.

For Qiagen®-panels (Human Tumour Mutational Burden Panel (TMB) [DHS-6600Z] and Human Actionable Solid Tumour Panel (AST) [DHS-101Z]), the NGS QIAseq Targeted DNA Panel Handbook was used for reference (version August 2021; https://www.qiagen.com/nl/resources/resourcedetail?id=8907edbe-a462-4883-ae1b-2759657e7fd0&lang=en; last accessed on 5th August 2022). In brief, the following steps were performed sequentially: fragmentation, end-repair and A-addition, adapter ligation, clean-up of adapter-ligated DNA, target enrichment, clean-up of target enrichment, library quantification on a Qubit 2.0 instrument (Invitrogen), library adaptation to 4 nM concentration and sequencing on either a MiSeq or NextSeq500 device (Illumina).

For the TSO500 panel, a hybrid-capture-based method, the instructions of the TruSight Oncology 500 Reference Guide (Document # 1000000067621 v09; https://emea.support.illumina.com/content/dam/illumina-support/documents/documentation/chemistry_documentation/trusight/oncology-500/trusight-oncology-500-reference-guide-1000000067621-09.pdf; last accessed on 5th August 2022) were followed. To summarize the principle: unique molecular identifier (UMI)-containing adaptors were ligated to the DNA fragments. Afterward, a pool of oligos complementary to the genes included in the panel was hybridized into the DNA libraries. The probes, which were hybridized to the targeted regions, were captured with Streptavidin magnetic beads. Using primers, the enriched libraries were amplified. Next, purification with sample purification beads was performed. Pooling, denaturation, and dilution of the libraries ensued. TSO500 libraries were sequenced either on a NextSeq^TM^ 500 or 550Dx (Illumina).

Variant annotation was performed using Qiagen Clinical Insight (QCI) Interpret (Qiagen N.V.). The yielded annotations were cross-validated with publicly available databases, including ClinVar, COSMIC, and JAX CKB. Variants denoted as pathogenic or likely pathogenic by QCI were reported (only in rare exceptions also variants of unknown significance [VUS] were reported, e.g., if the clinicians had emphasized a specific gene). The software vcf2maf converter (https://github.com/mskcc/vcf2maf; last accessed 5^th^ August 2022) generated Mutation Annotation Format (MAF) files for the import to cBioportal (see above). In the case of the TSO500 panel, copy number variations (CNV) of ≥ 2.0 were considered potentially relevant and were further subjected to FISH analyses for validation (especially HER2 and MET with implications for recommendations). A tumour mutational burden (TMB) of ≥ 10.0 mutations/Mb was assessed as high according to the KEYNOTE-158 trial [2] and further discussed by the MTB. Generally, recommendations for colorectal cancer were not given with a TMB < 16 [3]. Panel-based MSI percentage was reported; however not deemed significant as a standalone parameter. Confirmation by PCR fragment sizing MSI analysis or mismatch repair protein deficiency (dMMR) was required.

**2.4.2 PCR fragment sizing MSI analysis**

4-6 ng of DNA, as determined on a Qubit 2.0 device (Invitrogen), were used for the PCR. The following primers were added:

- BAT 25-Up-6-FAM: 5’-TCG GCT CCA AGA ATG TAA GT-3’
- BAT 25-Down: 5’-TCT GCA TTT TAA CTA TGG CTC-3’
- BAT 26-Up-NED: 5’-TGA CTA CTT TTG ACT TCA GCC-3’
- BAT 26-Down: 5’-AAC CAT TCA ACA TTT TTA ACC C-3’
- APC –Up: ACT 5’-CAC TCT AGT GAT AAA TCG-3’
- APC –Down-Hex: 5’-ACT CAC TCT AGT GAT AAA TCG-3’
- D17S250-Up-NED (MFD15): 5’-GGA AGA ATC AAA TAG ACA AT-3’
- D17S250-Down (MFD15): 5’-GCT GGC CAT ATA TAT ATT TAA ACC-3’
- D2S123-Up-FAM: 5’-AAA CAG GAT GCC TGC CTT TA-3’
- D2S123-Down: 5’-GGA CTT TCC ACC TAT GGG AC-3’

Optionally, the panel was extended for the following primers:

- D18S61-Up: 5’- AAC CAA CAT AAT ATA GCA ATG G-3’
- D18S61-Down-HEX: 5’- TTC GAA CTT CGA ACC ACC C-3’
- TP53-ALS-Up-FAM: 5’- TCG AGG AGG TTG CAG TAA GCG GA-3’
- TP53-ALS-Down: 5’-AAC AGC TCC TTT AAT GGC AG-3’

The master mix for the PCR reaction was prepared as follows: 14.5 µl H_2_O, 1.5 µl DMSO, 1.8 µl MgC_l2_, 3.0 µl 10x Rx Buffer, 0.6 µl DNTPs (0.2 mM), 3 µl 10x primer multimix (individual primer concentrations between 0.2 and 0.5 μM) and 0.6 µl Taq-polymerase (0.1 U/µl). 25 µl of the master mix were added to 5.0 µl of template DNA (concentration 10-200 ng/µl). The employed cycling conditions were: 94°C 2 min/ 94°C 1 min, 60°C – 50°C 1 min (minus 1°C every cycle), 72°C 1 min for a total of 10 cycles/ 94°C 1 min, 50°C 1 min, 72°C for 25 cycles/ 72°C 8 min.

One µl of the PCR product was denatured for 3 min together with 12μl HiDi-Formamide und 0,7μl HD ROX 400 at 94°C. The ABI 3100/310 Genetic Analyzer (Applied Biosystems, Waltham, MA, USA) was used for fragment analysis. The MSI evaluation was based on comparing the colour and size of PCR products of tumour and normal tissue.

**2.4.3 Fluorescence in-situ hybridization**

Paraffin-embedded, formalin-fixed tissue sections were placed at 70°C for 10 min. Slides were deparaffinized through xylene (2 x 10 min) and 100% ethanol (5 min). A gradient of 96% and 70% of ethanol follows. A pepsin solution was applied for 20-30 min at 37 °C in a wet chamber. Subsequently, slides are washed in H_2_O, 96% and 70% ethanol, and left to dry. Next, slides are placed on a heating plate at 73°C for 10 minutes for denaturation. Hybridization was performed in a humidity chamber at 37 °C overnight with 20 µl of the ZytoLight SPEC ERBB2/CEN17 Dual Color Probe (ZytoVision GmbH, Bremerhaven, Germany) or the ZytoLight SPEC MET/CEN 7 Dual Color Probe (ZytoVision GmbH). A washing step with 1x Wash buffer for 5 min at room temperature ensued. After drying, slides were stained for DAPI. Fluorescence images were acquired on an IX73® fluorescent microscope (Olympus, Tokyo, Japan) with excitation and emission at appropriate wavelengths.

For HER2, categories were defined according to international standards [4]: Positive- HER2/CEN17-ratio ≥ 2.0 or on average ˃ 6 HER2-signals per nucleus; negative- HER2/CEN17-ratio < 2.0 or on average < 4 HER2-signals per nucleus; borderline- HER2/CEN17-ratio < 2.0 and 4-6 HER2-signals per nucleus.

For MET, these rules were applied following recognised standards [5]: positive (high-level amplification) if MET/CEN7-ratio ≥ 2.0 or average MET-signal number of ≥ 6.0 or ≥10% of tumour cells with ≥15 MET-signals.

**2.4.4 Immunohistochemistry**

Approximately 2 µm thick slices were cut and placed on warming plates with a temperature of about 67-71°C to melt the paraffin. Immunohistology was performed automatically with a BenchMark ULTRA system (Ventana/ Roche, Basel, Switzerland). The procedure is briefly summarized: first, an inhibitor of the endogenous peroxidase is applied. Next, pre-treatment with protease 1 (for EGFR) or pre-heating to 95°C and incubation in citrate buffer for up to 64 min (all other antibodies) is used for antigen retrieval. Primary antibodies (Table S2) are added automatically. Next, horseradish peroxidase (HRP)-coupled secondary antibodies are applied. DAB (3,3′-Diaminobenzidine) is oxidized by hydrogen peroxide in a reaction catalysed by HRP, forming a brown precipitate, which can be visualized using light microscopy. In addition, a counterstaining with haemalaun enhanced with a bluing solution is added.

**2.4.5 Statistical tests**

Baseline demographics were presented with descriptive statistics. Continuous data were described as median (range), and categorical variables were summarized as n (%). The Shapiro-Wilk test was employed to test continuous variables for normality. In addition, Fisher's exact test was performed to compare categorical variables between MTB and alternative therapy. In contrast, the Mann-Whitney U test was used for continuous variables, which tested negative for normal distribution.

Kaplan-Meier curves and Log-rank tests were used to compare OS between the groups with no therapy, MTB therapy, and alternative therapy and PFS between the groups with no therapy and alternative therapy for the entire cohort and the cholangiocarcinoma sub-cohort.

Statistical calculations were performed in GraphPad Prism version 9 (Graphpad Software, LLC, San Diego, CA, USA). *p* values were obtained in two-tailed tests, and *p* ≤ 0.05 was considered statistically significant.

**References**

[1] Robinson JT, Thorvaldsdóttir H, Winckler W, Guttman M, Lander ES, Getz G, et al. Integrative genomics viewer. Nat Biotechnol 2011;29:24–6. https://doi.org/10.1038/nbt.1754.

[2] Marabelle A, Fakih M, Lopez J, Shah M, Shapira-Frommer R, Nakagawa K, et al. Association of tumour mutational burden with outcomes in patients with advanced solid tumours treated with pembrolizumab: prospective biomarker analysis of the multicohort, open-label, phase 2 KEYNOTE-158 study. Lancet Oncol 2020;21:1353–65. https://doi.org/10.1016/S1470-2045(20)30445-9.

[3] Friedman CF, Hainsworth JD, Kurzrock R, Spigel DR, Burris HA, Sweeney CJ, et al. Atezolizumab Treatment of Tumors with High Tumor Mutational Burden from MyPathway, a Multicenter, Open-Label, Phase IIa Multiple Basket Study. Cancer Discov 2022;12:654–69. https://doi.org/10.1158/2159-8290.CD-21-0450.

[4] Wolff AC, Hammond MEH, Hicks DG, Dowsett M, McShane LM, Allison KH, et al. Recommendations for human epidermal growth factor receptor 2 testing in breast cancer: American Society of Clinical Oncology/College of American Pathologists clinical practice guideline update. J Clin Oncol Off J Am Soc Clin Oncol 2013;31:3997–4013. https://doi.org/10.1200/JCO.2013.50.9984.

[5] Schildhaus H-U, Schultheis AM, Rüschoff J, Binot E, Merkelbach-Bruse S, Fassunke J, et al. MET Amplification Status in Therapy-Naïve Adeno- and Squamous Cell Carcinomas of the Lung. Clin Cancer Res 2015;21:907–15. https://doi.org/10.1158/1078-0432.CCR-14-0450.
