# Supplementary material for: Critical evaluation of molecular tumour board outcomes following 2 years of clinical practice in a Comprehensive Cancer Centre
Source: Br J Cancer. 2022 Dec 26;128(6):1134–47. doi: 10.1038/s41416-022-02120-x (PMC10006213; doi:10.1038/s41416-022-02120-x)
Supplement: Supplementary file 2 — Supplementary Figures 1 and 2 captions [file 41416_2022_2120_MOESM2_ESM.docx]

**Figure S1. Alterations as a foundation for MTB recommendations (extension of Figure 1).** Oncoprint representation of alterations bearing relevance for MTB recommendations. Blue frames highlight individual alterations on which MTB recommendations were based for first-priority recommendations, and orange frames for lower-priority recommendations. The percentage of first- and lower-priority recommendations is given on the right. The order of patients can be translated to PatID using Table S3. The row “others” subsumes individual cases with BCL2 loss by IHC, androgen receptor expression, and FGF CNV.

MSI, microsatellite instability status; TMB, tumour mutational burden in mutations per megabase.

CUP, carcinoma of unknown primary; Ca, cancer; CCA, cholangiocarcinoma; GIST, gastrointestinal stromal tumour; HCC, hepatocellular carcinoma; HNSCC, head and neck squamous cell cancer; CRC, colorectal cancer; NEC, neuroendocrine carcinoma; RCC, renal cell cancer.

**Figure S2. Survival outcome measures in the cholangiocarcinoma subcohort.** Progression-free survival (*a*) and overall survival (*b*) of the cholangiocarcinoma cohort (intrahepatic, perihilar, and extrahepatic cholangiocarcinoma as well as gallbladder carcinoma combined) comparing patients receiving MTB recommended therapies, alternative therapies after the MTB and patients without further therapies. The *p*-value was calculated by the log-rank test.
