# Supplementary material for: Critical evaluation of molecular tumour board outcomes following 2 years of clinical practice in a Comprehensive Cancer Centre
Source: Br J Cancer. 2022 Dec 26;128(6):1134–47. doi: 10.1038/s41416-022-02120-x (PMC10006213; doi:10.1038/s41416-022-02120-x)
Supplement: Supplementary file 3 — Supplementary tables [file 41416_2022_2120_MOESM3_ESM.docx]

**Table S1:** NCT/DKTK evidence levels (table adapted from [1])

| **Level of evidence** | **Basis for classification** | **Tissue context** | **Description of classification** |
| --- | --- | --- | --- |
| m1A | Clinical data | Same histopathologic entity | The predictive value of the biomarker or clinical effectiveness of the corresponding drug in a molecularly stratified cohort was demonstrated in a prospective study or meta-analysis in the same tumour type. |
| m1B | Clinical data | Same histopathologic entity | The predictive value of the biomarker or clinical effectiveness of the drug in a molecularly stratified cohort was demonstrated in a retrospective cohort or case-control study in the same tumour type. |
| m1C | Clinical data | Same histopathologic entity | A case study or single unusual responder indicates that the biomarker is associated with response to the corresponding drug in the same tumour type. |
| m2A | Clinical data | Different histopathologic entity | The predictive value of the biomarker or clinical effectiveness of the corresponding drug in a molecularly stratified cohort was demonstrated in a prospective study or meta-analysis in a different tumour type. |
| m2B | Clinical data | Different histopathologic entity | The predictive value of the biomarker or clinical effectiveness of the drug in a molecularly stratified cohort was demonstrated in a retrospective cohort or case-control study in a different tumour type. |
| m2C | Clinical data | Different histopathologic entity | A case study or single unusual responder indicates that the biomarker is associated with response to the corresponding drug in a different tumour type. |
| m3 | Preclinical data | Not applicable | Preclinical data demonstrate that the biomarker predicts response to a specific drug, supported by a scientific rationale. |
| m4 | Biological rationale | Not applicable | A biological rationale exists that associates the biomarker with altered activity of cellular pathways/processes or drug sensitivity without direct clinical or preclinical evidence for a response to the drug. |

**Table S2:** List of antibodies

| **Name of antibody** | **Clone** | **Dilution** | **Order number** | **Company** |
| --- | --- | --- | --- | --- |
| AR | SP107 | 1:200 | 200R-15 | Cell Marque Corporation, Rocklin, CA, USA |
| Bcl-2 | 124 | 1:60 | M0887 | Dako/ Agilent Technologies |
| BRG1 (SNF2β) | EPNCIR 111A | 1:100 | Ab110641 | Abcam, Cambridge, United Kingdom |
| EGFR | E30 | 1:100 | M7239 | Dako/ Agilent Technologies, Santa Clara, CA, USA |
| HER2neu | Rabbit polyclonal | 1:500 | A0485 | Dako/ Agilent Technologies |
| MLH1 | G168-15 | 1:20 | 551092 | Becton, Dickinson and Company (BD), Franklin Lakes, NJ, USA |
| MSH2 | G219-1129 | 1:1 | 760-5093 | Ventana/ Roche, Basel, Switzerland |
| MSH6 | 44/MSH6 | 1:300 | 610919 | Becton, Dickinson and Company (BD) |
| PD-L1 | 22C3 | 1:50 | M3653 | Dako/ Agilent Technologies |
| PMS2 | A16-4 | 1:1 | 760-5094 | Ventana/ Roche |

**Table S3:** MTB treatment recommendations

| **Order in figure 1** | **PatID** | **Entity** | **Priority** | **Board Recommendation** | **Drug class** | **Alteration** | **EL** | **Literature** |
| --- | --- | --- | --- | --- | --- | --- | --- | --- |
| 1 | 205 | Invasive breast carcinoma | 1 | capivasertib + fulvestrant | AKT inhibitor | AKT1 gain | m1B | [2]; [3] |
|  |  |  | 2 | everolimus | mTOR inhibitor | AKT1 gain | m4 | / |
| 2 | 37 | primary mediastinal germ cell neoplasia | 1 | pembrolizumab | immune checkpoint inhibitor | PD-L1 | m1B | [4]; [5]; [6] |
| 3 | 43 | adenocarcinoma of esophagogastric junction | 1 | tastuzumab-deruxtecan | Her2 inhibitor | Her2 expression | m1A | [7] |
| 4 | 31 | malignant peritoneal mesothelioma | 1 | ALK inhibitor | ALK inhibitor | STRN-ALK fusion | m1C | [8,9] |
| 4 | 31 | malignant peritoneal mesothelioma (second discussion) | 1 | brigatinib | ALK inhibitor | STRN-ALK fusion + p.L1196M ALK resistance mutation | m3 | [10]; [11] |
|  |  |  | 2 | ceritinib | ALK inhibitor | STRN-ALK fusion + p.L1196M ALK resistance mutation | m3 | [12]; [13]; [14]; [15] |
|  |  |  | 3 | alectinib | ALK inhibitor | STRN-ALK fusion + p.L1196M ALK resistance mutation | m3 | [16] ;[17] |
| 5 | 39 | extragonadal germ cell tumor | 1 | ALK inhibitor | ALK inhibitor | DXH57-ALK | m2A | [18]; [19]; [20] |
| 6 | 2 | adenocarcinoma of esophagogastric junction (synchronous seminoma) | 1 | pembrolizumab | immune checkpoint inhibitor | PD-L1 | m1A | KEYNOTE-059: NCT03933449 |
|  |  |  | 2 | alpelisib | PI3K inhibitor | PIK3CA mutation | m2A | NCT01219699; [21] |
| 7 | 166 | NSCLC | 1 | trametinib + osimertinib | MEK inhibitor + EGFR inhibitor | AGK-BRAF fusion, EGFR exon 19 deletion | m1C | [22]; [23] |
|  |  |  | 2 | trametinib | MEK inhibitor | AGK-BRAF fusion | m1C | [24] |
| 8 | 13 | pCCA | 1 | binimetinib + capecitabine | MEK inhibitor | NRAS | m1C | [25]; [26] |
| 9 | 172 | NSCLC | 1 | capmatinib | MET inhibitor | MET | m1A | [27] |
|  |  |  | 2 | crizotinib | MET inhibitor | MET | m1A | [28]; [29] |
| 10 | 244 | iCCA | 1 | pembrolizumab (+ lenvatinib) | immune checkpoint inhibitor | MSI-high + high TMB | m1A | [30]; [31] |
|  |  |  | 2 | ivosidenib | IDH1 inhibitor | IDH1 gain | m1A | [32] ; [33] |
| 11 | 115 | Urothelial carcinoma | 1 | immune checkpoint inhibitor | immune checkpoint inhibitor | PD-L1 | m1A | / |
|  |  |  | 2 | alpelisib | PI3K inhibitor | PIK3CA gain | m2A | [21] |
| 12 | 52 | Prostate carcinoma | 1 | pembrolizumab + olaparib | immune checkpoint inhibitor + PARP inhibitor | PD-L1 + ATM | m3 | / |
|  |  |  | 2 | pembrolizumab | immune checkpoint inhibitor | PD-L1 | m1B | NCT02787005; NCT02054806; [34] |
|  |  |  | 3 | olaparib | PARP inhibitor | ATM loss | m1A | [35] ; [36] |
|  |  |  | 4 | rucaparib | PARP inhibitor | ATM loss | m1C | NCT02952534 |
| 13 | 5 | T-PLL | 1 | venetoclax + ibrutinib | BCL-2 inhibitor | BCL2 expression | m1C | [37]; NCT03873493 |
|  |  |  | 2 | tofacitinib + ruxolitinib | JAK inhibitor | JAK2 gain | m3 | [38]; [39]; [40] |
| 14 | 188 | Pancreatic acinar cell carcinoma | 1 | olaparib | PARP inhibitor | BRCA2 mutation | m1C | [41] |
| 15 | 114 | follicular T-cell lymphoma | 1 | 5-azacitidine | nucleoside analogue | TET2 loss | m1B | [42] |
| 16 | 6 | Dedifferentiated liposarcoma | 1 | palbociclib or abemaciclib | CDK4/6 inhibitor | CDK4 CNV | m1B | [43]; [44] ; [45] |
|  |  |  | 2 | pazopanib | tyrosine kinase inhibitor | FGFR1 + FGF5 CNV | m4 | [46] |
| 17 | 119 | adenocarcinoma of esophagogastric junction | 1 | crizotinib or entrectinib | ALK inhibitor | LACE1-ROS1 fusion | m2A | [47] |
|  |  |  | 2 | ibrutinib | tyrosine kinase inhibitor | MYC CNV | m3 | [48]; [49] |
| 18 | 3 | malignant solitary fibrous tumor (change of diagnosis from sarcomatoid carcinoma) | 1 | pazopanib or axitinib or regorafenib or sorafenib | tyrosine kinase inhibitor | FGF1, FGF2, FGF10 CNV | m4 | [50] ; [51] |
| 19 | 198 | NSCLC | 1 | crizotinib + trastuzumab | Her2 inhibitor + MET inhibitor | Her2- and MET-amplification | m2C | [52] ;[53] |
|  |  |  | 2 | trastuzumab-deruxtecan | Her2 inhibitor | Her2 amplification | m1B | [54]; [55] |
| 20 | 93 | HNSCC | 1 | alpelisib + cetuximab | PI3K inhibitor | PIK3CA mutation | m3 | [56] |
|  |  |  | 2 | temsirolimus + cetuximab + bevacizumab | mTOR- inhibitor + EGFR inhibitor + VEGF inhibitor | PIK3CA mutation | m3 | [57] |
|  |  |  | 3 | temsirolimus + carboplatin + paclitaxel | mTOR inhibitor | PIK3CA | m3 |  |
| 21 | 33 | pCCA | 1 | pembrolizumab | immune checkpoint inhibitor | MSI-high | m1A | FDA approval second line treatment |
| 22 | 9 | Uterine leiomyosarcoma | 1 |  | immune checkpoint inhibitor | POLE loss | m2C | [58]; [59]; [60] |
| 23 | 48 | CRC | 1 | alpelisib (+ fulvestrant) | PI3K inhibitor | PIK3CA mutation | m1B | NCT01219699; [61]; [21]; NCT01387321 |
| 24 | 40 | Thymoma B3 | 1 | pembrolizumab | immune checkpoint inhibitor | PD-L1 | m1B | [62]; [63] |
| 25 | 42 | Malignant melanoma | 1 | imatinib | tyrosine kinase inhibitor | KIT gain | m1A | [64] |
|  |  |  | 2 | nilotinib or dasatinib | Kinase inhibitor | KIT gain | m1A | [65] |
| 26 | 249 | iCCA | 1 | EGFR inhibitor (+ bevacizumab) | EGFR inhibitor | EGFR amplification | m2B | [66]; [67] |
| 27 | 216 | iCCA | 1 | infigratinib | FGFR inhibitor | FGFR gain | m1C | [68] |
|  |  |  | 2 | pemigatinib | FGFR inhibitor | FGFR2 gain | m1C | [69] ; [70] |
|  |  |  | 3 | lenvatinib + pembrolizumab | tyrosine kinase inhibitor + immune checkpoint inhibitor | FGFR2 | m1C | [71] |
| 28 | 238 | gall bladder carcinoma | 1 | trametinib | MEK inhibitor | BRAF class 2 mutation | m2B | [72]; [73]; [74] |
| 29 | 14 | Salivary carcinoma, NOS | 1 | pembrolizumab | immune checkpoint inhibitor | TMB-high, MSI-high | m2A | [75]; [76] |
|  |  |  | 2 | alpelisib (+ androgen blockade) | PI3K inhibitor | PIK3CA | m2A | [77] |
|  |  |  | 3 | olaparib (sequentially after platinum containing CTx) | PARP inhibitor | BRCA2 | m2A | [78]; [35] |
|  |  |  | 4 | mTOR inhibitor | mTOR inhibitor | TSC2 | m2C | [79] |
| 30 | 159 | CRC | 1 | nivolumab | immune checkpoint inhibitor | MUTYH loss | m1C | [80] |
| 31 | 214 | Pancreatic ductal carcinoma | 1 | sotorasib | KRAS Inhibitor | KRAS | m2A | NCT04303780 |
| 32 | 44 | gall bladder carcinoma | 1 | pembrolizumab | immune checkpoint inhibitor | CPS | m2C | [81]; [82] |
| 33 | 163 | CRC | 1 | immune checkpoint inhibitor | immune checkpoint inhibitor | TMB-high | m1C | [83] ; [84]; [85] |
| 34 | 7 | iCCA | 1 | ivosidenib | IDH1 inhibitor | IDH1 gain | m1A | [32] ; [33] |
|  |  |  | 2 | olaparib | PARP inhibitor | BAP1, IDH1, ARID1A mutation | m3 | [86] |
| 35 | 133 | Adeno CUP | 1 | erlotinib + MET inhibitor | EGFR inhibitor + MET inhibitor | EGFR fusion + MET amplification | m3 | [87]; [88]; [89]; [27] |
| 36 | 164 | high grade serous carcinoma | 1 | T-DM1 + neratinib | Her2 inhibitor | Her2 gain | m2C | [90] |
|  |  |  | 2 | trastuzumab-deruxtecan | Her2 inhibitor | Her2 gain | m3 | [90] |
| 37 | 21 | gastric carcinoma | 1 |  | immune checkpoint inhibitor | PD-L1, TMB-Intermediate | m2A | [91]; [92] |
|  |  |  | 2 | trastuzmab-deruxtecan | Her2 inhibitor | Her2 amplification | m1A | [7] ; NCT0361526 |
|  |  |  | 3 | crizotinib | MET inhibitor | MET amplification | m1B | [93]; [94] |
| 38 | 27 | iCCA | 1 | pemigatinib | FGFR inhibitor | FGFR2-PHGDH fusion | m1A | [70] |
|  |  |  | 2 | Inclusion to NCT3797326 trial (lenvatinib + pembrolizumab) | immune checkpoint inhibitor | FGFR2-PHGDH fusion | m1C | NCT03797326 |
| 39 | 16 | iCCA | 1 | olaparib | PARP inhibitor | BRCA2 | m1C | [78]; [35]; [31] |
|  |  |  | 2 | everolimus | mTOR inhibitor | TSC1 | m2C | [95] |
| 40 | 86 | CRC | 1 | mTOR inhibitor (+ irinotecan) | mTOR inhibitor | mTOR gain | m2C | [96] ; [97]; [98] |
| 41 | 141 | HCC | 1 | atezolizumab + bevacizumab | immune checkpoint inhibitor | PD-L1 | m4 | [99]; NCT03434379; NCT 04224636 |
| 42 | 10 | Salivary duct carcinoma | 1 | androgen deprivation therapy | Androgen blockade | Androgen receptor expression | m1A | [100] |
|  |  |  | 2 | trastuzumab-deruxtecan | Her2 inhibitor | Her2 expression | m2B | [101]; [102] |
| 43 | 228 | iCCA | 1 | olaparib | PARP inhibitor | ATM loss | m2C | [103]; [104]; [35] |
| 44 | 64 | Adeno CUP | 1 | olaparib | PARP inhibitor | ATM loss | m2A | [35]; [36] |
| 45 | 202 | CRC | 1 | regorafenib | PDGFR inhibitor | PDGFRB-fusion | m4 | / |
| 46 | 75 | iCCA | 1 | crizotinib | tyrosine kinase inhibitor | MET-fusion | m1C | [105] |
|  |  |  | 2 | capmatinib | MET inhibitor | MET-fusion | NA | [27] |
| 47 | 8 | dCCA | 1 | dabrafenib + trametinib | RAF inhibitor + MEK inhibitor | BRAF gain | m2C | [106]; [107] |
| 48 | 130 | pancreatic ductal carcinoma | 1 | palbociclib + trametinib | CDK4/6 inhibitor + MEK inhibitor | CDKN2A loss + KRAS gain | m1C | [108]; [109] |
| 49 | 129 | chromophobe renal cell carcinoma | 1 |  | Genotoxic chemotherapy | MRE11 mutation | m3 | [110] ; [111]; [112] |
| 50 | 121 | TNBC | 1 | alpelisib + nab-paclitaxel | PI3K inhibitor | PIK3CA gain | m1B | [21]; [113] ; [114] |
| 51 | 125 | malignant melanoma | 1 | PI3K inhibitor + RAF inhibitor | PI3K inhibitor + RAF inhibitor | BRAF V600K gain + PIK3CA gain | m1C | [115] |
| 52 | 47 | urothelial carcinoma | 1 | trastuzumab + pertuzumab | Her2 inhibitor | Her2 amplification | m1B | [116] |
|  |  |  | 2 | lapatinib | EGFR- + Her2 Inihibitor | Her2 amplification | m1C | [117]; [118] |
|  |  |  | 3 |  | HDAC inhibitor | CREBBP loss | m1C | [119] |
| 53 | 4 | papillary thyroid carcinoma | 1 | pembrolizumab | immune checkpoint inhibitor | MSI-high | m1A | [75] |
| 54 | 191 | gastric carcinoma | 1 | pembrolizumab (+ lenvatinib) | immune checkpoint inhibitor | PD-L1 + TMB-high | m1A | [120]; [121]; [85] |
| 55 | 153 | HNSCC | 1 | cetuximab + alpelisib | PI3K inhibitor + EGFR inhibitor | PIK3CA gain | m1C | [122]; NCT01602315 |
| 56 | 189 | sebaceous carcinoma | 1 | rucaparib | PARP inhibitor | RAD51C loss | m2C | [123]; [124] |
| 57 | 239 | iCCA | 1 | lenvatinib + pembrolizumab | immune checkpoint inhibitor | FGFR2 gain | m1C | [71] |
|  |  |  | 2 | pazopanib | tyrosine kinase inhibitor | FGFR2 gain | m1C | [125] |
|  |  |  | 3 | erdafitinib | FGFR inhibitor | FGFR2 gain | m1C | [126] |
|  |  |  | 4 | infigratinib | FGFR inhibitor | FGFR2 gain | m1C | [[127]](https://doi.org/10.1200/PO.17.00018%20;%20doi:10.1200/JCO.2017.75.5009%20(unwirksam)%20;%20PMID:29182496) |
| 58 | 22 | iCCA | 1 | Inclusion to clinical trial (pembrolizumab + lenvatinib) | immune checkpoint Inihibitor |  | NA | NCT03797326 |
|  |  |  | 2 | pemigatinib | FGFR inhibitor | FGFR2-BICC fusion | m1A | [70] |
| 59 | 251 | iCCA | 1 | ivosidenib | IDH1 inhibitor | IDH1 gain | m1A | [32]; [33] |
|  |  |  | 2 | binimetinib + capecitabine | MEK inhibitor | NRAS gain | m1C | [25] |
|  |  |  | 3 | olaparib | PARP inhibitor | BAP1 loss + IDH1 gain | m2C | [33]; [128] |
| 60 | 162 | Esophageal squamous carcinoma | 1 |  | immune checkpoint inhibitor | TMB-intermediate | m2A | [85]; [129] |
| 61 | 182 | Sebaceous carcinoma | 1 | trastuzumab-deruxtecan | Her2 inhibitor | Her2 amplification | m2A | [130] ; [55] |
|  |  |  | 2 | trastuzumab + pertuzumab or trastuzumab + lapatinib | Her2 inhibitor | Her2 amplification | m2A | [131] ; [132] |
|  |  |  | 3 | trastuzumab-emtansine | Her2 inhibitor | Her2 amplification | m2A | [133] |
| 62 | 94 | eCCA | 1 | trastuzumab + pertuzumab (+ CTx) | HER2 inhibitor | Her2 amplification | m1B | [134] |
|  |  |  | 2 | zanidatamab | HER2 inhibitor | HER2 amplification | m1B | [135] |
|  |  |  | 3 | trastuzumab + CTx | Her2-Inibitor | Her2 amplification | m1C | [136] |
| 63 | 46 | CRC | 1 | trastuzumab-deruxtecan | Her2 inhibitor | Her2 amplification | m1B | [137] |
|  |  |  | 2 | trastuzumab + lapatinib or trastuzumab + pertuzumab | Her2 inhibitor | Her2 amplification | m1B | [131] |
|  |  |  | 3 | Trastuzumab-emtansine | Her2 inhibitor | Her2 amplification | m1C | [138] |
| 64 | 97 | adenocarcinoma of esophagogastric junction | 1 | tastuzumab-deruxtecan | Her2 inhibitor | Her2 amplification | m1A | [7] |
|  |  |  | 2 | trastuzumab + lapatinib | Her2 inhibitor | Her2 amplification + Her3 mutation | m2C | [139]; [140] |
|  |  |  | 3 | trastuzumab + afatinib | Her2 inhibitor + Her3 inhibitor | Her2 amplification + Her3 mutation | m2C | [139] |
| 65 | 203 | Invasive breast carcinoma | 1 | alpelisib (+ fulvestrant) | PI3K inhibitor | PIK3CA gain | m1A | [21] |
| 66 | 38 | pCCA | 1 | pembrolizumab | immune checkpoint inhibitor | MSI-H, dMMR | m1A | [75]; [76] |
|  |  |  | 2 | olaparib (+ pembrolizumab) | PARP inhibitor | BRCA2 | m2C | [141]; [142] |
| 67 | 161 | papillary thyroid carcinoma | 1 | BRAF inhibitor | RAF inhibitor | BRAF V600E gain | m1A | [143]; [144]; [145] |
|  |  |  | 2 | BRAF inhibitor + MEK inhibitor | RAF inhibitor + MEK inhibitor | BRAF V600E gain | m1B | [146] |
|  |  |  | 3 | BRAF inhibitor + radioactive iodine | RAF inhibitor | BRAF 600E gain | m1B | [147]; [148] |
| 68 | 225 | pCCA | 1 | trametinib (+ dabrafenib) | MEK inhibitor | BRAF | m2C | [149]; [72] ; [73] ; [74]; [150] |
| 69 | 23 | NSCLC | 1 | EGFR-TKI | EGFR inhibitor | EGFR kinase domain duplication | m1C | [151]; [152]; [153]; [154] |
| 70 | 87 | iCCA | 1 | erdafitinib | FGFR inhibitor | FGFR3 mutation | m1C | [155]; [156] |
| 71 | 54 | pancreatic ductal carcinoma | 1 | olaparib | PARP inhibitor | BRCA1 mutation | m1B | [78] |
| 72 | 201 | CRC | 1 | trastuzumab-deruxtecan | Her2 inhibitor | Her2 amplification | m1B | [137] |
|  |  |  | 2 | trastuzumab + pertuzumab or trastuzumab + lapatinib | Her2 inhibitor | Her2 amplification | m1B | [131]; [132] |
| 73 | 210 | HNSCC (synchronous maxillary chondroblastic osteosarcoma) | 1 | pembrolizumab (+ CTx) | immune checkpoint inhibitor | PD-L1 | m1A | [157] |
|  |  |  | 2 | olaparib (+ trabectedin) | PARP inhibitor | BRCA2 loss | m3 | [78] ; [35] |
| 74 | 99 | T-LBL | 1 | ruxolitinib | JAK inhibitor | STAT5B | m2B | [158]; [159] |
| 75 | 219 | iCCA | 1 | trastuzumab + pertuzumab | HER2 inhibitor | Her2 amplification | m1B | [160] ; [161] |
| 76 | 73 | Esophageal squamous carcinoma | 1 | erlotinib (+ cetuximab) | Her2 inhibitor and EGFR inhibitor | EGFR CNV | m2C | [162] |
|  |  |  | 2 | necitumumab + CTx | EGFR inhibitor | EGFR CNV | m2A | [163] |
|  |  |  | 3 | icotinib | EGFR inhibitor | EGFR amplification | m1B | [164] |
| 77 | 135 | Adeno CUP | 1 | inclusion to trial NCT04111458 | pan-KRAS inhibitor | KRAS gain | NA | NCT04111458 |
| 78 | 179 | inflammatory pseudotumor of the lung | 1 |  | immune checkpoint inhibitor | PD-L1 (ICS) | m2A | [165] |
| 79 | 220 | eCCA | 1 | olaparib | PARP inhibitor | CHEK2 | m2C | [166]; [35]; [36] |
| 80 | 250 | pCCA | 1 | binimetinib + capecitabine | MEK inhibitor | KRAS gain | m1C | [25] |
|  |  |  | 2 | olaparib | PARP inhibitor | CHEK2 loss | m2C | [166] |
| 81 | 233 | iCCA | 1 | pemigatinib | FGFR inhibitor | FGFR2-NOL4 fusion | m1A | [70] |
| 82 | 183 | esophageal adenocarcinoma | 1 | ivosidenib | IDH1 inhibitor | IDH1 gain | m2A | [32]; [33]; [167] |
| 83 | 36 | GIST | 1 | regorafenib | tyrosine kinase inhibitor | KIT gain | m1A | [168] |
|  |  |  | 2 | other TKIs | tyrosine kinase inhibitor | KIT gain | m1A | [169] ; [170]; [171] |
| 84 | 131 | adenocarcinoma of esophagogastric junction | 1 |  | immune checkpoint inhibitor | SMARCA4 loss | m2B | [172] ; [173] |
| 85 | 80 | NSCLC | 1 | crizotinib | MET inhibitor | MET fusion | m1C | [174] ; [175]; [176] |
|  |  |  | 2 | capmatinib | MET inhibitor | MET fusion | m1C | [27] |
| 86 | 29 | gastric carcinoma | 1 | pembrolizumab | immune checkpoint inhibitor | PD-L1 + MSI | m1A | KEYNOTE-059: NCT03933449 |
|  |  |  | 2 | alpelisib | PI3K inhibitor | PIK3CA mutation | m1A | NCT31091374 |
| 87 | 212 | iCCA | 1 | pemigatinib | FGFR inhibitor | FGFR2-NCD80 fusion | m1A | [69]; [177] |
|  |  |  | 2 | infigratinib | FGFR inhibitor | FGFR2-NCD80 | m1B | [127] |
|  |  |  | 3 | levantinib (+pembrolizumab/ nivolumab) | tyrosine kinase inhibitor + immune checkpoint inhibitor | FGFR2-NCD80 | m1B | [71] |
|  |  |  | 4 | erdafitinib | FGFR inhibitor | FGFR2-NCD80 | m1B | [155] |
| 88 | 34 | Adeno CUP | 1 | CDK4/6 inhibitor | CDK4/6 inhibitor | SMARCA4, KRAS | m3 | NCT02022982, NCT03170206, NCT02152631, NCT04165031 |
|  |  |  | 2 | nivolumab | immune checkpoint inhibitor | SMARCA4, KRAS + TP53, PD-L1 (CPS 20), TMB | m2B | [178] |
|  |  |  | 3 | tazemetostat | EZH2 inhibitor | SMARCA4 | m2A | NCT02601950 |
| 89 | 120 | CRC | 1 | pralsetinib (inclusion to trial NCT03037385) | RET inhibitor | RET mutation | m2B | NCT03007385 |
|  |  |  | 2 | selpercatinib | RET inhibitor | RET mutation | m2B | [179] |
| 90 | 218 | pCCA | 1 | pembrolizumab + lenvatinib | immune checkpoint inhibitor | MSI-high | m1A | [180] |
|  |  |  | 2 |  | PARP inhibitor | PALB2 | m2A | [35] |
| 91 | 90 | urothelial carcinoma | 1 | pertuzumab + trastuzumab + docetaxel | Her2 inhibitor | Her2 amplification | m1B | [116] |
|  |  |  | 2 | trastuzumab-emtansin | Her2 inhibitor | Her2 amplification | m1B | NCT02999672 |
|  |  |  | 3 | afatinib | tyrosine kinase inhibitor | Her2 amplification | m1B | NCT02780687 |
| 92 | 151 | adenocarcinoma of esophagogastric junction | 1 |  | immune checkpoint inhibitor | PD-L1 | m1A | NCT02872116; [181]; [182] |
| 93 | 149 | malignant melanoma | 1 | trastuzumab + lapatinib (+MEK inhibitor) | Her2 inhibitor (+ MEK inhibitor) | ERBB3 gain + ERBB3 CNV | m2C | [139]; [140]; [183] |
|  |  |  | 2 | abemaciclib | CDK4/6 inhibitor | CDK4 CNV | NA | [184] |
| 94 | 241 | iCCA | 1 | trastuzumab + pertuzumab | HER2 inhibitor | Her2 amplification | m1C | / |
|  |  |  | 2 |  | immune checkpoint inhibitor | POLE mutation | m2A | / |
| 95 | 85 | Adeno CUP | 1 | alpelisib + fulvestrant | PI3K inhibitor | PIK3CA | m2A | [21] |
|  |  |  | 2 |  | FGFR inhibitor | FGFR2-ABLIM-fusion | m2A | [156] |
| 96 | 20 | endometrioid adenocarcinoma | 1 | alpelisib + fulvestrant | PI3K inhibitor + estrogen blockade | PIK3CA gain | m2A | [21] |
| 97 | 30 | iCCA | 1 | pemigatinib | FGFR inhibitor | FGFR2-MYH16 fusion | m1A | [70] |
|  |  |  | 2 | Inclusion to trial NCT03230318 (derazantinib) | FGFR inhibitor | FGFR2-MYH16 fusion | m1B | NCT03230318; [185] |
|  |  |  | 3 | Inclusion to trial NCT03797326 (pembrolizumab + lenvatinib) | tyrosine kinase inhibitor + immune checkpoint inhibitor | FGFR2-MYH16 fusion | m1C | NCT03797326; [71] |
| 98 | 58 | uterine leiomyosarcoma | 1 | Inclusion to trial NCT04083976 | FGFR inhibitor | FGFR2 gain | m3 | NCT04083976; [186] |
| 99 | 15 | nonseminomatous germ cell tumor | 1 | pembrolizumab | immune checkpoint inhibitor | PD-L1 | m1B | [4]; [5] ; [6] |
| 100 | 156 | urothelial carcinoma | 1 | erdafitinib | FGFR inhibitor | FGFR3 mutation | m1A | [156] |
|  |  |  | 2 | pemigatinib | FGFR inhibitor | FGFR3 mutation | m1A | NCT02872714 |
| 101 | 25 | peripheral T-cell lymphoma | 1 | azacitidine (+ ascorbate) | nucleoside analogue | TET2 loss | m1B | [42]; [187]; [188] |
| 102 | 102 | CRC | 1 | Inclusion to trial NCT02576444 | PARP inhibitor | TP53 loss | NA | NCT02576444 |
| 103 | 12 | dedifferentiated chondrosarcoma | 1 | pazopanib + trametinib | MEK inhibitor | NRAS gain | m1C | [189] |
|  |  |  | 2 | pembrolizumab | immune checkpoint inhibitor | PD-L1 | m1C | [190] |
| 104 | 145 | GIST | 1 | regorafenib | tyrosine kinase inhibitor | KIT exon 17 mutation | m1B | [168] |
|  |  |  | 2 | pazopanib or nilotinib | tyrosine kinase inhibitor | / | m1B | [169]; [171] |
|  |  |  | 3 | sorafenib or dasatinib | tyrosine kinase inhibitor | / | m1B | [170] |
| 105 | 232 | pCCA | 1 | binimetinib + capecitabine | MEK inhibitor | KRAS gain | m1C | [25] |
| 106 | 11 | leiomyosarcoma | 1 | selpercatinib | RET inhibitor | RET gain | m2A | [191]; [192] |
|  |  |  | 2 | pralsetinib | RET inhibitor | RET gain | m2A | NCT03037385 |
|  |  |  | 3 | cabozantinib | RET inhibitor | RET gain | m2A | [193] |
| 107 | 168 | adenoid cystic carcinoma | 1 | trastuzumab-deruxtecan | Her2 inhibitor | Her2 expression | NA | [102] |
| 108 | 70 |  | 1 | alpelisib + CTx | PI3K inhibitor | PIK3CA gain | m2A | [21] |
| 109 | 45 | CUP | 1 |  | immune checkpoint inhibitor | PD-L1 | m2A | NCT04131621 |
|  |  |  | 2 | palbociclib | CDK4/6 inhibitor | CDK4 CNV | m2B | [44] |
| 110 | 234 | eCCA | 1 | pembrolizumab | immune checkpoint inhibitor | dMMR/MSI | m1A | [75]; [76] |
| 111 | 194 | gastric carcinoma | 1 | crizotinib | MET inhibitor | MET amplification | m1B | [194] |
| 112 | 139 | TNBC | 1 | atezolizumab + nab-paclitaxel | immune checkpoint inhibitor | PD-L1 | m1A | [195] |
|  |  |  | 2 | crizotinib | MET inhibitor | MET amplification | m1C | [196]; [197] |
|  |  |  | 3 | trastuzumab-deruxtecan | HER2 inhibitor | Her2 expression | m1B | [101]; NCT03734029 |
| 113 | 1 | NSCLC | 1 | afatinib + paclitaxel | Her2 inhibitor | ERBB2 gain | m1C | [198]; [199] |
|  |  |  | 2 | afatinib + crizotinib | Her2 inhibitor + MET inhibitor | ERBB2 gain + MET expression | m1C | [200] |
|  |  |  | 3 | T-DM1 | Her2 inhibitor | ERBB2 gain | m1A/B | [201] |
| 114 | 32 | neuroendocrine carcinoma of the breast | 1 | palbociclib (+ fulvestrant) | CDK4/6 inhibitor + estrogen blockade | SMARCA4 mutation | m4 | [202] |
|  |  |  |  | tazemetostat | EZH2 inhibitor | SMARCA4 mutation | m2A | NCT02601950 |
| 115 | 35 | Salivary carcinoma, NOS | 1 | trastuzumab-emtansine | Her2 inhibitor | Her2 amplification | m2B | [203] |
|  |  |  | 2 | trastuzumab-deruxtecan | Her2 inhibitor | Her2 expression | m2B | [55] |
|  |  |  | 3 | combined androgen blockade | Androgen blockade | androgen receptor expression | m1C | [100] |
|  |  |  | 3 | pembrolizumab | immune checkpoint inhibitor | PD-L1 | m1B | [204] |
| 116 | 96 | mixed HCC/CCC | 1 | alpelisib + (platinum-containing) CTx | PI3K inhibitor | PIK3CA gain | m2A | [205] ; [206] |
| 117 | 41 | adenocarcinoma of esophagogastric junction | 1 |  | immune checkpoint inhibitor | PD-L1 | NA | NCT03443856 |
| 118 | 17 | iCCA | 1 |  | immune checkpoint inhibitor | POLD1 loss | m1B | [59] |
| 119 | 18 | gall bladder carcinoma | 1 |  | immune checkpoint inhibitor | POLE loss | m2C | [58]; [59] |
| 120 | 19 | NSCLC | 1 | mobocertinib | EGFR inhibitor | EGFR exon 20 | m1A | [207] |
|  |  |  | 2 | afatinib + cetuximab | EGFR inhibitor | / | m3 |  |
| 121 | 24 | eCCA | 1 | alpelisib + cisplatin + gemcitabine | PI3K inhibitor | PIK3CA gain | m4 | NCT 03941782 |
|  |  |  | 2 |  | BET inhibitor | c-Myc overexpression | m3 | [208] ; [209]; [210] |
| 122 | 72 | iCCA | 1 |  | IDH2 inhibitor | IDH2 | m2A | [211] |
| 123 | 26 | iCCA | 1 | ivosidenib | IDH1 inhibitor | IDH1 gain | m1A | NCT02989857 |
| 124 | 28 | Adeno CUP | 1 | trametinib + dabrafenib | Raf inhibitor + MEK inhibitor | BRAF V600E gain | m2A | [212] |
| 125 | 108 | Salivary duct carcinoma | 1 |  | Her2 inhibitor | Her2 expression | m1B | [213], [133], [214], [215] |
|  |  |  | 2 |  | Androgen blockade | androgen receptor expression | m1B | [100] |
|  |  |  | 3 |  | PI3K inhibitor | PIK3CA | m2A | [205] |
|  |  |  | 4 |  | PARP inhibitor | ATR | m4 | [216] |

NSCLC, Non-small-cell lung cancer; pCCA, perihilar cholangiocarcinoma; iCCA intrahepatic cholangiocarcinoma; dCCA, distal cholangiocarcinoma; T-PLL, T-Cell Prolymphocytic Leukemia; T-LBL, T-cell lymphoblastic lymphoma; HNSCC, head and neck squamous cell carcinoma; CRC, colorectal carcinoma; HCC, hepatocellular carcinoma; TNBC, triple negative breast cancer; CUP, carcinoma of unknown primary; GIST, gastrointestinal stromal tumor; gain, gain-of-function mutation; loss, loss-of-function mutation; NA, not applicable; PatID, patient ID; EL, evidence level.

**Literature:**

[1] Horak P, Heining C, Kreutzfeldt S, Hutter B, Mock A, Hüllein J, et al. Comprehensive Genomic and Transcriptomic Analysis for Guiding Therapeutic Decisions in Patients with Rare Cancers. Cancer Discov 2021;11:2780–95. https://doi.org/10.1158/2159-8290.CD-21-0126.

[2] Smyth LM, Tamura K, Oliveira M, Ciruelos EM, Mayer IA, Sablin M-P, et al. Capivasertib, an AKT kinase inhibitor, as monotherapy or in combination with fulvestrant in patients with AKT1 E17K-mutant, ER-positive metastatic breast cancer. Clin Cancer Res 2020;26:3947–57. https://doi.org/10.1158/1078-0432.CCR-19-3953.

[3] Jones RH, Casbard A, Carucci M, Cox C, Butler R, Alchami F, et al. Fulvestrant plus capivasertib versus placebo after relapse or progression on an aromatase inhibitor in metastatic, oestrogen receptor-positive breast cancer (FAKTION): a multicentre, randomised, controlled, phase 2 trial. Lancet Oncol 2020;21:345–57. https://doi.org/10.1016/S1470-2045(19)30817-4.

[4] Zschäbitz S, Lasitschka F, Hadaschik B, Hofheinz R-D, Jentsch-Ullrich K, Grüner M, et al. Response to anti-programmed cell death protein-1 antibodies in men treated for platinum refractory germ cell cancer relapsed after high-dose chemotherapy and stem cell transplantation. Eur J Cancer 2017;76:1–7. https://doi.org/10.1016/j.ejca.2017.01.033.

[5] Goldfarb JA, Dinoi G, Mariani A, Langstraat CL. A case of multi-agent drug resistant choriocarcinoma treated with Pembrolizumab. Gynecol Oncol Rep 2020;32:100574. https://doi.org/10.1016/j.gore.2020.100574.

[6] Clair KH, Gallegos N, Bristow RE. Successful treatment of metastatic refractory gestational choriocarcinoma with pembrolizumab: A case for immune checkpoint salvage therapy in trophoblastic tumors. Gynecol Oncol Rep 2020;34:100625. https://doi.org/10.1016/j.gore.2020.100625.

[7] Shitara K, Bang Y-J, Iwasa S, Sugimoto N, Ryu M-H, Sakai D, et al. Trastuzumab deruxtecan in previously treated HER2-positive gastric cancer. N Engl J Med 2020;382:2419–30. https://doi.org/10.1056/NEJMoa2004413.

[8] Gerthofer V, Scheiter A, Lüke F, Keil F, Utpatel K, Pöhmerer L-M-G, et al. STRN-ALK Fusion in a Case of Malignant Peritoneal Mesothelioma: Mixed Response to Crizotinib, Mode of Resistance, and Brigatinib Sequential Therapy. JCO Precis Oncol 2021:1507–13. https://doi.org/10.1200/PO.21.00184.

[9] Rüschoff JH, Gradhand E, Kahraman A, Rees H, Ferguson JL, Curioni-Fontecedro A, et al. STRN -ALK Rearranged Malignant Peritoneal Mesothelioma With Dramatic Response Following Ceritinib Treatment. JCO Precis Oncol 2019;3. https://doi.org/10.1200/PO.19.00048.

[10] Huber RM, Hansen KH, Paz-Ares Rodríguez L, West HL, Reckamp KL, Leighl NB, et al. Brigatinib in crizotinib-refractory ALK+ NSCLC: 2-year follow-up on systemic and intracranial outcomes in the phase 2 ALTA trial. J Thorac Oncol 2020;15:404–15. https://doi.org/10.1016/j.jtho.2019.11.004.

[11] Zhang S, Anjum R, Squillace R, Nadworny S, Zhou T, Keats J, et al. The potent ALK inhibitor brigatinib (AP26113) overcomes mechanisms of resistance to first- and second-generation ALK inhibitors in preclinical models. Clin Cancer Res 2016;22:5527–38. https://doi.org/10.1158/1078-0432.ccr-16-0569.

[12] Crinò L, Ahn M-J, De Marinis F, Groen HJM, Wakelee H, Hida T, et al. Multicenter phase II study of whole-body and intracranial activity with ceritinib in patients with ALK-rearranged non–small-cell lung cancer previously treated with chemotherapy and crizotinib: Results from ASCEND-2. J Clin Oncol 2016;34:2866–73. https://doi.org/10.1200/jco.2015.65.5936.

[13] Shaw AT, Kim D-W, Mehra R, Tan DSW, Felip E, Chow LQM, et al. Ceritinib in ALK-rearranged non-small-cell lung cancer. N Engl J Med 2014;370:1189–97. https://doi.org/10.1056/NEJMoa1311107.

[14] Gainor JF, Tan DSW, De Pas T, Solomon BJ, Ahmad A, Lazzari C, et al. Progression-free and overall survival in ALK-positive NSCLC patients treated with sequential crizotinib and ceritinib. Clin Cancer Res 2015;21:2745–52. https://doi.org/10.1158/1078-0432.CCR-14-3009.

[15] Friboulet L, Li N, Katayama R, Lee CC, Gainor JF, Crystal AS, et al. The ALK inhibitor ceritinib overcomes crizotinib resistance in non-small cell lung cancer. Cancer Discov 2014;4:662–73. https://doi.org/10.1158/2159-8290.CD-13-0846.

[16] Nakanishi Y, Masuda S, Iida Y, Takahashi N, Hashimoto S. Case report of non-small cell lung cancer with STRN-ALK translocation: A nonresponder to alectinib. J Thorac Oncol 2017;12:e202–4. https://doi.org/10.1016/j.jtho.2017.08.009.

[17] Sakamoto H, Tsukaguchi T, Hiroshima S, Kodama T, Kobayashi T, Fukami TA, et al. CH5424802, a selective ALK inhibitor capable of blocking the resistant gatekeeper mutant. Cancer Cell 2011;19:679–90. https://doi.org/10.1016/j.ccr.2011.04.004.

[18] Kwak EL, Bang Y-J, Camidge DR, Shaw AT, Solomon B, Maki RG, et al. Anaplastic lymphoma kinase inhibition in non-small-cell lung cancer. N Engl J Med 2010;363:1693–703. https://doi.org/10.1056/NEJMoa1006448.

[19] Peters S, Camidge DR, Shaw AT, Gadgeel S, Ahn JS, Kim D-W, et al. Alectinib versus crizotinib in untreated ALK-positive non-small-cell lung cancer. N Engl J Med 2017;377:829–38. https://doi.org/10.1056/NEJMoa1704795.

[20] Soria J-C, Tan DSW, Chiari R, Wu Y-L, Paz-Ares L, Wolf J, et al. First-line ceritinib versus platinum-based chemotherapy in advanced ALK-rearranged non-small-cell lung cancer (ASCEND-4): a randomised, open-label, phase 3 study. Lancet 2017;389:917–29. https://doi.org/10.1016/S0140-6736(17)30123-X.

[21] André F, Ciruelos E, Rubovszky G, Campone M, Loibl S, Rugo HS, et al. Alpelisib for PIK3CA-Mutated, Hormone Receptor–Positive Advanced Breast Cancer. N Engl J Med 2019;380:1929–40. https://doi.org/10.1056/NEJMoa1813904.

[22] Dagogo-Jack I, Piotrowska Z, Cobb R, Banwait M, Lennerz JK, Hata AN, et al. Response to the combination of osimertinib and trametinib in a patient with EGFR-mutant NSCLC harboring an acquired BRAF fusion. J Thorac Oncol 2019;14:e226–8. https://doi.org/10.1016/j.jtho.2019.05.046.

[23] Vojnic M, Kubota D, Kurzatkowski C, Offin M, Suzawa K, Benayed R, et al. Acquired BRAF rearrangements induce secondary resistance to EGFR therapy in EGFR-mutated lung cancers. J Thorac Oncol 2019;14:802–15. https://doi.org/10.1016/j.jtho.2018.12.038.

[24] Wang C-Y, Hsia J-Y, Li C-H, Ho C-C, Chao W-R, Wu M-F. Lung adenocarcinoma with primary LIMD1-BRAF fusion treated with MEK inhibitor: A case report. Clin Lung Cancer 2021;22:e878–80. https://doi.org/10.1016/j.cllc.2021.05.003.

[25] Kim JW, Lee K-H, Kim J-W, Suh KJ, Nam A-R, Bang J-H, et al. Enhanced antitumor effect of binimetinib in combination with capecitabine for biliary tract cancer patients with mutations in the RAS/RAF/MEK/ERK pathway: phase Ib study. Br J Cancer 2019;121:332–9. https://doi.org/10.1038/s41416-019-0523-5.

[26] Dummer R, Schadendorf D, Ascierto PA, Arance A, Dutriaux C, Di Giacomo AM, et al. Binimetinib versus dacarbazine in patients with advanced NRAS-mutant melanoma (NEMO): a multicentre, open-label, randomised, phase 3 trial. Lancet Oncol 2017;18:435–45. https://doi.org/10.1016/S1470-2045(17)30180-8.

[27] Wolf J, Seto T, Han J-Y, Reguart N, Garon EB, Groen HJM, et al. Capmatinib in MET Exon 14-mutated or MET-amplified non-small-cell lung cancer. N Engl J Med 2020;383:944–57. https://doi.org/10.1056/NEJMoa2002787.

[28] Drilon A, Clark JW, Weiss J, Ou S-HI, Camidge DR, Solomon BJ, et al. Antitumor activity of crizotinib in lung cancers harboring a MET exon 14 alteration. Nat Med 2020;26:47–51. https://doi.org/10.1038/s41591-019-0716-8.

[29] Mendenhall MA, Goldman JW. MET-mutated NSCLC with major response to crizotinib. J Thorac Oncol 2015;10:e33–4. https://doi.org/10.1097/JTO.0000000000000491.

[30] Naganuma A, Sakuda T, Murakami T, Aihara K, Watanuki Y, Suzuki Y, et al. Microsatellite instability-high intrahepatic cholangiocarcinoma with portal vein tumor thrombosis successfully treated with pembrolizumab. Intern Med 2020;59:2261–7. https://doi.org/10.2169/internalmedicine.4588-20.

[31] Lin J, Cao Y, Yang X, Li G, Shi Y, Wang D, et al. Mutational spectrum and precision oncology for biliary tract carcinoma. Theranostics 2021;11:4585–98. https://doi.org/10.7150/thno.56539.

[32] Zhu AX, Macarulla T, Javle MM, Kelley RK, Lubner SJ, Adeva J, et al. Final Overall Survival Efficacy Results of Ivosidenib for Patients With Advanced Cholangiocarcinoma With IDH1 Mutation: The Phase 3 Randomized Clinical ClarIDHy Trial. JAMA Oncol 2021;7:1669–77. https://doi.org/10.1001/jamaoncol.2021.3836.

[33] Roboz GJ, DiNardo CD, Stein EM, de Botton S, Mims AS, Prince GT, et al. Ivosidenib induces deep durable remissions in patients with newly diagnosed IDH1-mutant acute myeloid leukemia. Blood 2020;135:463–71. https://doi.org/10.1182/blood.2019002140.

[34] Graff JN, Alumkal JJ, Drake CG, Thomas G V, Redmond WL, Farhad M, et al. Early evidence of anti-PD-1 activity in enzalutamide-resistant prostate cancer. Oncotarget; Vol 7, No 33 2016.

[35] de Bono J, Mateo J, Fizazi K, Saad F, Shore N, Sandhu S, et al. Olaparib for Metastatic Castration-Resistant Prostate Cancer. N Engl J Med 2020;382:2091–102. https://doi.org/10.1056/NEJMoa1911440.

[36] Mateo J, Porta N, Bianchini D, McGovern U, Elliott T, Jones R, et al. Olaparib in patients with metastatic castration-resistant prostate cancer with DNA repair gene aberrations (TOPARP-B): a multicentre, open-label, randomised, phase 2 trial. Lancet Oncol 2020;21:162–74. https://doi.org/10.1016/S1470-2045(19)30684-9.

[37] Kornauth CF, Herbaux C, Boidol B, Guillemette C, Mayerhöfer ME, Jäger U, et al. The combination of venetoclax and ibrutinib is effective in relapsed/refractory T-prolymphocytic leukemia and influences BCL-2-family member dependencies. Hematol Oncol 2019;37:482–4. https://doi.org/https://doi.org/10.1002/hon.161_2631.

[38] Gomez-Arteaga A, Margolskee E, Wei MT, van Besien K, Inghirami G, Horwitz S. Combined use of tofacitinib (pan-JAK inhibitor) and ruxolitinib (a JAK1/2 inhibitor) for refractory T-cell prolymphocytic leukemia (T-PLL) with a JAK3 mutation. Leuk Lymphoma 2019;60:1626–31. https://doi.org/10.1080/10428194.2019.1594220.

[39] Herbaux C, Kornauth C, Poulain S, Tournilhac O, Collins MC, Valentin R, et al. Characterizing the Anti-Apoptotic Dependencies of T-Cell Prolymphocytic Leukemia Identifies HDAC and JAK/STAT Pathway Inhibitors As Promising Combination Partners to Augment Bcl-2 Targeted Killing By Venetoclax. Blood 2019;134:807. https://doi.org/10.1182/blood-2019-126773.

[40] Braun T, von Jan J, Wahnschaffe L, Herling M. Advances and Perspectives in the Treatment of T-PLL. Curr Hematol Malig Rep 2020;15:113–24. https://doi.org/10.1007/s11899-020-00566-5.

[41] Li M, Mou Y, Hou S, Cao D, Li A. Response of germline BRCA2-mutated advanced pancreatic acinar cell carcinoma to olaparib: A case report. Med 2018;97:e13113. https://doi.org/10.1097/MD.0000000000013113.

[42] Lemonnier F, Dupuis J, Sujobert P, Tournillhac O, Cheminant M, Sarkozy C, et al. Treatment with 5-azacytidine induces a sustained response in patients with angioimmunoblastic T-cell lymphoma. Blood 2018;132:2305–9. https://doi.org/10.1182/blood-2018-04-840538.

[43] Dickson MA, Tap WD, Keohan ML, D’Angelo SP, Gounder MM, Antonescu CR, et al. Phase II trial of the CDK4 inhibitor PD0332991 in patients with advanced CDK4-amplified well-differentiated or dedifferentiated liposarcoma. J Clin Oncol 2013;31:2024–8. https://doi.org/10.1200/JCO.2012.46.5476.

[44] Dickson MA, Schwartz GK, Keohan ML, D’Angelo SP, Gounder MM, Chi P, et al. Progression-free survival among patients with well-differentiated or dedifferentiated liposarcoma treated with CDK4 inhibitor palbociclib: A phase 2 clinical trial. JAMA Oncol 2016;2:937–40. https://doi.org/10.1001/jamaoncol.2016.0264.

[45] Dickson MA, Koff A, D’Angelo SP, Gounder MM, Keohan ML, Kelly CM, et al. Phase 2 study of the CDK4 inhibitor abemaciclib in dedifferentiated liposarcoma. J Clin Oncol 2019;37:11004. https://doi.org/10.1200/jco.2019.37.15_suppl.11004.

[46] Valverde CM, Martin Broto J, Lopez-Martin JA, Romagosa C, Sancho Marquez MP, Carrasco JA, et al. Phase II clinical trial evaluating the activity and tolerability of pazopanib in patients (pts) with advanced and/or metastatic liposarcoma (LPS): A joint Spanish Sarcoma Group (GEIS) and German Interdisciplinary Sarcoma Group (GISG) Study—NCT01692496. J Clin Oncol 2016;34:11039. https://doi.org/10.1200/jco.2016.34.15_suppl.11039.

[47] Qiao J, Li M, Sun D, Li W, Xin Y. Knockdown of ROS proto-oncogene 1 inhibits migration and invasion in gastric cancer cells by targeting the PI3K/Akt signaling pathway. Onco Targets Ther 2019;12:8569–82. https://doi.org/10.2147/OTT.S213421.

[48] Davidson M, Chong IY-S, Cunningham D, Aronson L, Bryant H, Begum R, et al. iMYC: Proof-of-concept study of ibrutinib in c-MYC and HER2 amplified oesophagogastric carcinoma. J Clin Oncol 2017;35:TPS221–TPS221. https://doi.org/10.1200/JCO.2017.35.4_suppl.TPS221.

[49] Wang JD, Chen XY, Ji KW, Tao F. Targeting Btk with ibrutinib inhibit gastric carcinoma cells growth. Am J Transl Res 2016;8:3003–12.

[50] Martin-Broto J, Stacchiotti S, Lopez-Pousa A, Redondo A, Bernabeu D, de Alava E, et al. Pazopanib for treatment of advanced malignant and dedifferentiated solitary fibrous tumour: a multicentre, single-arm, phase 2 trial. Lancet Oncol 2019;20:134–44. https://doi.org/10.1016/S1470-2045(18)30676-4.

[51] Stacchiotti S, Simeone N, Lo Vullo S, Morosi C, Greco FG, Gronchi A, et al. Activity of axitinib in progressive advanced solitary fibrous tumour: Results from an exploratory, investigator-driven phase 2 clinical study. Eur J Cancer 2019;106:225–33. https://doi.org/10.1016/j.ejca.2018.10.024.

[52] Liu M. Combination treatment with trastuzumab and crizotinib in metastatic gastric cancer harboring Her-2 amplification and c-MET amplification: A case report. Medicine (Baltimore) 2021;100.

[53] Shattuck DL, Miller JK, Carraway 3rd KL, Sweeney C. Met receptor contributes to trastuzumab resistance of Her2-overexpressing breast cancer cells. Cancer Res 2008;68:1471–7. https://doi.org/10.1158/0008-5472.CAN-07-5962.

[54] Smit EF, Nakagawa K, Nagasaka M, Felip E, Goto Y, Li BT, et al. Trastuzumab deruxtecan (T-DXd; DS-8201) in patients with HER2-mutated metastatic non-small cell lung cancer (NSCLC): Interim results of DESTINY-Lung01. J Clin Oncol 2020;38:9504. https://doi.org/10.1200/JCO.2020.38.15_suppl.9504.

[55] Modi S, Saura C, Yamashita T, Park YH, Kim S-B, Tamura K, et al. Trastuzumab deruxtecan in previously treated HER2-positive breast cancer. N Engl J Med 2020;382:610–21. https://doi.org/10.1056/NEJMoa1914510.

[56] Razak ARA, Ahn M-J, Yen C-J, Solomon BJ, Lee S-H, Wang H-M, et al. Phase lb/ll study of the PI3Kα inhibitor BYL719 in combination with cetuximab in recurrent/metastatic squamous cell cancer of the head and neck (SCCHN). J Clin Oncol 2014;32:6044. https://doi.org/10.1200/jco.2014.32.15_suppl.6044.

[57] Liu X, Kambrick S, Fu S, Naing A, Subbiah V, Blumenschein GR, et al. Advanced malignancies treated with a combination of the VEGF inhibitor bevacizumab, anti-EGFR antibody cetuximab, and the mTOR inhibitor temsirolimus. Oncotarget; Vol 7, No 17 2016.

[58] Garmezy B, Gheeya JS, Thein KZ, Pilie PG, Wang W, Rodon Ahnert J, et al. Correlation of pathogenic POLE mutations with clinical benefit to immune checkpoint inhibitor therapy. J Clin Oncol 2020;38:3008. https://doi.org/10.1200/jco.2020.38.15_suppl.3008.

[59] Wang F, Zhao Q, Wang Y-N, Jin Y, He M-M, Liu Z-X, et al. Evaluation of POLE and POLD1 mutations as biomarkers for immunotherapy outcomes across multiple cancer types. JAMA Oncol 2019;5:1504–6. https://doi.org/10.1001/jamaoncol.2019.2963.

[60] Ben-Ami E, Barysauskas CM, Solomon S, Tahlil K, Malley R, Hohos M, et al. Immunotherapy with single agent nivolumab for advanced leiomyosarcoma of the uterus: Results of a phase 2 study. Cancer 2017;123:3285–90. https://doi.org/10.1002/cncr.30738.

[61] Juric D, Rodon J, Tabernero J, Janku F, Burris HA, Schellens JHM, et al. Phosphatidylinositol 3-kinase a–selective inhibition with alpelisib (BYL719) in PIK3CA-altered solid tumors: Results from the first-in-human study. J Clin Oncol 2018;36:1291–9. https://doi.org/10.1200/JCO.2017.72.7107.

[62] Cho J, Kim HS, Ku BM, Choi Y-L, Cristescu R, Han J, et al. Pembrolizumab for Patients With Refractory or Relapsed Thymic Epithelial Tumor: An Open-Label Phase II Trial. J Clin Oncol 2018;37:2162–70. https://doi.org/10.1200/JCO.2017.77.3184.

[63] Giaccone G, Kim C, Thompson J, McGuire C, Kallakury B, Chahine JJ, et al. Pembrolizumab in patients with thymic carcinoma: a single-arm, single-centre, phase 2 study. Lancet Oncol 2018;19:347–55. https://doi.org/10.1016/s1470-2045(18)30062-7.

[64] Carvajal RD, Antonescu CR, Wolchok JD, Chapman PB, Roman R-A, Teitcher J, et al. KIT as a therapeutic target in metastatic melanoma. JAMA 2011;305:2327–34. https://doi.org/10.1001/jama.2011.746.

[65] Kalinsky K, Lee S, Rubin KM, Lawrence DP, Iafrarte AJ, Borger DR, et al. A phase 2 trial of dasatinib in patients with locally advanced or stage IV mucosal, acral, or vulvovaginal melanoma: A trial of the ECOG-ACRIN Cancer Research Group (E2607). Cancer 2017;123:2688–97. https://doi.org/10.1002/cncr.30663.

[66] Philip PA, Mahoney MR, Allmer C, Thomas J, Pitot HC, Kim G, et al. Phase II study of erlotinib in patients with advanced biliary cancer. J Clin Oncol 2006;24:3069–74. https://doi.org/10.1200/JCO.2005.05.3579.

[67] Maron SB, Alpert L, Kwak HA, Lomnicki S, Chase L, Xu D, et al. Targeted therapies for targeted populations: Anti-EGFR treatment for EGFR-amplified gastroesophageal adenocarcinoma. Cancer Discov 2018;8:696–713. https://doi.org/10.1158/2159-8290.cd-17-1260.

[68] Egan JB, Marks DL, Hogenson TL, Vrabel AM, Sigafoos AN, Tolosa EJ, et al. Molecular Modeling and Functional Analysis of Exome Sequencing–Derived Variants of Unknown Significance Identify a Novel, Constitutively Active FGFR2 Mutant in Cholangiocarcinoma. JCO Precis Oncol 2017:1–13. https://doi.org/10.1200/PO.17.00018.

[69] Fizazi K, Maillard A, Penel N, Baciarello G, Allouache D, Daugaard G, et al. A phase III trial of empiric chemotherapy with cisplatin and gemcitabine or systemic treatment tailored by molecular gene expression analysis in patients with carcinomas of an unknown primary (CUP) site (GEFCAPI 04). Ann Oncol 2019;30:v851. https://doi.org/10.1093/annonc/mdz394.

[70] Abou-Alfa GK, Sahai V, Hollebecque A, Vaccaro G, Melisi D, Al-Rajabi R, et al. Pemigatinib for previously treated, locally advanced or metastatic cholangiocarcinoma: a multicentre, open-label, phase 2 study. Lancet Oncol 2020;21:671–84. https://doi.org/10.1016/S1470-2045(20)30109-1.

[71] Lin J, Shi W, Zhao S, Hu J, Hou Z, Yao M, et al. Lenvatinib plus checkpoint inhibitors in patients (pts) with advanced intrahepatic cholangiocarcinoma (ICC): Preliminary data and correlation with next-generation sequencing. J Clin Oncol 2018;36:500. https://doi.org/10.1200/jco.2018.36.4_suppl.500.

[72] Bowyer SE, Rao AD, Lyle M, Sandhu S, Long G V, McArthur GA, et al. Activity of trametinib in K601E and L597Q BRAF mutation-positive metastatic melanoma. Melanoma Res 2014;24:504–8. https://doi.org/10.1097/CMR.0000000000000099.

[73] Marconcini R, Galli L, Antonuzzo A, Bursi S, Roncella C, Fontanini G, et al. Metastatic BRAF K601E-mutated melanoma reaches complete response to MEK inhibitor trametinib administered for over 36 months. Exp Hematol Oncol 2017;6:6. https://doi.org/10.1186/s40164-017-0067-4.

[74] Kim KB, Kefford R, Pavlick AC, Infante JR, Ribas A, Sosman JA, et al. Phase II study of the MEK1/MEK2 inhibitor Trametinib in patients with metastatic BRAF-mutant cutaneous melanoma previously treated with or without a BRAF inhibitor. J Clin Oncol 2013;31:482–9. https://doi.org/10.1200/JCO.2012.43.5966.

[75] Marabelle A, Le DT, Ascierto PA, Di Giacomo AM, De Jesus-Acosta A, Delord J-P, et al. Efficacy of pembrolizumab in patients with noncolorectal high microsatellite instability/mismatch repair–deficient cancer: Results from the phase II KEYNOTE-158 study. J Clin Oncol 2020;38:1–10. https://doi.org/10.1200/jco.19.02105.

[76] Le DT, Uram JN, Wang H, Bartlett BR, Kemberling H, Eyring AD, et al. PD-1 blockade in tumors with mismatch-repair deficiency. N Engl J Med 2015;372:2509–20. https://doi.org/10.1056/NEJMoa1500596.

[77] Ho AS, Ochoa A, Jayakumaran G, Zehir A, Valero Mayor C, Tepe J, et al. Genetic hallmarks of recurrent/metastatic adenoid cystic carcinoma. J Clin Invest 2019;129:4276–89. https://doi.org/10.1172/JCI128227.

[78] Kaufman B, Shapira-Frommer R, Schmutzler RK, Audeh MW, Friedlander M, Balmaña J, et al. Olaparib monotherapy in patients with advanced cancer and a germline BRCA1/2 mutation. J Clin Oncol 2015;33:244–50. https://doi.org/10.1200/JCO.2014.56.2728.

[79] Maroto P, Anguera G, Roldan-Romero JM, Apellániz-Ruiz M, Algaba F, Boonman J, et al. Biallelic TSC2 Mutations in a Patient With Chromophobe Renal Cell Carcinoma Showing Extraordinary Response to Temsirolimus. J Natl Compr Canc Netw 2018;16:352–8. https://doi.org/10.6004/jnccn.2017.7041.

[80] Volkov NM, Yanus GA, Ivantsov AO, Moiseenko F V, Matorina OG, Bizin I V, et al. Efficacy of immune checkpoint blockade in MUTYH-associated hereditary colorectal cancer. Invest New Drugs 2020;38:894–8. https://doi.org/10.1007/s10637-019-00842-z.

[81] Ahn S, Lee J, Shin DW, Kim J, Hwang J-H. High PD-L1 expression is associated with therapeutic response to pembrolizumab in patients with advanced biliary tract cancer. Sci Rep 2020;10:12348. https://doi.org/10.1038/s41598-020-69366-4.

[82] Bang Y-J, Ueno M, Malka D, Chung HC, Nagrial A, Kelley RK, et al. Pembrolizumab (pembro) for advanced biliary adenocarcinoma: Results from the KEYNOTE-028 (KN028) and KEYNOTE-158 (KN158) basket studies. J Clin Oncol 2019;37:4079. https://doi.org/10.1200/JCO.2019.37.15_suppl.4079.

[83] Fabrizio DA, George Jr TJ, Dunne RF, Frampton G, Sun J, Gowen K, et al. Beyond microsatellite testing: assessment of tumor mutational burden identifies subsets of colorectal cancer who may respond to immune checkpoint inhibition. J Gastrointest Oncol 2018;9:610–7. https://doi.org/10.21037/jgo.2018.05.06.

[84] Hellmann MD, Ciuleanu T-E, Pluzanski A, Lee JS, Otterson GA, Audigier-Valette C, et al. Nivolumab plus ipilimumab in lung cancer with a high tumor mutational burden. N Engl J Med 2018;378:2093–104. https://doi.org/10.1056/NEJMoa1801946.

[85] Marabelle A, Fakih M, Lopez J, Shah M, Shapira-Frommer R, Nakagawa K, et al. Association of tumour mutational burden with outcomes in patients with advanced solid tumours treated with pembrolizumab: prospective biomarker analysis of the multicohort, open-label, phase 2 KEYNOTE-158 study. Lancet Oncol 2020;21:1353–65. https://doi.org/10.1016/S1470-2045(20)30445-9.

[86] Gbyli R, Song Y, Liu W, Gao Y, Biancon G, Chandhok NS, et al. In vivo anti-tumor effect of PARP inhibition in IDH1/2 mutant MDS/AML resistant to targeted inhibitors of mutant IDH1/2. Leukemia 2022;36:1313–23. https://doi.org/10.1038/s41375-022-01536-x.

[87] Zhu Y-C, Wang W-X, Xu C-W, Song Z-B, Du K-Q, Chen G, et al. EGFR-RAD51 fusion variant in lung adenocarcinoma and response to erlotinib: A case report. Lung Cancer 2018;115:131–4. https://doi.org/10.1016/j.lungcan.2017.12.001.

[88] Konduri K, Gallant J-N, Chae YK, Giles FJ, Gitlitz BJ, Gowen K, et al. EGFR fusions as novel therapeutic targets in lung cancer. Cancer Discov 2016;6:601–11. https://doi.org/10.1158/2159-8290.CD-16-0075.

[89] Palma NA, Ali SM, O’Connor J, Dutta D, Wang K, Soman S, et al. Durable response to crizotinib in a MET-amplified, KRAS-mutated carcinoma of unknown primary. Case Rep Oncol 2014;7:503–8. https://doi.org/10.1159/000365326.

[90] Li BT, Michelini F, Misale S, Cocco E, Baldino L, Cai Y, et al. HER2-mediated internalization of cytotoxic agents in ERBB2 amplified or mutant lung cancers. Cancer Discov 2020;10:674–87. https://doi.org/10.1158/2159-8290.CD-20-0215.

[91] Janjigian YY, Shitara K, Moehler M, Garrido M, Salman P, Shen L, et al. First-line nivolumab plus chemotherapy versus chemotherapy alone for advanced gastric, gastro-oesophageal junction, and oesophageal adenocarcinoma (CheckMate 649): a randomised, open-label, phase 3 trial. Lancet 2021;398:27–40. https://doi.org/10.1016/S0140-6736(21)00797-2.

[92] Wang F, Wei XL, Wang FH, Xu N, Shen L, Dai GH, et al. Safety, efficacy and tumor mutational burden as a biomarker of overall survival benefit in chemo-refractory gastric cancer treated with toripalimab, a PD-1 antibody in phase Ib/II clinical trial NCT02915432. Ann Oncol 2019;30:1479–86. https://doi.org/10.1093/annonc/mdz197.

[93] Lennerz JK, Kwak EL, Ackerman A, Michael M, Fox SB, Bergethon K, et al. MET amplification identifies a small and aggressive subgroup of esophagogastric adenocarcinoma with evidence of responsiveness to crizotinib. J Clin Oncol Off J Am Soc Clin Oncol 2011;29:4803–10. https://doi.org/10.1200/JCO.2011.35.4928.

[94] Hou GX, Song B Bin. Gastric cancer patient with c-MET amplification treated with crizotinib after failed multi-line treatment: A case report and literature review. Math Biosci Eng 2019;16:5923–30. https://doi.org/10.3934/mbe.2019296.

[95] Lau DK, Tay RY, Yeung YH, Chionh F, Mooi J, Murone C, et al. Phase II study of everolimus (RAD001) monotherapy as first-line treatment in advanced biliary tract cancer with biomarker exploration: the RADiChol Study. Br J Cancer 2018;118:966–71. https://doi.org/10.1038/s41416-018-0021-1.

[96] Wagle N, Grabiner BC, Van Allen EM, Hodis E, Jacobus S, Supko JG, et al. Activating mTOR mutations in a patient with an extraordinary response on a phase I trial of everolimus and pazopanib. Cancer Discov 2014;4:546–53. https://doi.org/10.1158/2159-8290.CD-13-0353.

[97] Rodríguez-Moreno JF, Apellaniz-Ruiz M, Roldan-Romero JM, Durán I, Beltrán L, Montero-Conde C, et al. Exceptional response to temsirolimus in a metastatic clear cell renal cell carcinoma with an early novel MTOR -activating mutation. J Natl Compr Canc Netw 2017;15:1310–5. https://doi.org/10.6004/jnccn.2017.7018.

[98] Coleman N, Subbiah V, Pant S, Patel K, Roy-Chowdhuri S, Yedururi S, et al. Emergence of mTOR mutation as an acquired resistance mechanism to AKT inhibition, and subsequent response to mTORC1/2 inhibition. NPJ Precis Oncol 2021;5:99. https://doi.org/10.1038/s41698-021-00240-w.

[99] Finn RS, Qin S, Ikeda M, Galle PR, Ducreux M, Kim T-Y, et al. IMbrave150: Updated overall survival (OS) data from a global, randomized, open-label phase III study of atezolizumab (atezo) + bevacizumab (bev) versus sorafenib (sor) in patients (pts) with unresectable hepatocellular carcinoma (HCC). J Clin Oncol 2021;39:267. https://doi.org/10.1200/jco.2021.39.3_suppl.267.

[100] Fushimi C, Tada Y, Takahashi H, Nagao T, Ojiri H, Masubuchi T, et al. A prospective phase II study of combined androgen blockade in patients with androgen receptor-positive metastatic or locally advanced unresectable salivary gland carcinoma. Ann Oncol 2018;29:979–84. https://doi.org/10.1093/annonc/mdx771.

[101] Modi S, Park H, Murthy RK, Iwata H, Tamura K, Tsurutani J, et al. Antitumor activity and safety of trastuzumab deruxtecan in patients with HER2-low-expressing advanced breast cancer: Results from a phase Ib study. J Clin Oncol 2020;38:1887–96. https://doi.org/10.1200/JCO.19.02318.

[102] Bando H, Kinoshita I, Modi S, Tsurutani J, Bang Y-J, Iwata H, et al. Trastuzumab deruxtecan (T-DXd) in patients with human epidermal growth factor receptor 2 (HER2)-expressing salivary duct carcinoma: Subgroup analysis of two phase 1 studies. J Clin Oncol 2021;39:6079. https://doi.org/10.1200/jco.2021.39.15_suppl.6079.

[103] Zhang W, Shi J, Li R, Han Z, Li L, Li G, et al. Effectiveness of olaparib treatment in a patient with gallbladder cancer with an ATM-inactivating mutation. Oncologist 2020;25:375–9. https://doi.org/10.1634/theoncologist.2019-0498.

[104] Hussain M, Mateo J, Fizazi K, Saad F, Shore N, Sandhu S, et al. Survival with olaparib in metastatic castration-resistant prostate cancer. N Engl J Med 2020;383:2345–57. https://doi.org/10.1056/NEJMoa2022485.

[105] Yu Y, Liu Q, Li W, Qu Y, Zhang Y, Liu T. Identification of a Novel EHBP1‐MET Fusion in an Intrahepatic Cholangiocarcinoma Responding to Crizotinib. Oncologist 2020;25:1005–8. https://doi.org/10.1634/theoncologist.2020-0535.

[106] Marchand A, Tallet A, Collin C, Cormier B, Venel Y, Miquelestorena-Standley E, et al. A rare BRAF T599dup mutation conferring sensitivity to BRAF inhibitor in a patient with metastatic melanoma. Br J Dermatol 2018;179:528–9. https://doi.org/10.1111/bjd.16499.

[107] Subbiah V, Lassen U, Élez E, Italiano A, Curigliano G, Javle M, et al. Dabrafenib plus trametinib in patients with BRAFV600E-mutated biliary tract cancer (ROAR): a phase 2, open-label, single-arm, multicentre basket trial. Lancet Oncol 2020;21:1234–43. https://doi.org/10.1016/S1470-2045(20)30321-1.

[108] Kato S, Adashek JJ, Shaya J, Okamura R, Jimenez RE, Lee S, et al. Concomitant MEK and cyclin gene alterations: Implications for response to targeted therapeutics. Clin Cancer Res 2021;27:2792–7. https://doi.org/10.1158/1078-0432.CCR-20-3761.

[109] Knudsen ES, Kumarasamy V, Chung S, Ruiz A, Vail P, Tzetzo S, et al. Targeting dual signalling pathways in concert with immune checkpoints for the treatment of pancreatic cancer. Gut 2021;70:127–38. https://doi.org/10.1136/gutjnl-2020-321000.

[110] Bian L, Meng Y, Zhang M, Li D. MRE11-RAD50-NBS1 complex alterations and DNA damage response: implications for cancer treatment. Mol Cancer 2019;18:169. https://doi.org/10.1186/s12943-019-1100-5.

[111] Altan B, Yokobori T, Ide M, Bai T, Yanoma T, Kimura A, et al. High expression of MRE11-RAD50-NBS1 is associated with poor prognosis and chemoresistance in gastric cancer. Anticancer Res 2016;36:5237–47. https://doi.org/10.21873/anticanres.11094.

[112] Araki K, Yamashita T, Reddy N, Wang H, Abuzeid WM, Khan K, et al. Molecular disruption of NBS1 with targeted gene delivery enhances chemosensitisation in head and neck cancer. Br J Cancer 2010;103:1822–30. https://doi.org/10.1038/sj.bjc.6605980.

[113] Sharma P, Abramson VG, O’Dea A, Pathak HB, Pessetto ZY, Wang YY, et al. Clinical and biomarker results from phase I/II study of PI3K inhibitor BYL 719 (alpelisib) plus nab-paclitaxel in HER2-negative metastatic breast cancer. J Clin Oncol 2018;36:1018. https://doi.org/10.1200/jco.2018.36.15_suppl.1018.

[114] Dent R, Oliveira M, Isakoff SJ, Im S-A, Espié M, Blau S, et al. 139O Final results of the double-blind placebo (PBO)-controlled randomised phase II LOTUS trial of first-line ipatasertib (IPAT) + paclitaxel (PAC) for inoperable locally advanced/metastatic triple-negative breast cancer (mTNBC). Ann Oncol 2020;31:S64–5. https://doi.org/10.1016/j.annonc.2020.03.240.

[115] Yam C, Xu X, Davies MA, Gimotty PA, Morrissette JJD, Tetzlaff MT, et al. A multicenter phase I study evaluating dual PI3K and BRAF inhibition with PX-866 and vemurafenib in patients with advanced BRAF V600–mutant solid tumors. Clin Cancer Res 2018;24:22–32. https://doi.org/10.1158/1078-0432.ccr-17-1807.

[116] Bryce AH, Kurzrock R, Meric-Bernstam F, Hurwitz H, Hainsworth JD, Spigel DR, et al. Pertuzumab plus trastuzumab for HER2-positive metastatic urothelial cancer (mUC): Preliminary data from MyPathway. J Clin Oncol 2017;35:348. https://doi.org/10.1200/jco.2017.35.6_suppl.348.

[117] Wülfing C, Machiels J-PH, Richel DJ, Grimm M-O, Treiber U, De Groot MR, et al. A single-arm, multicenter, open-label phase 2 study of lapatinib as the second-line treatment of patients with locally advanced or metastatic transitional cell carcinoma. Cancer 2009;115:2881–90. https://doi.org/10.1002/cncr.24337.

[118] Galsky MD, Von Hoff DD, Neubauer M, Anderson T, Fleming M, Nagarwala Y, et al. Target-specific, histology-independent, randomized discontinuation study of lapatinib in patients with HER2-amplified solid tumors. Invest New Drugs 2012;30:695–701. https://doi.org/10.1007/s10637-010-9541-0.

[119] Gupta S, Albertson DJ, Parnell T, Dalley B, O Shea J, Weston A, et al. Clinical and translational investigation of pan-HDAC inhibition in advanced urothelial carcinoma (UC). J Clin Oncol 2017;35:379. https://doi.org/10.1200/jco.2017.35.6_suppl.379.

[120] Shitara K, Van Cutsem E, Bang Y-J, Fuchs C, Wyrwicz L, Lee K-W, et al. Efficacy and safety of pembrolizumab or pembrolizumab plus chemotherapy vs chemotherapy alone for patients with first-line, advanced gastric cancer: The KEYNOTE-062 phase 3 randomized clinical trial. JAMA Oncol 2020;6:1571–80. https://doi.org/10.1001/jamaoncol.2020.3370.

[121] Kawazoe A, Fukuoka S, Nakamura Y, Kuboki Y, Wakabayashi M, Nomura S, et al. Lenvatinib plus pembrolizumab in patients with advanced gastric cancer in the first-line or second-line setting (EPOC1706): an open-label, single-arm, phase 2 trial. Lancet Oncol 2020;21:1057–65. https://doi.org/10.1016/S1470-2045(20)30271-0.

[122] Dunn LA, Riaz N, Fury MG, McBride SM, Michel L, Lee NY, et al. A phase 1b study of cetuximab and BYL719 (alpelisib) concurrent with intensity modulated radiation therapy in stage III-IVB head and neck squamous cell carcinoma. Int J Radiat Oncol Biol Phys 2020;106:564–70. https://doi.org/10.1016/j.ijrobp.2019.09.050.

[123] Swisher EM, Lin KK, Oza AM, Scott CL, Giordano H, Sun J, et al. Rucaparib in relapsed, platinum-sensitive high-grade ovarian carcinoma (ARIEL2 Part 1): an international, multicentre, open-label, phase 2 trial. Lancet Oncol 2017;18:75–87. https://doi.org/10.1016/S1470-2045(16)30559-9.

[124] Kondrashova O, Nguyen M, Shield-Artin K, Tinker A V, Teng NNH, Harrell MI, et al. Secondary somatic mutations restoring RAD51C and RAD51D associated with acquired resistance to the PARP inhibitor rucaparib in High-grade ovarian carcinoma. Cancer Discov 2017;7:984–98. https://doi.org/10.1158/2159-8290.cd-17-0419.

[125] Bitzer M, Spahn S, Babaei S, Horger M, Singer S, Schulze-Osthoff K, et al. Targeting extracellular and juxtamembrane FGFR2 mutations in chemotherapy-refractory cholangiocarcinoma. NPJ Precis Oncol 2021;5:80. https://doi.org/10.1038/s41698-021-00220-0.

[126] Park JO, Feng Y-H, Chen Y-Y, Su W-C, Oh D-Y, Shen L, et al. Updated results of a phase IIa study to evaluate the clinical efficacy and safety of erdafitinib in Asian advanced cholangiocarcinoma (CCA) patients with FGFR alterations. J Clin Oncol 2019;37:4117. https://doi.org/10.1200/JCO.2019.37.15_suppl.4117.

[127] Javle M, Lowery M, Shroff RT, Weiss KH, Springfeld C, Borad MJ, et al. Phase II study of BGJ398 in patients with FGFR-altered advanced cholangiocarcinoma. J Clin Oncol 2018;36:276–82. https://doi.org/10.1200/JCO.2017.75.5009.

[128] L. SP, D. CC, D. RN, E. SS, R. PK, Hanwen B, et al. 2-Hydroxyglutarate produced by neomorphic IDH mutations suppresses homologous recombination and induces PARP inhibitor sensitivity. Sci Transl Med 2017;9:eaal2463. https://doi.org/10.1126/scitranslmed.aal2463.

[129] Kato K, Cho BC, Takahashi M, Okada M, Lin C-Y, Chin K, et al. Nivolumab versus chemotherapy in patients with advanced oesophageal squamous cell carcinoma refractory or intolerant to previous chemotherapy (ATTRACTION-3): a multicentre, randomised, open-label, phase 3 trial. Lancet Oncol 2019;20:1506–17. https://doi.org/10.1016/S1470-2045(19)30626-6.

[130] Siena S, Di Bartolomeo M, Raghav K, Masuishi T, Loupakis F, Kawakami H, et al. Trastuzumab deruxtecan (DS-8201) in patients with HER2-expressing metastatic colorectal cancer (DESTINY-CRC01): a multicentre, open-label, phase 2 trial. Lancet Oncol 2021;22:779–89. https://doi.org/10.1016/S1470-2045(21)00086-3.

[131] Sartore-Bianchi A, Trusolino L, Martino C, Bencardino K, Lonardi S, Bergamo F, et al. Dual-targeted therapy with trastuzumab and lapatinib in treatment-refractory, KRAS codon 12/13 wild-type, HER2-positive metastatic colorectal cancer (HERACLES): a proof-of-concept, multicentre, open-label, phase 2 trial. Lancet Oncol 2016;17:738–46. https://doi.org/10.1016/S1470-2045(16)00150-9.

[132] Meric-Bernstam F, Hurwitz H, Raghav KPS, McWilliams RR, Fakih M, VanderWalde A, et al. Pertuzumab plus trastuzumab for HER2-amplified metastatic colorectal cancer (MyPathway): an updated report from a multicentre, open-label, phase 2a, multiple basket study. Lancet Oncol 2019;20:518–30. https://doi.org/10.1016/S1470-2045(18)30904-5.

[133] Corrêa TS, Matos GDR, Segura M, Dos Anjos CH. Second-line treatment of HER2-positive salivary gland tumor: Ado-trastuzumab emtansine (T-DM1) after progression on trastuzumab. Case Rep Oncol 2018;11:252–7. https://doi.org/10.1159/000488669.

[134] Javle MM, Hainsworth JD, Swanton C, Burris HA, Kurzrock R, Sweeney C, et al. Pertuzumab + trastuzumab for HER2-positive metastatic biliary cancer: Preliminary data from MyPathway. J Clin Oncol 2017;35:402. https://doi.org/10.1200/JCO.2017.35.4_suppl.402.

[135] Meric-Bernstam F, Hanna DL, El-Khoueiry AB, Kang Y-K, Oh D-Y, Chaves JM, et al. Zanidatamab (ZW25) in HER2-positive biliary tract cancers (BTCs): Results from a phase I study. J Clin Oncol 2021;39:299. https://doi.org/10.1200/JCO.2021.39.3_suppl.299.

[136] Nam A-R, Kim J-W, Cha Y, Ha H, Park JE, Bang J-H, et al. Therapeutic implication of HER2 in advanced biliary tract cancer. Oncotarget 2016;7:58007–21. https://doi.org/10.18632/oncotarget.11157.

[137] Siena S, Di Bartolomeo M, Raghav K, Masuishi T, Loupakis F, Kawakami H, et al. Trastuzumab deruxtecan (DS-8201) in patients with HER2-expressing metastatic colorectal cancer (DESTINY-CRC01): a multicentre, open-label, phase 2 trial. Lancet Oncol 2021;22:779–89. https://doi.org/10.1016/S1470-2045(21)00086-3.

[138] Sandhu J, Wang C, Fakih M. Clinical response to T-DM1 in HER2-amplified, KRAS-mutated metastatic colorectal cancer. J Natl Compr Canc Netw 2020;18:116–9. https://doi.org/10.6004/jnccn.2019.7371.

[139] Verlingue L, Hollebecque A, Lacroix L, Postel-Vinay S, Varga A, El Dakdouki Y, et al. Human epidermal receptor family inhibitors in patients with ERBB3 mutated cancers: Entering the back door. Eur J Cancer 2018;92:1–10. https://doi.org/10.1016/j.ejca.2017.12.020.

[140] Bidard F-C, Ng CKY, Cottu P, Piscuoglio S, Escalup L, Sakr RA, et al. Response to dual HER2 blockade in a patient with HER3-mutant metastatic breast cancer. Ann Oncol 2015;26:1704–9. https://doi.org/10.1093/annonc/mdv217.

[141] Cheng Y, Zhang J, Qin SK, Hua HQ. Treatment with olaparib monotherapy for BRCA2-mutated refractory intrahepatic cholangiocarcinoma: a case report. Onco Targets Ther 2018;11:5957–62. https://doi.org/10.2147/OTT.S176914.

[142] Xiong F, Gong J, Wang Q. Olaparib and Pembrolizumab Treatment for BRCA1-Mutated and PD-L1-Positive Intrahepatic Cholangiocarcinoma Recurrence and Metastasis: A Case Report. Onco Targets Ther 2020;13:6385–91. https://doi.org/10.2147/OTT.S250454.

[143] Brose MS, Cabanillas ME, Cohen EEW, Wirth LJ, Riehl T, Yue H, et al. Vemurafenib in patients with BRAF(V600E)-positive metastatic or unresectable papillary thyroid cancer refractory to radioactive iodine: a non-randomised, multicentre, open-label, phase 2 trial. Lancet Oncol 2016;17:1272–82. https://doi.org/10.1016/S1470-2045(16)30166-8.

[144] Shah MH, Wei L, Wirth LJ, Daniels GA, De Souza JA, Timmers CD, et al. Results of randomized phase II trial of dabrafenib versus dabrafenib plus trametinib in BRAF-mutated papillary thyroid carcinoma. J Clin Oncol 2017;35:6022. https://doi.org/10.1200/JCO.2017.35.15_suppl.6022.

[145] Falchook GS, Millward M, Hong D, Naing A, Piha-Paul S, Waguespack SG, et al. BRAF inhibitor dabrafenib in patients with MetastaticBRAF-mutant thyroid cancer. Thyroid 2015;25:71–7. https://doi.org/10.1089/thy.2014.0123.

[146] Subbiah V, Kreitman RJ, Wainberg ZA, Cho JY, Schellens JHM, Soria JC, et al. Dabrafenib and trametinib treatment in patients with locally advanced or metastatic BRAF V600–mutant anaplastic thyroid cancer. J Clin Oncol 2018;36:7–13. https://doi.org/10.1200/jco.2017.73.6785.

[147] Rothenberg SM, McFadden DG, Palmer EL, Daniels GH, Wirth LJ. Redifferentiation of iodine-refractory BRAF V600E-mutant metastatic papillary thyroid cancer with dabrafenib. Clin Cancer Res 2015;21:1028–35. https://doi.org/10.1158/1078-0432.CCR-14-2915.

[148] Dunn LA, Sherman EJ, Baxi SS, Tchekmedyian V, Grewal RK, Larson SM, et al. Vemurafenib redifferentiation of BRAF mutant, RAI-refractory thyroid cancers. J Clin Endocrinol Metab 2019;104:1417–28. https://doi.org/10.1210/jc.2018-01478.

[149] Menzer C, Menzies AM, Carlino MS, Reijers I, Groen EJ, Eigentler T, et al. Targeted therapy in advanced melanoma with rare BRAF mutations. J Clin Oncol 2019;37:3142–51. https://doi.org/10.1200/JCO.19.00489.

[150] Richtig G, Aigelsreiter A, Kashofer K, Talakic E, Kupsa R, Schaider H, et al. Two case reports of rare BRAF mutations in Exon 11 and Exon 15 with discussion of potential treatment options. Case Rep Oncol 2016;9:543–6. https://doi.org/10.1159/000449125.

[151] Wang J, Li X, Xue X, Ou Q, Wu X, Liang Y, et al. Clinical outcomes of EGFR kinase domain duplication to targeted therapies in NSCLC. Int J Cancer 2019;144:2677–82. https://doi.org/10.1002/ijc.31895.

[152] Gallant J-N, Sheehan JH, Shaver TM, Bailey M, Lipson D, Chandramohan R, et al. EGFR kinase domain duplication (EGFR-KDD) is a novel oncogenic driver in lung cancer that is clinically responsive to afatinib. Cancer Discov 2015;5:1155–63. https://doi.org/10.1158/2159-8290.CD-15-0654.

[153] Zhang L-D, Gao H, Qin S-M, Zeng Q, Chen Q-F. Osimertinib is an effective epidermal growth factor receptor-tyrosine kinase inhibitor choice for lung cancer with epidermal growth factor receptor exon 18-25 kinase domain duplication: report of two cases. Anticancer Drugs 2022;33:e486–90. https://doi.org/10.1097/CAD.0000000000001148.

[154] Hirokawa E, Watanabe S, Sakai K, Takeda M, Sato C, Takahama T, et al. Durable response to EGFR tyrosine kinase inhibitors in a patient with non-small cell lung cancer harboring an EGFR kinase domain duplication. Thorac Cancer 2021;12:2283–7. https://doi.org/10.1111/1759-7714.14081.

[155] Loriot Y, Schuler MH, Iyer G, Witt O, Doi T, Qin S, et al. Tumor agnostic efficacy and safety of erdafitinib in patients (pts) with advanced solid tumors with prespecified fibroblast growth factor receptor alterations (FGFRalt) in RAGNAR: Interim analysis (IA) results. J Clin Oncol 2022;40:3007. https://doi.org/10.1200/JCO.2022.40.16_suppl.3007.

[156] Loriot Y, Necchi A, Park SH, Garcia-Donas J, Huddart R, Burgess E, et al. Erdafitinib in Locally Advanced or Metastatic Urothelial Carcinoma. N Engl J Med 2019. https://doi.org/10.1056/nejmoa1817323.

[157] Burtness B, Harrington KJ, Greil R, Soulières D, Tahara M, de Castro Jr G, et al. Pembrolizumab alone or with chemotherapy versus cetuximab with chemotherapy for recurrent or metastatic squamous cell carcinoma of the head and neck (KEYNOTE-048): a randomised, open-label, phase 3 study. Lancet 2019;394:1915–28. https://doi.org/10.1016/S0140-6736(19)32591-7.

[158] Moskowitz AJ, Ghione P, Jacobsen ED, Ruan J, Schatz JH, Noor S, et al. Final Results of a Phase II Biomarker-Driven Study of Ruxolitinib in Relapsed and Refractory T-Cell Lymphoma. Blood 2019;134:4019. https://doi.org/10.1182/blood-2019-125017.

[159] Pham HTT, Hengstschläger M, Moriggl R. A haunted beast: Targeting STAT5BN642H in T-Cell Neoplasia. Mol Cell Oncol 2018;5:e1435181. https://doi.org/10.1080/23723556.2018.1435181.

[160] Hainsworth JD, Meric-Bernstam F, Swanton C, Hurwitz H, Spigel DR, Sweeney C, et al. Targeted therapy for advanced solid tumors on the basis of molecular profiles: Results from MyPathway, an open-label, phase IIa multiple basket study. J Clin Oncol 2018;36:536–42. https://doi.org/10.1200/jco.2017.75.3780.

[161] Javle M, Churi C, Kang HC, Shroff R, Janku F, Surapaneni R, et al. HER2/neu-directed therapy for biliary tract cancer. J Hematol Oncol 2015;8:58. https://doi.org/10.1186/s13045-015-0155-z.

[162] Kato S, Okamura R, Mareboina M, Lee S, Goodman A, Patel SP, et al. Revisiting Epidermal Growth Factor Receptor (EGFR) Amplification as a Target for Anti-EGFR Therapy: Analysis of Cell-Free Circulating Tumor DNA in Patients With Advanced Malignancies. JCO Precis Oncol 2019:1–14. https://doi.org/10.1200/PO.18.00180.

[163] Díaz-Serrano A, Sánchez-Torre A, Paz-Ares L. Necitumumab for the treatment of advanced non-small-cell lung cancer. Future Oncol 2019;15:705–16. https://doi.org/10.2217/fon-2018-0594.

[164] Wang X, Niu H, Fan Q, Lu P, Ma C, Liu W, et al. Predictive value of EGFR overexpression and gene amplification on icotinib efficacy in patients with advanced esophageal squamous cell carcinoma. Oncotarget 2016;7:24744–51. https://doi.org/10.18632/oncotarget.8271.

[165] Li XS, Li XF, Jin LF, Zhao Y, Shen WZ. Treatment of ALK negative metastatic inflammatory myofibroblastic tumor by combination of pembrolizumab and anlotinib: a case report. Zhonghua Zhong Liu Za Zhi 2021;43:240–2. https://doi.org/10.3760/cma.j.cn112152-20191216-00810.

[166] Ghiringhelli F, Richard C, Chevrier S, Végran F, Boidot R. Efficiency of olaparib in colorectal cancer patients with an alteration of the homologous repair protein. World J Gastroenterol 2016;22:10680–6. https://doi.org/10.3748/wjg.v22.i48.10680.

[167] Mellinghoff IK, Ellingson BM, Touat M, Maher E, De La Fuente MI, Holdhoff M, et al. Ivosidenib in Isocitrate Dehydrogenase 1-Mutated Advanced Glioma. J Clin Oncol Off J Am Soc Clin Oncol 2020;38:3398–406. https://doi.org/10.1200/JCO.19.03327.

[168] Demetri GD, Reichardt P, Kang Y-K, Blay J-Y, Rutkowski P, Gelderblom H, et al. Efficacy and safety of regorafenib for advanced gastrointestinal stromal tumours after failure of imatinib and sunitinib (GRID): an international, multicentre, randomised, placebo-controlled, phase 3 trial. Lancet (London, England) 2013;381:295–302. https://doi.org/10.1016/S0140-6736(12)61857-1.

[169] Mir O, Cropet C, Toulmonde M, Cesne A Le, Molimard M, Bompas E, et al. Pazopanib plus best supportive care versus best supportive care alone in advanced gastrointestinal stromal tumours resistant to imatinib and sunitinib (PAZOGIST): a randomised, multicentre, open-label phase 2 trial. Lancet Oncol 2016;17:632–41. https://doi.org/10.1016/s1470-2045(16)00075-9.

[170] Park SH, Ryu MH, Ryoo BY, Im SA, Kwon HC, Lee SS, et al. Sorafenib in patients with metastatic gastrointestinal stromal tumors who failed two or more prior tyrosine kinase inhibitors: a phase II study of Korean gastrointestinal stromal tumors study group. Invest New Drugs 2012;30:2377–83. https://doi.org/10.1007/s10637-012-9795-9.

[171] Reichardt P, Blay J-Y, Gelderblom H, Schlemmer M, Demetri GD, Bui-Nguyen B, et al. Phase III study of nilotinib versus best supportive care with or without a TKI in patients with gastrointestinal stromal tumors resistant to or intolerant of imatinib and sunitinib. Ann Oncol Off J Eur Soc Med Oncol 2012;23:1680–7. https://doi.org/10.1093/annonc/mdr598.

[172] Zhou H, Shen J, Liu J, Fang W, Zhang L. Efficacy of Immune Checkpoint Inhibitors in SMARCA4-Mutant NSCLC. J Thorac Oncol 2020;15:e133–6. https://doi.org/10.1016/j.jtho.2020.03.030.

[173] Schoenfeld AJ, Bandlamudi C, Lavery JA, Montecalvo J, Namakydoust A, Rizvi H, et al. The Genomic Landscape of SMARCA4 Alterations and Associations with Outcomes in Patients with Lung Cancer. Clin Cancer Res an Off J Am Assoc Cancer Res 2020;26:5701–8. https://doi.org/10.1158/1078-0432.CCR-20-1825.

[174] Plenker D, Bertrand M, de Langen AJ, Riedel R, Lorenz C, Scheel AH, et al. Structural Alterations of MET Trigger Response to MET Kinase Inhibition in Lung Adenocarcinoma Patients. Clin Cancer Res an Off J Am Assoc Cancer Res 2018;24:1337–43. https://doi.org/10.1158/1078-0432.CCR-17-3001.

[175] Wang WX, Xu C, Chen Y, Zhu Y-C, Liu Y, Wang H, et al. MET gene fusions in non-small cell lung cancer (NSCLC) in the Chinese population: A multicenter study. J Clin Oncol 2018;36:e13539–e13539. https://doi.org/10.1200/jco.2018.36.15_suppl.e13539.

[176] Davies KD, Ng TL, Estrada-Bernal A, Le AT, Ennever PR, Camidge DR, et al. Dramatic Response to Crizotinib in a Patient With Lung Cancer Positive for an HLA-DRB1-MET Gene Fusion. JCO Precis Oncol 2017:1–6. https://doi.org/10.1200/PO.17.00117.

[177] Scheiter A, Keil F, Lüke F, Grosse J, Verloh N, Opitz S, et al. Identification and In-Depth Analysis of the Novel FGFR2-NDC80 Fusion in a Cholangiocarcinoma Patient : Implication for Therapy 2021:1161–9.

[178] Naito T, Umemura S, Nakamura H, Zenke Y, Udagawa H, Kirita K, et al. Successful treatment with nivolumab for SMARCA4-deficient non-small cell lung carcinoma with a high tumor mutation burden: A case report. Thorac Cancer 2019;10:1285–8. https://doi.org/10.1111/1759-7714.13070.

[179] Shah MH, Sherman EJ, Robinson B, Solomon BJ, Kang H, Lorch JH, et al. Selpercatinib (LOXO-292) in patients with RET-mutant medullary thyroid cancer. J Clin Oncol 2020;38:3594. https://doi.org/10.1200/jco.2020.38.15_suppl.3594.

[180] Czink E, Kloor M, Goeppert B, Fröhling S, Uhrig S, Weber TF, et al. Successful immune checkpoint blockade in a patient with advanced stage microsatellite-unstable biliary tract cancer. Cold Spring Harb Mol Case Stud 2017;3:a001974. https://doi.org/10.1101/mcs.a001974.

[181] Kojima T, Shah MA, Muro K, Francois E, Adenis A, Hsu C-H, et al. Randomized phase III KEYNOTE-181 study of pembrolizumab versus chemotherapy in advanced esophageal cancer. J Clin Oncol 2020;38:4138–48. https://doi.org/10.1200/JCO.20.01888.

[182] Janjigian YY, Bendell J, Calvo E, Kim JW, Ascierto PA, Sharma P, et al. CheckMate-032 study: Efficacy and safety of nivolumab and nivolumab plus ipilimumab in patients with metastatic esophagogastric cancer. J Clin Oncol 2018;36:2836–44. https://doi.org/10.1200/JCO.2017.76.6212.

[183] Capparelli C, Purwin TJ, Heilman SA, Chervoneva I, McCue PA, Berger AC, et al. ErbB3 targeting enhances the effects of MEK inhibitor in wild-type BRAF/NRAS melanoma. Cancer Res 2018;78:5680–93. https://doi.org/10.1158/0008-5472.CAN-18-1001.

[184] Patnaik A, Rosen LS, Tolaney SM, Tolcher AW, Goldman JW, Gandhi L, et al. Efficacy and safety of abemaciclib, an inhibitor of CDK4 and CDK6, for patients with breast cancer, non–small cell lung cancer, and other solid tumors. Cancer Discov 2016;6:740–53. https://doi.org/10.1158/2159-8290.cd-16-0095.

[185] Mazzaferro V, El-Rayes BF, Droz dit Busset M, Cotsoglou C, Harris WP, Damjanov N, et al. Derazantinib (ARQ 087) in advanced or inoperable FGFR2 gene fusion-positive intrahepatic cholangiocarcinoma. Br J Cancer 2019;120:165–71. https://doi.org/10.1038/s41416-018-0334-0.

[186] Wong JP, Todd JR, Finetti MA, McCarthy F, Broncel M, Vyse S, et al. Dual Targeting of PDGFRA and FGFR1 Displays Synergistic Efficacy in Malignant Rhabdoid Tumors. Cell Rep 2016;17:1265–75. https://doi.org/10.1016/j.celrep.2016.10.005.

[187] Das AB, Kakadia PM, Wojcik D, Pemberton L, Browett PJ, Bohlander SK, et al. Clinical remission following ascorbate treatment in a case of acute myeloid leukemia with mutations in TET2 and WT1. Blood Cancer J 2019;9:82. https://doi.org/10.1038/s41408-019-0242-4.

[188] Bejar R, Lord A, Stevenson K, Bar-Natan M, Pérez-Ladaga A, Zaneveld J, et al. TET2 mutations predict response to hypomethylating agents in myelodysplastic syndrome patients. Blood 2014;124:2705–12. https://doi.org/10.1182/blood-2014-06-582809.

[189] Subbiah V, Meyer C, Zinner R, Meric-Bernstam F, Zahurak ML, O’Connor A, et al. Phase Ib/II study of the safety and efficacy of combination therapy with multikinase VEGF inhibitor pazopanib and MEK inhibitor trametinib in advanced soft tissue sarcoma. Clin Cancer Res 2017;23:4027–34. https://doi.org/10.1158/1078-0432.CCR-17-0272.

[190] Tawbi HA, Burgess M, Bolejack V, Van Tine BA, Schuetze SM, Hu J, et al. Pembrolizumab in advanced soft-tissue sarcoma and bone sarcoma (SARC028): a multicentre, two-cohort, single-arm, open-label, phase 2 trial. Lancet Oncol 2017;18:1493–501. https://doi.org/10.1016/S1470-2045(17)30624-1.

[191] Wirth LJ, Sherman E, Robinson B, Solomon B, Kang H, Lorch J, et al. Efficacy of selpercatinib in RET-altered thyroid cancers. N Engl J Med 2020;383:825–35. https://doi.org/10.1056/NEJMoa2005651.

[192] Drilon A, Oxnard GR, Tan DSW, Loong HHF, Johnson M, Gainor J, et al. Efficacy of selpercatinib in RET fusion-positive non-small-cell lung cancer. N Engl J Med 2020;383:813–24. https://doi.org/10.1056/NEJMoa2005653.

[193] Sherman SI, Clary DO, Elisei R, Schlumberger MJ, Cohen EEW, Schöffski P, et al. Correlative analyses of RET and RAS mutations in a phase 3 trial of cabozantinib in patients with progressive, metastatic medullary thyroid cancer. Cancer 2016;122:3856–64. https://doi.org/10.1002/cncr.30252.

[194] Aparicio T, Cozic N, de la Fouchardière C, Meriaux E, Plaza J, Mineur L, et al. The activity of crizotinib in chemo-refractory MET-amplified esophageal and gastric adenocarcinomas: Results from the AcSé-crizotinib program. Target Oncol 2021;16:381–8. https://doi.org/10.1007/s11523-021-00811-8.

[195] Schmid P, Adams S, Rugo HS, Schneeweiss A, Barrios CH, Iwata H, et al. Atezolizumab and nab-paclitaxel in advanced triple-negative breast cancer. N Engl J Med 2018;379:2108–21. https://doi.org/10.1056/NEJMoa1809615.

[196] Parsons BM, Meier DR, Sreekumar S, Richmond CS, Ernzen KJ, Lofgren KA, et al. Abstract 1317: Exceptional responses to crizotinib in breast cancer patients with somatic MET and ROS1 alterations. Exp Mol Ther 2019. https://doi.org/10.1158/1538-7445.am2019-1317.

[197] Parsons BM, Meier DR, Gurda GT, Lofgren KA, Kenny PA. Exceptional Response to Crizotinib in an MET-Amplified Triple-Negative Breast Tumor. JCO Precis Oncol 2017:1–6. https://doi.org/10.1200/PO.17.00070.

[198] Mazières J, Barlesi F, Filleron T, Besse B, Monnet I, Beau-Faller M, et al. Lung cancer patients with HER2 mutations treated with chemotherapy and HER2-targeted drugs: results from the European EUHER2 cohort. Ann Oncol Off J Eur Soc Med Oncol 2016;27:281–6. https://doi.org/10.1093/annonc/mdv573.

[199] Fan Y, Chen J, Zhou C, Wang H, Shu Y, Zhang J, et al. Afatinib in patients with advanced non-small cell lung cancer harboring HER2 mutations, previously treated with chemotherapy: A phase II trial. Lung Cancer 2020;147:209–13. https://doi.org/https://doi.org/10.1016/j.lungcan.2020.07.017.

[200] Long Y, Zhang K, Li Y, Yu M, Zhu J, Huang M. Durable complete response after afatinib and crizotinib in an advanced non-small cell lung cancer patient with EGFR L861Q mutation and acquired MET amplification: a case report. Ann Palliat Med 2020;9:3609–13. https://doi.org/10.21037/apm-19-482.

[201] Li BT, Shen R, Buonocore D, Olah ZT, Ni A, Ginsberg MS, et al. Ado-trastuzumab emtansine for patients with HER2-mutant lung cancers: Results from a phase II basket trial. J Clin Oncol 2018;36:2532–7. https://doi.org/10.1200/JCO.2018.77.9777.

[202] Xue Y, Meehan B, Fu Z, Wang XQD, Fiset PO, Rieker R, et al. SMARCA4 loss is synthetic lethal with CDK4/6 inhibition in non-small cell lung cancer. Nat Commun 2019;10:557. https://doi.org/10.1038/s41467-019-08380-1.

[203] Li BT, Shen R, Offin M, Buonocore DJ, Myers ML, Venkatesh A, et al. Ado-trastuzumab emtansine in patients with HER2 amplified salivary gland cancers (SGCs): Results from a phase II basket trial. J Clin Oncol 2019;37:6001. https://doi.org/10.1200/JCO.2019.37.15_suppl.6001.

[204] Cohen RB, Delord J-P, Doi T, Piha-Paul SA, Liu S V, Gilbert J, et al. Pembrolizumab for the Treatment of Advanced Salivary Gland Carcinoma: Findings of the Phase 1b KEYNOTE-028 Study. Am J Clin Oncol 2018;41:1083–8. https://doi.org/10.1097/COC.0000000000000429.

[205] Rugo HS, Lerebours F, Ciruelos E, Drullinsky P, Ruiz-Borrego M, Neven P, et al. Alpelisib plus fulvestrant in PIK3CA-mutated, hormone receptor-positive advanced breast cancer after a CDK4/6 inhibitor (BYLieve): one cohort of a phase 2, multicentre, open-label, non-comparative study. Lancet Oncol 2021;22:489–98. https://doi.org/10.1016/S1470-2045(21)00034-6.

[206] André F, Ciruelos EM, Juric D, Loibl S, Campone M, Mayer IA, et al. Alpelisib plus fulvestrant for PIK3CA-mutated, hormone receptor-positive, human epidermal growth factor receptor-2-negative advanced breast cancer: final overall survival results from SOLAR-1. Ann Oncol 2021;32:208–17. https://doi.org/10.1016/j.annonc.2020.11.011.

[207] Doebele RC, Riely GJ, Spira AI, Horn L, Piotrowska Z, Costa DB, et al. First report of safety, PK, and preliminary antitumor activity of the oral EGFR/HER2 exon 20 inhibitor TAK-788 (AP32788) in non–small cell lung cancer (NSCLC). J Clin Oncol 2018;36:9015. https://doi.org/10.1200/jco.2018.36.15_suppl.9015.

[208] Jung M, Gelato KA, Fernández-Montalván A, Siegel S, Haendler B. Targeting BET bromodomains for cancer treatment. Epigenomics 2015;7:487–501. https://doi.org/10.2217/epi.14.91.

[209] Li X, Zhang XA, Xie W, Li X, Huang S. MYC-mediated synthetic lethality for treatment of hematological malignancies. Curr Cancer Drug Targets 2015;15:53–70. https://doi.org/10.2174/1568009615666150105120055.

[210] Wang J, Wang H, Peters M, Ding N, Ribback S, Utpatel K, et al. Loss of Fbxw7 synergizes with activated Akt signaling to promote c-Myc dependent cholangiocarcinogenesis. J Hepatol 2019;71:742–52. https://doi.org/10.1016/j.jhep.2019.05.027.

[211] Stein EM, DiNardo CD, Pollyea DA, Fathi AT, Roboz GJ, Altman JK, et al. Enasidenib in mutant IDH2 relapsed or refractory acute myeloid leukemia. Blood 2017;130:722–31. https://doi.org/10.1182/blood-2017-04-779405.

[212] Kopetz S, Grothey A, Yaeger R, Van Cutsem E, Desai J, Yoshino T, et al. Encorafenib, Binimetinib, and Cetuximab in BRAF V600E–Mutated Colorectal Cancer. N Engl J Med 2019;381:1632–43. https://doi.org/10.1056/NEJMoa1908075.

[213] Takahashi H, Tada Y, Saotome T, Akazawa K, Ojiri H, Fushimi C, et al. Phase II trial of trastuzumab and docetaxel in patients with human epidermal growth factor receptor 2-positive salivary duct carcinoma. J Clin Oncol 2019;37:125–34. https://doi.org/10.1200/JCO.18.00545.

[214] van Boxtel W, Boon E, Weijs WLJ, van den Hoogen FJA, Flucke UE, van Herpen CML. Combination of docetaxel, trastuzumab and pertuzumab or treatment with trastuzumab-emtansine for metastatic salivary duct carcinoma. Oral Oncol 2017;72:198–200. https://doi.org/10.1016/j.oraloncology.2017.06.023.

[215] Park JC, Ma TM, Rooper L, Hembrough T, Foss RD, Schmitt NC, et al. Exceptional responses to pertuzumab, trastuzumab, and docetaxel in human epidermal growth factor receptor-2 high expressing salivary duct carcinomas. Head Neck 2018;40:E100–6. https://doi.org/10.1002/hed.25392.

[216] Kim H, George E, Ragland R, Rafail S, Zhang R, Krepler C, et al. Targeting the ATR/CHK1 Axis with PARP Inhibition Results in Tumor Regression in BRCA-Mutant Ovarian Cancer Models. Clin Cancer Res an Off J Am Assoc Cancer Res 2017;23:3097–108. https://doi.org/10.1158/1078-0432.CCR-16-2273.

[217] Li MM, Datto M, Duncavage EJ, Kulkarni S, Lindeman NI, Roy S, et al. Standards and Guidelines for the Interpretation and Reporting of Sequence Variants in Cancer: A Joint Consensus Recommendation of the Association for Molecular Pathology, American Society of Clinical Oncology, and College of American Pathologists. J Mol Diagn 2017;19:4–23. https://doi.org/10.1016/j.jmoldx.2016.10.002.

**Table S4:** Detected mutations

| **PatID** | **Gene Symbol** | **DNA change** | **Amino acid change** | **Predicted effect** | **Variant Classification** | **Clinical relevance** | **pathogenicity** | **Allele fraction** | **Comment** |
| --- | --- | --- | --- | --- | --- | --- | --- | --- | --- |
| 1 | ERBB2 | c.2332_2340dup | p.G778_P780dup | gain |  | 2C | pathogenic | 0.33 |  |
| 1 | TP53 | c.783-1G>T |  |  | Splicing | 3 | pathogenic | 0.1 |  |
| 2 | PIK3CA | c.333G>C | p.K111N |  |  | 1A | pathogenic | 0.47 |  |
| 2 | TP53 | c.375G>C | p.T125T |  |  | 2C | likely pathogenic | 0.32 |  |
| 4 | MSH2 | c.1165C>T | p.R389* | loss | Stop gain | 2C | pathogenic | 0.22 |  |
| 4 | DNMT3A | c.2312G>A | p.R771Q | loss | Missense mutation | 2C | likely pathogenic | 0.06 |  |
| 4 | DAXX | c.472_493del | p.I158fs*3 | loss | Frameshift | 3 | likely pathogenic | 0.25 |  |
| 5 | JAK2 | c.3188G>A | p.R1063H | gain | Missense mutation | 2C | pathogenic | 0.47 |  |
| 5 | SH2B3 | c.622G>C | p.E208Q | loss | Missense mutation | 2C | pathogenic | 0.41 |  |
| 5 | STAT5B | c.1924A>C | p.N642H | gain | Missense mutation | 3 | pathogenic | 0.06 |  |
| 6 | MEN1 | c.1594G>T | p.G532C | loss | Missense mutation | 2C | likely pathogenic | 0.48 |  |
| 7 | IDH1 | c.394C>T | p.R132C | gain | Missense mutation | 1A | pathogenic | 0.23 |  |
| 7 | ARID1A | c.4993+2T>C |  | loss | Splicing | 2C | likely pathogenic | 0.42 |  |
| 7 | BAP1 | c.915dupT | p.E306 | loss | Frameshift mutation | 2C | likely pathogenic | 0.44 |  |
| 8 | BRAF | c.1794_1796dupTAC | p.T599dup | gain | In Frame Duplication | 2C | pathogenic | 0.2 |  |
| 8 | TP53 | c.746G>T | p.R249M | loss | Missense mutation | 2C | pathogenic | 0.28 |  |
| 8 | FAT1 | c.6437_6438insC | p.L2146fs*3 | loss | Frameshift mutation | 3 | pathogenic | 0.17 |  |
| 8 | SMAD4 | c.145_148dupGAGA | p.K50fs*5 | loss | Frameshift mutation | 3 | likely pathogenic | 0.19 |  |
| 9 | RB1 | c.1960G>A | p.V654M | loss | Splicing | 2C | pathogenic | 0.45 |  |
| 9 | POLE | c.893A>G | p.Y298C | loss | Missense mutation | 2C | likely pathogenic | 0.09 |  |
| 11 | RET | c.1946C>T | p.S649L | gain | Missense mutation | 2C | pathogenic | 0.81 |  |
| 11 | TP53 | c.782+1G>T |  | loss | Splicing | 2C | pathogenic | 0.93 |  |
| 12 | NRAS | c.181C>A | p.Q61K | gain | Missense mutation | 2C | pathogenic | 0.2 |  |
| 12 | TP53 | c.725G>A | p.C242Y | loss | Missense mutation | 2C | pathogenic | 0.51 |  |
| 13 | NRAS | c.181C>G | p.Gln61Lys |  |  | 3 | pathogenic | 0.09 |  |
| 14 | BRCA2 | c.6676_6677delGA | p.E2226fs*6 | loss | Frameshift | 1A | likely pathogenic | 0.25 |  |
| 14 | TP53 | c.374C>A | p.T125K | loss | Splicing | 2C | pathogenic | 0.6 |  |
| 14 | TSC2 | c.3206_3207delTG | p.V1069fs*98 | loss | Frameshift | 2C | pathogenic | 0.09 |  |
| 14 | ARID1A | c.215_216delAG | p.E72fs*38 | loss | Frameshift | 2C | likely pathogenic | 0.28 |  |
| 14 | MSH6 | c.1333_1334delAG | p.S445* | loss | Frameshift | 2C | likely pathogenic | 0.31 |  |
| 14 | NF1 | c.2341_2342delCA | p.H781fs*12 | loss | Frameshift | 2C | likely pathogenic | 0.39 |  |
| 14 | PPP2R2A | c.680_681delAG | p.E227fs*13 | loss | Frameshift | 2C | likely pathogenic | 0.5 |  |
| 14 | PTEN | c.647_648delTG | p.V216fs*26 | loss | Frameshift | 2C | likely pathogenic | 0.47 |  |
| 14 | PTEN | c.490_491delAA | p.K164fs*15 | loss | Frameshift | 2C | likely pathogenic | 0.3 |  |
| 14 | RASA1 | c.383_384delTC | p.L128fs*29 | loss | Frameshift | 2C | likely pathogenic | 0.49 |  |
| 14 | PIK3CA | c.3140A>G | p.H1047R | gain | Missense mutation | 2D | pathogenic | 0.41 |  |
| 14 | ARID1B | c.2434_2435delTC | p.S812fs*33 | loss | Frameshift | 3 | likely pathogenic | 0.37 |  |
| 14 | ARID2 | c.2395_2396delAG | p.H800fs*40 | loss | Frameshift | 3 | likely pathogenic | 0.47 |  |
| 14 | AXIN1 | c.662_663delGT | p.C221* | loss | Frameshift | 3 | likely pathogenic | 0.44 |  |
| 14 | BCOR | c.297_298delGA | p.K100fs*85 | loss | Frameshift | 3 | likely pathogenic | 0.24 |  |
| 14 | CUL3 | c.235_236delGA | p.E79fs*9 | loss | Frameshift | 3 | likely pathogenic | 0.31 |  |
| 14 | CUL3 | c.1864_1865delAG | p.R622fs*14 | loss | Frameshift | 3 | likely pathogenic | 0.37 |  |
| 14 | DHX15 | c.848_849delGA | p.R283fs*22 | loss | Frameshift | 3 | likely pathogenic | 0.41 |  |
| 14 | DNMT3B | c.296_297delAG | p.E99fs*113 | loss | Frameshift | 3 | likely pathogenic | 0.41 |  |
| 14 | FAT1 | c.4420_4421delGA | p.E1474fs*11 | loss | Frameshift | 3 | likely pathogenic | 0.72 |  |
| 14 | INPP4B | c.544_545delAG | p.S182fs*8 | loss | Frameshift | 3 | likely pathogenic | 0.28 |  |
| 14 | KMT2A | c.6655_6658delTACT | p.Y2219fs*23 | loss | Frameshift | 3 | likely pathogenic | 0.5 |  |
| 14 | KMT2A | c.4850_4581delTG | p.V1617fs*9 | loss | Frameshift | 3 | likely pathogenic | 0.38 |  |
| 14 | KMT2A | c.2641_2642delGA | p.E881fs*4 | loss | Frameshift | 3 | likely pathogenic | 0.3 |  |
| 14 | LATS1 | c.1173_1174del AG | p.G393fs*23 | loss | Frameshift | 3 | likely pathogenic | 0.46 |  |
| 14 | MAP3K13 | c.232_233delGT | p.78fs*17 | loss | Frameshift | 3 | likely pathogenic | 0.31 |  |
| 14 | MDC | c.2762_2763delGA | p.R921fs*94 | loss | Frameshift | 3 | likely pathogenic | 0.31 |  |
| 14 | NCOR1 | c.1249_1250delGA | p.E417fs*9 | loss | Frameshift | 3 | likely pathogenic | 0.36 |  |
| 14 | NOTCH3 | c.833_834delGT | p.C278fs*39 | loss | Frameshift | 3 | likely pathogenic | 0.38 |  |
| 14 | PRKCI | c.1033C>T | p.Q345* | loss | Stop Gain | 3 | likely pathogenic | 0.4 |  |
| 14 | RB1 | c.1512_1513delGA | p.N505fs*2 | loss | Frameshift | 3 | likely pathogenic | 0.5 |  |
| 14 | RPS6KB2 | c.1107_1108delCA | p.D369fs*3 | loss | Frameshift | 3 | likely pathogenic | 0.33 |  |
| 14 | SF3B1 | c.2427_2428delGA | p.E809fs*7 | loss | Frameshift | 3 | likely pathogenic | 0.06 |  |
| 14 | STK40 | c.120_121delTG | p.A41fs*25 | loss | Frameshift | 3 | likely pathogenic | 0.3 |  |
| 14 | TOP2A | c.3947_3948elAG | p.E1316fs*18 | loss | Frameshift | 3 | likely pathogenic | 0.37 |  |
| 14 | TRAF7 | c.1178_1179delTG | p.V393fs*16 | loss | Frameshift | 3 | likely pathogenic | 0.35 |  |
| 14 | U2AF1 | c.199+1_199+2delGT |  | loss | Splicing | 3 | likely pathogenic | 0.36 |  |
| 16 | BRCA2 | c.2590delC | p.Q864fs*10 | loss | Frameshift mutation | 2C | likely pathogenic | 0.07 |  |
| 16 | TSC1 | c.260delT | p.L87fs*11 | loss | Frameshift mutation | 2C | likely pathogenic | 0.33 |  |
| 17 | TP53 | c.637C>t | p.R213* | loss | Stop gain | 2C | pathogenic | 0.46 |  |
| 17 | ARID1A | c.6634C>T | p.Q2212* | loss | Stop gain | 2C | likely pathogenic | 0.42 |  |
| 17 | BRCA1 | c.4955T>C | p.M1652T | loss | Missense mutation | 2C | likely pathogenic | 0.5 |  |
| 17 | POLD1 | c.3068-1G>T |  | loss | Splicing | 2C | likely pathogenic | 0.19 |  |
| 17 | JAK2 | c.3188G>A | p.R1063H | gain | Missense mutation | 3 | pathogenic | 0.53 |  |
| 17 | PBRM1 | c.3275_3276delAG | p.E1092fs*5 | loss | Frameshift mutation | 3 | likely pathogenic | 0.44 |  |
| 18 | NF1 | c.3616G>T | p.E1206* | loss | Stop gain | 2C | pathogenic | 0.2 |  |
| 18 | TP53 | c.722C>T | p.S241F | gain | Missense mutation | 2C | pathogenic | 0.21 |  |
| 18 | POLE | c.2091delC | p.L698fs*94 | loss | Frameshift mutation | 2C | likely pathogenic | 0.52 |  |
| 18 | PBMR1 | c.1414C>T | p.472* | loss | Stop gain | 3 | pathogenic | 0.21 |  |
| 19 | EGFR Exon 20 | c.2303_2311dup | p.S768_770dup |  |  |  |  | 0.28 |  |
| 19 | PIK3CA | c.3129G>A | p.M1043I |  |  |  |  | 0.32 |  |
| 19 | PIK3CA | c.3133G>A | p.D1045N |  |  |  |  | 0.31 |  |
| 20 | PIK3CA | c.1633G>A | p.E545K | gain | Missense mutation | 2C | pathogenic | 0.09 |  |
| 20 | TP53 | c.965delC | p.P322fs*23 | loss | Frameshift | 2C | pathogenic | 0.2 |  |
| 20 | TET2 | c.5707C>T | p.Q1903* | loss | Stop Gain | 2C | likely pathogenic | 0.07 |  |
| 20 | PPP2R1A | c.547C>T | p.R183W | loss | Missense mutation | 3 | pathogenic | 0.15 |  |
| 21 | CDH1 | c.616_621del | p.I206_I207 del | loss | In-frame Deletion | 2C | likely pathogenic | 0.28 |  |
| 21 | BCOR | c.4274A>G | p.N1425S | loss | Missense mutation | 3 | likely pathogenic | 0.27 |  |
| 21 | ERBB4 | c.822A>C | p.Q274H | loss | Missense mutation | 3 | likely pathogenic | 0.1 |  |
| 22 | BAP1 | c.1437delC | p.T480fs*91 | loss | Frameshift mutation | 2C | likely pathogenic | 0.32 |  |
| 22 | PARP1 | c.718-1G>A |  | loss | Splicing | 2C | likely pathogenic | 0.12 |  |
| 22 | PRKN | c.823C>T | p.R275W | loss | Missense mutation | 3 | likely pathogenic | 0.64 |  |
| 23 | TP53 | c.743G>A | p.Arg248Gln | loss |  |  | pathogenic | 0.56 |  |
| 24 | FBXW7 | c.1513C>t | p.R505 |  | Missense mutation | 2C | pathogenic | 0.28 |  |
| 24 | KRAS | c.35G>C | p.G12A |  | Missense mutation | 2C | pathogenic | 0.23 |  |
| 24 | NRAS | c.182A>G | p.Q61R |  | Missense mutation | 2C | pathogenic | 0.36 |  |
| 24 | PIK3CA | c.3130A>G | p.H1047R |  | Missense mutation | 2C | pathogenic | 0.54 |  |
| 24 | BARD1 | c-1518_1519delTGinsCA | p.V507M |  | Missense mutation | 2C | likely pathogenic | 1 |  |
| 24 | APC | c.3340C>T | p.R1114* |  | Stop gain | 3 | pathogenic | 0.5 |  |
| 25 | FGFR3 | c.1150T>C | p.F384L | normal | Missense mutation | 2C | pathogenic | 0.43 |  |
| 25 | TET2 | c.2862G>A | p.W954* | loss | Stop Gain | 2C | pathogenic | 0.4 |  |
| 25 | TET2 | c.4272delT | p.D1425fs*23 | loss | Frameshift | 2C | likely pathogenic | 0.3 |  |
| 26 | IDH1 | c.394C>T | p.R132C | gain | Missense mutation | 1A | pathogenic | 0.24 |  |
| 28 | BRAF | c.1799T>A | p.V600E | gain | Missense mutation | 1A | pathogenic | 0.26 |  |
| 29 | PIK3CA | c.263G>A | p.R88Q |  |  | 2C | likely pathogenic | 0.14 |  |
| 29 | PIK3CA | c.3140A>G | p.H1047R | gain | Missense mutation | 2C | likely pathogenic | 0.16 |  |
| 31 | ALK | c.3586C>A | p.L1196M | gain | Missense mutation | 2C | pathogenic | 0.1 | second analysis |
| 32 | SMARCA4 | c.1548delG | p.R529fs*84 |  |  | 2C | likely pathogenic | 0.14 |  |
| 32 | NF1 | c.1400C>T | p.T467I |  |  | 3 | VUS | 0.11 |  |
| 33 | BRAF | c.1799T>A | p.V600E | gain | Missense mutation | 1A | pathogenic | 0.2 | second analysis |
| 33 | TP53 | c.818G>A | p.R273H | gain | Missense mutation | 2C | pathogenic | 0.2 | second analysis |
| 33 | KRAS | c.35G>A | p.G12D* | gain | Missense mutation |  | pathogenic | 0.25 |  |
| 34 | KRAS | c.34G>T | p.G12C | gain | Missense mutation | 2C | pathogenic | 0.41 |  |
| 34 | TP53 | c.746G>T | p.R249M | loss | Missense mutation | 2C | pathogenic | 0.5 |  |
| 34 | SMARCA4 | c.317dupT | p.M106fs*23 | loss | Frameshift | 2C | likely pathogenic | 0.36 |  |
| 34 | EPHA5 | c.2237-1G>C |  | loss | Splicing | 3 | pathogenic | 0.26 |  |
| 34 | AR | c.1738T>C | p.580R | loss | Missense mutation | 3 | likely pathogenic | 0.13 |  |
| 34 | CCN6 | c.27delC | p.S10fs*36 | loss | Frameshift | 3 | likely pathogenic | 0.24 |  |
| 35 | TP53 | c.626_627delGA | p.R209fs*6 | loss | Frameshift | 2C | pathogenic | 0.45 |  |
| 35 | ATRX | c.2612C>G | p.S871* | loss | Stop gain | 3 | VUS | 0.25 |  |
| 36 | KIT | c.1665_1679delACAGTGGAAGGTTGT | p.Q556_V560del | gain | In-frame Deletion | 1A | pathogenic | 0.9 |  |
| 37 | JAK2 | c.3188G>A | p.R1063H | gain | Missense mutation | 2C | pathogenic | 0.33 |  |
| 37 | TP53 | c.737T>C | p.M246T | loss | Missense mutation | 2C | pathogenic | 0.29 |  |
| 37 | PTEN | c.532_534delTAT | p.Y138del | loss | Frameshift | 2C | likely pathogenic | 0.31 |  |
| 38 | BRAF | c.1780G>A | p.D594N | loss | Missense mutation | 1A | pathogenic | 0.16 |  |
| 38 | BRCA2 | c.8940delA | p.E2981fs*7 | loss | Frameshift mutation | 1A | pathogenic | 0.15 |  |
| 38 | PBRM1 | c.1444-2A>G |  | loss | Splicing | 2D | likely pathogenic | 0.14 |  |
| 38 | GNAS | c.602G>A | p.R201H | gain | Missense mutation | 3 | pathogenic | 0.16 |  |
| 38 | FAT1 | c.12932delC | p.P4311fs*38 | loss | Frameshift mutation | 3 | likely pathogenic | 0.21 |  |
| 38 | FUBP1 | c.30delC | p.S11fs*43 | loss | Frameshift mutation | 3 | likely pathogenic | 0.17 |  |
| 38 | IRS2 | c.3272delC | p.P1091fs*15 | loss | Frameshift mutation | 3 | likely pathogenic | 0.21 |  |
| 39 | TP53 | c.1009C>T | p.R337C | loss | Missense mutation | 2C | pathogenic | 0.83 |  |
| 39 | APC | c.4666dupA | p.T1556fs*3 | loss | Frameshift | 3 | pathogenic | 0.67 |  |
| 39 | MUTYH | c.1103G>A | p.G368D | loss | Missense mutation | 3 | pathogenic | 0.55 |  |
| 42 | KIT | c.1727T>C | p.L576P | gain | Missense mutation | 1A | pathogenic | 0.35 |  |
| 42 | EP300 | c.4195G>A | p.D1399N | loss | Missense mutation | 2C | pathogenic | 0.07 |  |
| 42 | SF3B1 | c.1873C>T | p.R625C | loss | Missense mutation | 3 | pathogenic | 0.19 |  |
| 42 | PTPRT | c.4297C>T | p.R1433C | loss | Missense mutation | 3 | likely pathogenic | 0.06 |  |
| 43 | TP53 | c.524G>A | p.R175H | gain | Missense mutation | 2C | pathogenic | 0.23 |  |
| 43 | AR | p.R775C | p.R775C | loss | Missense mutation | 3 | pathogenic | 0.16 |  |
| 44 | ARID1A | c.2033dupA | p.S679fs*138 | loss | Frameshift mutation | 3 | likely pathogenic | 0.22 |  |
| 46 | TP53 | c.711G>A | p.M237I | loss | Missense mutation | 2C | pathogenic | 0.57 |  |
| 46 | APC | c.4067C>G | p.S1356* | loss | Stop gain | 3 | pathogenic | 0.68 |  |
| 46 | GRIN2A | c.1945C>G | p.L649V | loss | Missense mutation | 3 | pathogenic | 0.25 |  |
| 46 | PBRM1 | c.1291C>T | p.Q431* | loss | Stop gain | 3 | pathogenic | 0.66 |  |
| 46 | U2AF1 | c.101C>T | p.S34F | gain | Missense mutation | 3 | pathogenic | 0.18 |  |
| 46 | NTRK1 | c.1669C>T | p.R557W | loss | Missense mutation | 3 | likely pathogenic | 0.55 |  |
| 46 | SMAD3 | c.304G>T | p.E102* | loss | Stop gain | 3 | likely pathogenic | 0.65 |  |
| 47 | TP53 | c.742C>T | p.R248W | gain | Missense mutation | 2C | pathogenic | 0.46 |  |
| 47 | CDK12 | c.3107G>A | p.W1036* | loss | Stop gain | 2C | likely pathogenic | 0.15 |  |
| 47 | CREBBP | c.4169delC | p.P1390fs*69 | loss | Frameshift | 2C | likely pathogenic | 0.13 |  |
| 47 | TERT | c.-124C>T |  | gain |  | 3 | pathogenic | 0.48 |  |
| 47 | PPP2R1A | c.373G>T | p.E125* | loss | Stop gain | 3 | likely pathogenic | 0.06 |  |
| 48 | KRAS | c.30_35dupAGCTGG | p.A11_G12dup | gain | In-frame Insertion | 1A | pathogenic | 0.36 |  |
| 48 | ASXL1 | c.1534C>T | p.Q512* | loss | Stop gain | 2C | pathogenic | 0.18 |  |
| 48 | PIK3CA | c.1633G>A | p.E545K | gain | Missense mutation | 2C | pathogenic | 0.19 |  |
| 48 | APC | c.4666dupA | T1556fs*3 | loss | Frameshift | 3 | pathogenic | 0.28 |  |
| 48 | SMAD4 | c.1610A>G | p.D537G | loss | Missense mutation | 3 | pathogenic | 0.29 |  |
| 49 | TP53 | c.818G>A | p.R273H | gain | Missense mutation | 2C | pathogenic | 0.54 |  |
| 49 | ESR1 | c.1610A>C | p.Y537S | gain | Missense mutation | 2D | pathogenic | 0.41 |  |
| 49 | FOXP1 | 1.722+1G>T |  | loss | Splicing | 3 | pathogenic | 0.32 |  |
| 49 | GATA3 | c.1000_1001insC | p.N334fs*19 | loss | Frameshift | 3 | likely pathogenic | 0.42 |  |
| 50 | ATM | c.8631G>C | p.L2877F | loss | Missense mutation | 1A | likely pathogenic | 0.07 |  |
| 51 | FANCL | c.1096_1099dupATTA | p.T367fs*? | loss | Frameshift | 2C | pathogenic | 0.49 |  |
| 51 | TGFRB1 | c.902delG | p.G301fs*2 | loss | Frameshift | 3 | likely pathogenic | 0.09 |  |
| 52 | ATM | c.5346_5362dup | p.G1788fs*11 | loss | Frameshift | 2C | likely pathogenic | 0.19 |  |
| 53 | CDKN2A | c.71G>C | p.R24P | loss | Missense mutation | 2C | pathogenic | 0.68 |  |
| 53 | TERT | c.-124C>T |  | gain |  | 3 | pathogenic | 0.45 |  |
| 54 | BRCA1 | c.5206G>A | p.V1736I |  |  |  | likely pathogenic | 0.75 |  |
| 56 | KRAS | c.183A>C | p.Q61H | gain | Missense mutation | 1A | pathogenic | 0.34 |  |
| 56 | TP53 | c.844C>G | p.R282G | loss | Missense mutation | 2C | pathogenic | 0.9 |  |
| 56 | APC | c.4263delT | p.S1355fs*60 | loss | Frameshift | 3 | pathogenic | 0.25 |  |
| 56 | APC | c.4271delC | p.P1424fs*49 | loss | Frameshift | 3 | pathogenic | 0.59 |  |
| 56 | FH | c.1431_1433dupAAA | p.K477dup | loss | In-frame Deletion | 3 | likely pathogenic | 0.34 |  |
| 57 | TP53 | c.445delC | p.P152fs*18 | loss | Frameshift | 2C | pathogenic | 0.68 |  |
| 58 | ATRX | c.6514delG | p.E2172fs*9 | loss | Frameshift | 2C | likely pathogenic | 0.45 |  |
| 58 | FGFR2 | c.1620G>C | p.M540I | gain | Missense mutation | 3 | likely pathogenic | 0.39 |  |
| 58 | MUTYH | NM_001048171.1: c.283C>T ; NM_001048171.1: c.316C>T | NM_001048171.1: p.R95W; NM_001048171.1: p.R106W | loss | Missense mutation | 3 | likely pathogenic | 0.52 |  |
| 59 | TP53 | c.455C>T | p.P152L | loss | Missense mutation | 2C | pathogenic | 0.16 |  |
| 59 | NF2 | c.719_728delGGCTTCACAT | p.G240fs*8 | loss | Frameshift | 2C | likely pathogenic | 0.19 |  |
| 61 | ATRX | c.2785G>C | p.E292Q | loss | Missense mutation | 2C | likely pathogenic | 0.3 |  |
| 61 | TGFRB2 | c.383delA | p.K128fs*35 | loss | Frameshift | 3 | pathogenic | 0.08 |  |
| 63 | SETD2 | c.3278_3281del | p.S1093fs*27 | loss | Frameshift | 3 | pathogenic | 0.44 |  |
| 63 | SPEN | c.2194C>T | p.R732* | loss | Stop Gain | 3 | pathogenic | 0.85 |  |
| 64 | CDKN2A | c.238C>T | p.R80* | loss | Stop gain | 2C | pathogenic | 0.7 |  |
| 64 | ABL1 | c.740A>G | p.K247R | normal | Missense mutation | 2C | likely pathogenic | 0.16 |  |
| 64 | ATM | c.46G>T | p.E16* | loss | Stop gain | 2C | likely pathogenic | 0.71 |  |
| 64 | TERT | c.-146C>T |  | gain |  | 3 | pathogenic | 0.5 |  |
| 65 | ARID1A | c.1113dupG | p.Q372fs*28 | loss | Frameshift mutation | 2C | pathogenic | 0.15 |  |
| 65 | ATM | c.1564_1565delGA | p.E522fs*43 | loss | Frameshift mutation | 2C | pathogenic | 0.38 |  |
| 65 | ATM | c.8287C>T | p.R2763* | loss | Stop gain | 2C | pathogenic | 0.2 |  |
| 65 | KRAS | c.35G>A | p.G12D | gain | Missense mutation | 2C | pathogenic | 0.18 |  |
| 65 | SMAD2 | c.806C>G | p.S269* | loss | Stop gain | 3 | likely pathogenic | 0.15 |  |
| 69 | CEBPA | c.313_315dupGAC | p.D105dup | loss | In-frame Insertion |  | pathogenic | 0.59 |  |
| 69 | TP53 | c.436T>G | p.W146G | loss | Missense mutation |  | likely pathogenic | 0.65 |  |
| 69 | ATRX | c.2785G>C | p.E929Q | loss | Missense mutation |  | likely pathogenic | 0.97 |  |
| 70 | PIK3CA | c.1634A>C | p.E545A | gain | Missense mutation | 2C | pathogenic | 0.22 | second analysis |
| 70 | NF1 | c.4309G>A | p.E1437K | loss | Missense mutation | 2C | likely pathogenic | 0.34 | second analysis |
| 70 | PIK3CA | c.1359_1364delAGATTT | p.D454_L455del | loss | In Frame Deletion | 2C | likely pathogenic | 0.35 | second analysis |
| 71 | TP53 | c.763A>T | p.I255F | loss | Missense mutation | 2C | pathogenic | 0.22 |  |
| 71 | CDKN2A | c.220G>A | p.D74N | loss | Missense mutation | 2C | likely pathogenic | 0.2 |  |
| 71 | NTRK1 | c.1760G>A | p.G587D | gain | Missense mutation | 2C | likely pathogenic | 0.45 |  |
| 72 | IDH2 | c.516G>T | p.R172S |  | Missense mutation |  | pathogenic | 0.26 |  |
| 72 | ARID1B | c.5985T>G | p.Y1995* |  | Stop gain |  | pathogenic | 0.12 |  |
| 72 | MICA | c.953delG | p.G318fs |  | Frameshift mutation |  | likely pathogenic | 0.55 |  |
| 72 | BARD1 | c.1518_1519delinsCA | p.V507M |  | Missense mutation |  | likely pathogenic | 1 |  |
| 73 | TP53 | c.560-1G>A |  | loss | Splicing | 2C | pathogenic | 0.5 |  |
| 74 | KRAS | c.35G>A | p.G12D | gain | Missense mutation | 1A | pathogenic | 0.05 |  |
| 74 | TP53 | c.375+1_375+2insGCAGCTGTGACTTGCACGG |  | loss | Splicing | 2C | likely pathogenic | 0.08 |  |
| 74 | SMAD4 | c.670C>T | p.Q224* | loss | Stop Gain | 3 | likely pathogenic | 0.05 |  |
| 75 | ATM | c.5763-1G>T |  | loss | Splicing | 2C | likely pathogenic | 0.07 |  |
| 76 | ATRX | c.2785G>C | p.E929Q | loss | Missense mutation | 2C | likely pathogenic | 0.94 |  |
| 78 | SMAD4 | c.1081C>T | p.R361C | loss | Missense mutation | 3 | pathogenic | 0.13 |  |
| 79 | TP53 | c.796G>C | p.G266R |  |  |  | pathogenic | 0.72 |  |
| 81 | CDKN2A | c.355G>T | p.E119* | loss | Stop gain | 1A | likely pathogenic | 0.14 |  |
| 82 | FAT1 | c.6796G>T | p.E2266Ter | loss | Stop Gain | 3 | likely pathogenic | 0.37 |  |
| 83 | BRAF | c.1799T>A | p.V600E | gain | Missense mutation | 1A | pathogenic | 0.45 |  |
| 83 | TP53 | c.818G>A | p.R273H | gain | Missense mutation | 2C | pathogenic | 0.34 |  |
| 83 | BMPR1A | c.1508G>A | p.C503Y | loss | Missense mutation | 3 | likely pathogenic | 0.39 |  |
| 84 | ATM | c.2880delC | p.L961fs*10 | loss | Frameshift mutation | 2C | pathogenic | 0.35 |  |
| 84 | KRAS | c.35G>C | p.G12A | gain | Missense mutation | 2C | pathogenic | 0.4 |  |
| 84 | ATM | c.4741dupA | p.I1581fs*5 | loss | Frameshift mutation | 2C | likely pathogenic | 0.31 |  |
| 85 | PIK3CA | c.3140A>G | p.H1047R | gain | Missense mutation | 2C | pathogenic | 0.06 |  |
| 85 | TP53 | c.659A>G | p.Y220C | gain | Missense mutation | 2C | pathogenic | 0.07 |  |
| 86 | MTOR | c.5395G>A | p.E1799K | gain | Missense mutation | 2C | pathogenic | 0.34 |  |
| 86 | PIK3CA | c.1624G>A | p.E542K | gain | Missense mutation | 2C | pathogenic | 0.25 |  |
| 86 | TP53 | c.844C>T | p.R282W | gain | Missense mutation | 2C | pathogenic | 0.59 |  |
| 86 | LZTR1 | c.2284C>T | p.Q762* | loss | Stop gain | 3 | pathogenic | 0.76 |  |
| 86 | APC | c.3491_3492delTA | p.I1164fs*3 | loss | Frameshift | 3 | likely pathogenic | 0.51 |  |
| 87 | FGFR3 | c.1118A>G | p.Y373C | gain | Missense mutation | 2C | pathogenic | 0.6 |  |
| 87 | TP53 | c.460G>A | p.G154S | loss | Missense mutation | 2C | pathogenic | 0.23 |  |
| 89 | CHEK2 | c.599T>A | p.I200T | loss | Missense mutation | 2C | pathogenic | 0.53 |  |
| 89 | MET | c.504G>T | p.E168D | gain | Missense mutation | 3 | VUS | 0.55 |  |
| 90 | TP53 | c.844C>T | p.R282W | gain | Missense mutation | 2C | pathogenic | 0.36 |  |
| 90 | TERT | c.-124C>T |  | gain |  | 3 | pathogenic | 0.18 |  |
| 90 | RHOA | c.481G>A | p.A161T | gain | Missense mutation | 3 | likely pathogenic | 0.18 |  |
| 91 | BRAF | c.1799T>A | p.V600E | gain | Missense mutation | 1A | pathogenic | 0.35 |  |
| 92 | KRAS | c.35G>T | p.G12V | gain | Missense mutation | 2C | pathogenic | 0.36 |  |
| 92 | TP53 | c.814G>T | c.V272L | loss | Missense mutation | 2C | pathogenic | 0.15 |  |
| 92 | APC | c.4668delT | p.I1557fs*8 | loss | Frameshift | 3 | likely pathogenic | 0.18 |  |
| 93 | PIK3CA | c.1633G>A | p.E545K | gain | Missense mutation | 2C | pathogenic | 0.24 |  |
| 93 | TP53 | c.814G>T | p.V272L |  |  | 1A | pathogenic | 0.4 |  |
| 94 | TP53 | c.454_466del | p.P152fs*14 | loss | Frameshift mutation | 1A | pathogenic | 0.79 |  |
| 94 | IKBKE | c.1243_1248+13del |  | loss |  | 2C | likely pathogenic | 0.33 |  |
| 94 | CHD2 | c.393dupG | p.S132fs*2 | loss | Frameshift mutation | 3 | likely pathogenic | 0.42 |  |
| 94 | SMAD4 | c.1051G>T | p.D351Y | loss | Missense mutation | 3 | likely pathogenic | 0.43 |  |
| 95 | TP53 | c.659A>G | p.Y220C | gain | Missense mutation | 2C | pathogenic | 0.58 |  |
| 95 | NOTCH3 | c.1403dupT | p.D469fs*3 | loss | Frameshift | 3 | likely pathogenic | 0.17 |  |
| 95 | TP63 | c.580-1G>T |  | loss | Splicing | 3 | likely pathogenic | 0.47 |  |
| 96 | TP53 | c.1046_1063del | p.E349_Q354del | loss | In Frame Deletion | 2C | likely pathogenic | 0.64 | twice, 2 components |
| 96 | TP53 | c.1046_1063del | p.E349_Q354del | loss | In Frame Deletion | 2C | likely pathogenic | 0.28 | twice, 2 components |
| 96 | PIK3CA | c.3140A>G | p.H1047R | gain | Missense mutation | 2D | pathogenic | 0.13 |  |
| 97 | TP53 | c.743G>A | p.R248Q | gain | Missense mutation | 1A | pathogenic | 0.3 |  |
| 97 | ERBB3 | c.850G>A | p.G284R | gain | Missense mutation | 2C | pathogenic | 0.1 |  |
| 98 | KRAS | c.35G>A | p.G12D | gain | Missense mutation | 1A | pathogenic | 0.2 |  |
| 98 | TP53 | c.506C>G | p.Q136E | loss | Missense mutation | 1A | pathogenic | 0.32 |  |
| 98 | SMAD4 | c.1572GYT | p.W524C | loss | Missense mutation | 1A | pathogenic | 0.35 |  |
| 99 | NOTCH1 | c.7141C>T | p.Q2381Ter | gain | Stop gain | 3 | VUS | 0.97 |  |
| 99 | STAT5B | c.1924A>C | p.N642H | gain | Missense mutation | 3 | likely pathogenic | 0.94 |  |
| 100 | KRAS | c.183A>C | p.Q61H | gain | Missense mutation | 1A | pathogenic | 0.31 |  |
| 100 | TP53 | c.524G>A | p.R175H | gain | Missense mutation | 2C | pathogenic | 0.48 |  |
| 100 | BRIP1 | c.2327C>T | p.A776V | loss | Missense mutation | 2C | pathogenic | 0.55 |  |
| 101 | ARID2 | c.109dupA | p.I37fs*29 | loss | Frameshift | 3 |  | 0.57 |  |
| 101 | MICA | c.953delG | p.G318fs*? | loss | Frameshift | 3 |  | 0.47 |  |
| 102 | TP53 | c.644G>A | p.S215N | loss | Missense mutation | 1A | pathogenic | 0.85 |  |
| 102 | FBXW7 | c.45_46insCCT | p.T15_G16insP | loss | In-frame Insertion | 2C | likely pathogenic | 0.9 |  |
| 102 | APC | c.3927_3931delAAAGA | p.E1309fs*4 | loss | Frameshift | 2D | pathogenic | 0.25 |  |
| 102 | MICA | c.953_956delGCTG | p.G318fs* | loss | Frameshift | 3 | pathogenic | 0.32 |  |
| 102 | CIC | c.1979C>T | p.P660L | normal | Missense mutation | 3 | likely pathogenic | 0.64 |  |
| 103 | KRAS | c.34G>T | p.G12C | gain | Missense mutation |  | pathogenic | 0.22 |  |
| 103 | TP53 | c.527G>T | p.C176F | gain | Missense mutation |  | pathogenic | 0.19 |  |
| 103 | MUTYH | c.884C>T | p.295L | loss | Missense mutation |  | pathogenic | 0.53 |  |
| 103 | BARD1 | c.1518_1519delTGinsCA | p.V507M | gain | Missense mutation |  | likely pathogenic | 0.47 |  |
| 103 | APC | c.2985C>A | p.C995* | loss | Stop gain |  | pathogenic | 0.13 |  |
| 103 | APC | c.4630G>T | p.E1544* | loss | Stop gain |  | pathogenic | 0.16 |  |
| 103 | AMER1 | c.1801C>T | p.R601* | loss | Stop gain |  | pathogenic | 0.32 |  |
| 103 | SMAD4 | c.1180G>T | p.E394* | loss | Stop gain |  | pathogenic | 0.21 |  |
| 105 | KRAS | c.34G>A | p.G12S |  |  |  | pathogenic | 0.64 |  |
| 105 | TP53 | c.524G>A | p.R175H |  |  |  | pathogenic | 0.58 |  |
| 105 | TP53 | c.517G>C | p.V137L |  |  |  | pathogenic | 0.22 |  |
| 108 | PIK3CA | c.1624G>A | p.E542k | gain | Missense mutation | 1A | pathogenic | 0.46 | twice, 2 components |
| 108 | ATR | c.442G>T | p.E148* | loss | Stop gain | 2C | pathogenic | 0.43 | twice, 2 components |
| 108 | PIK3CA | c.1624G>A | p.E542k | gain | Missense mutation | 1A | pathogenic | 0.32 | twice, 2 components |
| 108 | ATR | c.442G>T | p.E148* | loss | Stop gain | 2C | pathogenic | 0.29 | twice, 2 components |
| 108 | CTNNA1 | c.2494dupA | p.T832fs*46 | loss | Frameshift | 3 | likely pathogenic | 0.4 |  |
| 109 | TP53 | c.817C>T | p.R273C | gain | Missense mutation | 1A | pathogenic | 0.7 |  |
| 109 | PIK3CA | c.1030G>A | p.V344M | loss | Missense mutation | 2C | pathogenic | 0.21 |  |
| 109 | PIK3CA | c.2176G>A | p.E726K | gain | Missense mutation | 2C | pathogenic | 0.26 |  |
| 110 | NF1 | c.1318C>T | p.R440* | loss | Stop gain | 1A | pathogenic | 0.51 |  |
| 110 | ATRX | c.3549_3567del | p.K1184fs*19 | loss | Frameshift | 2C | likely pathogenic | 0.64 |  |
| 111 | TP53 | c.332T>A | p.L111Q | loss | Missense mutation | 1A | pathogenic | 0.77 |  |
| 111 | APC | c.4062_4063delTT | p.S1355fs*19 | loss | Frameshift | 2D | pathogenic | 0.72 |  |
| 112 | TP53 | c.643A>T | p.S215G |  |  | 2C | pathogenic | 0.66 |  |
| 113 | BRAF | c.1799T>A | p.V600E | gain | Missense mutation | 1A | pathogenic | 0.28 |  |
| 113 | TP53 | c.534_536delCCA | p.H179del | loss | In-frame Deletion |  |  | 0.17 |  |
| 113 | SMAD4 | c.1081C>T | p.R361C | loss | Missense mutation | 3 | pathogenic | 0.22 |  |
| 114 | CREBBP | c.4650+2T>C |  | loss | Splicing | 2C | pathogenic | 0.06 |  |
| 114 | TET2 | c.1402_1405delCATG | p.H468fs*17 | loss | Frameshift | 2C | likely pathogenic | 0.29 |  |
| 114 | TET2 | c.5039_5040insTA | p.Q1680fs*16 | loss | Frameshift | 2C | likely pathogenic | 0.35 |  |
| 114 | PDCD1 | c.353_376del | p.S118_A125del | loss | In-frame Deletion | 3 | likely pathogenic | 0.77 |  |
| 115 | PIK3CA | c.1633G>C | p.E545Q | gain | Missense mutation | 2C | pathogenic | 0.25 |  |
| 115 | TP53 | c.814G>A | p.V272M | loss | Missense mutation | 2C | pathogenic | 0.28 |  |
| 115 | TERT | c.-124C>T |  | gain | Promoter mutation | 3 | pathogenic | 0.14 |  |
| 115 | NCOR1 | c.7042G>T | p.E2348* | loss | Stop Gain | 3 | likely pathogenic | 0.25 |  |
| 115 | RB1 | c.424_452del | p.T142fs*5 | loss | Frameshift | 3 | likely pathogenic | 0.1 |  |
| 117 | TP53 | c.508_527del | p.T170fs*4 | loss | Frameshift | 2C | pathogenic | 0.38 |  |
| 117 | RAD50 | c.551+1G>T |  | loss | Splicing | 2C | likely pathogenic | 0.28 |  |
| 117 | FGFR3 | c.1150T>C | p.F384L | normal | Missense mutation |  | VUS | 0.5 |  |
| 117 | AR | c.2105T>A | p.L702H | gain | Missense mutation | 2D | pathogenic | 0.36 |  |
| 117 | RAD21 | c.1162-1_1168del |  | loss | Splicing | 3 | likely pathogenic | 0.19 |  |
| 118 | KRAS | c.35G>T | p.G12V | gain | Missense mutation | 1A | pathogenic | 0.5 |  |
| 118 | PIK3CA | c.316G>C | p.G106R | loss | Missense mutation | 2C | pathogenic | 0.27 |  |
| 118 | APC | c.4335delA | p.A1446fs*27 | loss | Frameshift | 3 | pathogenic | 0.33 |  |
| 118 | PPM1D | c.1349delT | p.L450* | gain | Frameshift | 3 | pathogenic | 0.25 |  |
| 119 | CDKN2A | c.107delC | p.A36fs*17 | loss | Frameshift | 2C | pathogenic | 0.21 |  |
| 119 | CHEK2 | c.1427C>T | p.T476M | loss | Missense mutation | 2C | pathogenic | 0.19 |  |
| 119 | ERCC2 | c.262C>T | p.R88* | loss | Stop gain | 2C | likely pathogenic | 0.14 |  |
| 119 | FBXW7 | c.2066G>A | p.R689Q | loss | Missense mutation | 2C | likely pathogenic | 0.33 |  |
| 119 | TP53 | c.373_375+9del |  | loss | Promoter-Loss | 2C | pathogenic | 0.43 |  |
| 120 | TP53 | NM_000545.5:c[826_827dupGC;829T>G;832_833insT;837_848del12] | p.C277_R283delinsPVSG | loss | In-frame | 2C | likely pathogenic | 0.12 |  |
| 120 | RET | c.1759+1G>A |  | loss | Splicing | 2C | likely pathogenic | 0.19 |  |
| 120 | APC | c.3306C>G | p.Y1102* | loss | Stop gain | 3 | pathogenic | 0.26 |  |
| 120 | APC | c.3871C>T | p.Q1291* | loss | Stop gain | 3 | pathogenic | 0.28 |  |
| 120 | PPP2R1A | c.547C>T | p.R183W | loss | Missense mutation | 3 | pathogenic | 0.29 |  |
| 120 | CHD2 | c.1081_1097del17 | p.E361fs*9 | loss | Frameshift | 3 | likely pathogenic | 0.36 |  |
| 121 | PIK3CA | c.1035T>A | p.N345K | gain | Missense mutation | 2C | pathogenic | 0.26 |  |
| 121 | CTNNB1 | c.110C>T | p.S37F | gain | Missense mutation | 3 | pathogenic | 0.12 |  |
| 122 | RUNX1 | c.1003dupC | p.Q335fs*? | loss | Frameshift | 3 | likely pathogenic | 0.41 |  |
| 123 | NF1 | c.3709G>T | p.D1237Y | loss | Splicing | 1A | likely pathogenic | 0.06 |  |
| 123 | SDHA | c.767_768delCA | p.T256fs*64 | loss | Frameshift | 1A | likely pathogenic | 0.06 |  |
| 124 | TP53 | c.933-1G>A |  | loss | Splicing | 2C | pathogenic | 0.93 |  |
| 124 | TMEM127 | c.217G>C | p.G73R | loss | Missense mutation | 3 | likely pathogenic | 0.72 |  |
| 125 | BRAF | c.1798_1799delGTinsAA | p.V600K | gain | Missense mutation | 1A | pathogenic | 0.46 |  |
| 125 | PIK3CA | c.328_330delGAA | p.E110del | gain | In-frame Deletion | 2C | likely pathogenic | 0.07 |  |
| 125 | TP53 | c.1013C>T | p.F338S | loss | Missense mutation | 3 | pathogenic | 0.27 |  |
| 125 | CDC73 | c.760C>T | p.Q254* | loss | Stop gain | 3 | pathogenic | 0.3 |  |
| 125 | TERT | c.-124C>T |  | gain |  | 3 | pathogenic | 0.27 |  |
| 126 | TP53 | c.581T>G | p.L194R | gain | Missense mutation | 2C | pathogenic | 0.56 |  |
| 126 | APC | c.3920T>A | p.I1307K | loss | Missense mutation | 3 | pathogenic | 0.73 |  |
| 127 | ESR1 | c.1610A>G | p.Y537C | gain | Missense mutation | 1B | pathogenic | 0.25 |  |
| 127 | FOXP1 | c.1240delC | p.L414* | loss | Frameshift | 3 | pathogenic | 0.19 |  |
| 127 | FOXP1 | c.1349_1380del | p.A450fs*5 | loss | Frameshift | 3 | likely pathogenic | 0.13 |  |
| 128 | ARID1A | c.3826C>T | p.R1276 | loss | Stop gain | 2C | pathogenic | 0.29 |  |
| 128 | PIK3CA | c.1624G>A | p.E542K | gain | Missense mutation | 2C | pathogenic | 0.06 |  |
| 128 | TP53 | c.686_687delGT | p.C229fs*10 | loss | Frameshift | 2C | pathogenic | 0.34 |  |
| 128 | APC | c.1420_1423dupGCCA | p.I475fs*11 | loss | Frameshift | 2C | likely pathogenic | 0.31 |  |
| 128 | EGFR | c.2175G>A | p.T725T | loss | Synonymous | 3 | likely pathogenic | 0.48 |  |
| 129 | TP53 | c.412G>C | p.A138P | loss | Missense mutation | 2C | pathogenic | 0.1 |  |
| 129 | TP53 | c.706T>A | p.Y236N | loss | Missense mutation | 2C | pathogenic | 0.4 |  |
| 129 | MRE11 | c.1018G>T |  | loss | Splicing | 2C | likely pathogenic | 0.05 |  |
| 129 | SF3B1 | c.3481delA | p.M1161fs*9 | loss | Frameshift | 3 | likely pathogenic | 0.05 |  |
| 130 | KRAS | c.35G>T | p.G12V | gain | Missense mutation | 2C | pathogenic | 0.63 |  |
| 130 | KRAS | c.35G>T | p.G12V | gain | Missense mutation | 2C | pathogenic | 0.33 | second analysis |
| 130 | TP53 | c.831T>A | p.C277* | loss | Stop gain | 2C | pathogenic | 0.16 | second analysis |
| 130 | CDKN2A | c.122dupC | p.N42fs*2 | loss | Frameshift | 2C | likely pathogenic | 0.19 | second analysis |
| 131 | TP53 | c.818G>A | p.R273H | gain | Missense mutation | 2C | pathogenic | 0.76 |  |
| 131 | MLH1 | c.91_92delGCinsTG | p.A31C | loss | Missense mutation | 2C | likely pathogenic | 0.33 |  |
| 131 | SMARCA4 | c.3574C>T | p.R1192C | loss | Missense mutation | 2C | likely pathogenic | 0.41 |  |
| 132 | TP53 | c.994-1G>A |  | loss | Splicing | 2C | pathogenic | 0.53 |  |
| 132 | RB1 | c.1689G>C | p.W563C | loss | Missense mutation | 2C | likely pathogenic | 0.5 |  |
| 132 | APC | c.4666dupA | p.T1556fs*3 | loss | Frameshift | 3 | pathogenic | 0.3 |  |
| 132 | TGFBR2 | c.1159G>A | p.V387M | loss | Missense mutation | 3 | likely pathogenic | 0.11 |  |
| 133 | ARID1A | c.6259G>A | p.G2087R | loss | Missense mutation | 2C | pathogenic | 0.61 |  |
| 133 | CTNNB1 | c.134C>T | p.S45F | gain | Missense mutation | 2C | pathogenic | 0.34 |  |
| 133 | PTPN11 | c.1403C>T | p.T468M | loss | Missense mutation | 3 | pathogenic | 0.31 |  |
| 133 | PTPN11 | c.211T>C | p.F71L | gain | Missense mutation | 3 | pathogenic | 0.47 |  |
| 134 | KRAS | c.35G>A | p.G12D | gain | Missense mutation | 2C | pathogenic | 0.28 |  |
| 134 | ARID1A | c.3598C>T | p.Q1200* | loss | Stop gain | 2C | likely pathogenic | 0.09 |  |
| 134 | ATM | c.6154G>T | p.E2052* | loss | Stop gain | 2C | likely pathogenic | 0.11 |  |
| 134 | SMAD4 | c.1333C>T | p.R445* | loss | Stop gain | 3 | pathogenic | 0.09 |  |
| 135 | KRAS | c.35G>T | p.G12V | gain | Missense mutation | 2C | pathogenic | 0.11 |  |
| 136 | STK11 | c.647C>T | p.S216F | loss | Missense mutation | 2C | pathogenic | 0.2 |  |
| 137 | CDKN2A | c.235dupA | p.T79fs*41 | loss | Frameshift | 2C | pathogenic | 0.2 |  |
| 137 | PIK3CA | c.1624G>A | p.E542K | gain | Missense mutation | 2C | pathogenic | 0.06 |  |
| 137 | ARID1A | c.1114C>T | p.Q372* | loss | Stop gain | 2C | pathogenic | 0.13 |  |
| 137 | GNAS | c.602G>A | p.R201H | gain | Missense mutation | 3 | pathogenic | 0.08 |  |
| 138 | TP53 | c.837delG | p.R280fs*65 | loss | Frameshift | 2C | pathogenic | 0.68 |  |
| 139 | TP53 | c.743G>A | p.R248Q | gain | Missense mutation | 1A | pathogenic | 0.92 |  |
| 139 | RB1 | c.1215+1G>A |  | loss | Splicing | 2C | pathogenic | 0.88 |  |
| 139 | JAK3 | c.2969C>T | p.P990L | loss | Missense mutation | 2C | likely pathogenic | 0.14 |  |
| 140 | EP300 | c.631G>A | p.G211S | normal | Missense mutation |  |  | 0.54 |  |
| 140 | PTEN | c.1027-1G>A |  | loss | Splicing | 2C | pathogenic | 0.48 |  |
| 140 | PTEN | c.386G>T | p.G129V | loss | Missense mutation | 2C | pathogenic | 0.17 |  |
| 140 | ARID1A | c.803_839del | p.Q268fs*83 | loss | Frameshift | 2C | likely pathogenic | 0.42 |  |
| 140 | CTNNB1 | c.94G>T | p.D32Y | gain | Missense mutation | 2C | pathogenic | 0.34 |  |
| 140 | CTCF | c.592C>T | p.Q198* | loss | Stop gain | 3 | likely pathogenic | 0.29 |  |
| 140 | ZFHX3 | c.1507C>T | p.Q503* | loss | Stop gain | 3 | likely pathogenic | 0.3 |  |
| 142 | TP53 | c.524G>A | p.R175H | gain | Missense mutation | 2C | pathogenic | 0.95 |  |
| 142 | APC | c.3856G>T | p.E1286* | loss | Stop gain | 3 | pathogenic | 0.91 |  |
| 142 | AR | c.197A>T | p.Q66L | loss | Missense mutation | 3 | likely pathogenic | 0.7 |  |
| 143 | KRAS | c.183A>C | p.Q61H | gain | Missense mutation | 1A | pathogenic | 0.6 |  |
| 143 | TP53 | c.743G>A | p.R248Q | gain | Missense mutation | 1A | pathogenic | 0.44 |  |
| 143 | APC | c.4666delA | p.T1556fs*9 | loss | Frameshift | 3 | pathogenic | 0.4 |  |
| 144 | TP53 | c.412G>C | p.A138P | loss | Missense mutation | 2C | pathogenic | 0.9 |  |
| 145 | KIT | c.1667_1674del | p.Y553_K558del | gain | In-frame Deletion | 1A | pathogenic | 0.47 |  |
| 145 | KIT | c.2467T>C | p.Y823H | gain | Missense mutation | 1A | likely pathogenic | 0.11 |  |
| 145 | KIT | c.2467T>A | p.Y823N | loss | Missense mutation | 1A | likely pathogenic | 0.5 |  |
| 146 | PIK3CA | c.1624G>A | p.E542K | gain | Missense mutation | 2C | pathogenic | 0.18 |  |
| 146 | ARID1A | c.5420_5421insA | p.S1807fs*2 | loss | Frameshift | 2C | likely pathogenic | 0.15 |  |
| 146 | PIK3CA | c.1133G>T | p.C378F | gain | Missense mutation | 2C | likely pathogenic | 0.19 |  |
| 146 | BCOR | c.4076_4086del | p.T1359fs*12 | loss | Frameshift | 3 | likely pathogenic | 0.44 |  |
| 147 | TP53 | c.646G>A | p.V216M | loss | Missense mutation | 2C | pathogenic | 0.81 |  |
| 147 | ATRX | c.3523C>T | p.Q1175* | loss | Stop gain | 2C | likely pathogenic | 0.3 |  |
| 147 | APC | c.4063dupT | p.S1355fs*20 | loss | Frameshift | 3 | pathogenic | 0.82 |  |
| 147 | RBM10 | c.274C>T | p.Q92* | loss | Stop Gain | 3 | likely pathogenic | 0.26 |  |
| 148 | TP53 | c.524G>T | p.R175L | loss | Missense mutation | 2C | pathogenic | 0.48 |  |
| 148 | RUNX1 | c.1255dupC | p.L419PfsTer154 | loss | Frameshift | 3 | likely pathogenic | 0.37 |  |
| 149 | ERBB3 | c.2783A>G | p.E928G | gain | Missense mutation | 2C | pathogenic | 0.1 |  |
| 149 | NAB2 | c.1140delA | p.E381fs*72 | loss | Frameshift | 3 | likely pathogenic | 0.45 |  |
| 149 | SPEN | c.4423C>T | p.R1475* | loss | Stop gain | 3 | likely pathogenic | 0.32 |  |
| 150 | KRAS | c.35G>A | p.G12D | gain | Missense mutation | 1A | pathogenic | 0.03 |  |
| 150 | KRAS | c.35G>A | p.G12D | gain | Missense mutation | 1A | pathogenic | 0.03 |  |
| 151 | SETD2 | c.412G>T | p.E138* | loss | Stop Gain | 3 | likely pathogenic | 0.12 |  |
| 152 | KRAS | c.35G>T | p.G12V | gain | Missense mutation | 2C | pathogenic | 0.49 |  |
| 152 | TP53 | c.626_627delGA | p.R209fs*6 | loss | Frameshift | 2C | pathogenic | 0.62 |  |
| 153 | ASLX1 | c.1894C>T | p.R632* | loss | Stop gain | 2C | pathogenic | 0.52 |  |
| 153 | PIK3CA | c.1633G>A | p.E545K | gain | Missense mutation | 2C | pathogenic | 0.26 |  |
| 153 | NSD1 | c.1847C>G | p.S616 | loss | Stop gain | 3 | likely pathogenic | 0.34 |  |
| 154 | GNAQ | c.548G>A | p.R183Q | gain | Missense mutation | 2C | pathogenic | 0.85 |  |
| 155 | EZH2 | c.589delG | p.E197fs*44 | loss | Frameshift | 2C | likely pathogenic | 0.06 |  |
| 156 | FGFR3 | c.742C>T | p.R248C |  |  |  | pathogenic | 0.46 |  |
| 157 | KRAS | c.34G>A | p.G12S | gain | Missense mutation | 1A | pathogenic | 0.56 |  |
| 157 | TP53 | c.524G>A | p.R175H | gain | Missense mutation | 2C | pathogenic | 0.5 |  |
| 157 | APC | c.694C>T | p.R232* | loss | Stop gain | 3 | pathogenic | 0.56 |  |
| 157 | XPO1 | c.1711G>A | p.E571K | gain | Missense mutation | 3 | pathogenic | 0.1 |  |
| 157 | TCF7L2 | c.932+1G>A |  | loss | Splicing | 3 | likely pathogenic | 0.35 |  |
| 158 | KRAS | c.35G>T | p.G12V | gain | Missense mutation | 2C | pathogenic | 0.36 |  |
| 158 | ARID1A | c.3670delA | p.M1224fs*13 | loss | Frameshift | 2C | likely pathogenic | 0.27 |  |
| 158 | CDKN2A | c.214delT | p.C72fs*74 | loss | Frameshift | 2C | likely pathogenic | 0.46 |  |
| 158 | TP53 | c.829_849del | p.C277_R832del | loss | In-frame Deletion | 2C | likely pathogenic | 0.32 |  |
| 159 | KRAS | c.35G>A | p.G12D | gain | Missense mutation | 2C | pathogenic | 0.41 |  |
| 159 | PIK3CA | c.1624G>A | p.E542K | gain | Missense mutation | 2C | pathogenic | 0.23 |  |
| 159 | TP53 | c.725G>T | p.C242F | loss | Missense mutation | 2C | pathogenic | 0.53 |  |
| 159 | MUTYH | c.452 | p.Y151C | loss | Missense mutation | 3 | pathogenic | 1 |  |
| 159 | APC | c.673G>T | p.E225* | loss | Missense mutation | 3 | likely pathogenic | 0.52 |  |
| 160 | APC | c.4059delA | p.E1353fs*62 | loss | Frameshift | 3 | pathogenic | 0.26 |  |
| 160 | PTPN11 | c.1508G>A | p.G503E | gain | Missense mutation | 3 | pathogenic | 0.1 |  |
| 160 | NSD1 | c.4030A>T | p.K1344* | loss | Stop gain | 3 | likely pathogenic | 0.3 |  |
| 161 | BRAF | c.1799T>A | p.V600E | gain | Missense mutation | 1A | pathogenic | 0.35 |  |
| 162 | PTEN | c.388C>T | p.R130* | loss | Stop gain | 2C | pathogenic | 0.07 |  |
| 162 | TP53 | c.314G>T | p.G105V | loss | Missense mutation | 2C | pathogenic | 0.69 |  |
| 162 | SDHA | c.779C>T | p.T260M | loss | Missense mutation | 2C | likely pathogenic | 0.26 |  |
| 162 | CASP8 | c.595+1G>C |  | loss | Splicing | 3 | likely pathogenic | 0.4 |  |
| 163 | KRAS | c.38G>A | p.G13D | gain | Missense mutation | 1A | pathogenic | 0.55 |  |
| 163 | MAP2K1 | c.199G>A | p.D67N | gain | Missense mutation | 2C | pathogenic | 0.33 |  |
| 163 | PIK3CA | c.1633G>A | p.E545K | gain | Missense mutation | 2C | pathogenic | 0.32 |  |
| 163 | TET2 | c.1648C>T | p.R550* | loss | Stop gain | 2C | pathogenic | 0.12 |  |
| 163 | ARID1A | c.2502dupC | p.M835fs*37 | loss | Frameshift | 2C | likely pathogenic | 0.32 |  |
| 163 | NTRK1 | c.1837C>T | p.R613W | loss | Missense mutation | 2C | likely pathogenic | 0.16 |  |
| 163 | TP53 | c.806G>A | p.S269N | loss | Missense mutation | 2C | likely pathogenic | 0.47 |  |
| 163 | APC | c.4348C>T | p.R1450* | loss | Stop gain | 3 | pathogenic | 0.59 |  |
| 163 | TGFBR2 | c.1528C>T | p.R528C | loss | Missense mutation | 3 | pathogenic | 0.67 |  |
| 163 | AR | c.451G>A | p.A151T | loss | Missense mutation | 3 | likely pathogenic | 0.18 |  |
| 163 | SOX9 | c.886C>T | p.Q296* | loss | Stop gain | 3 | likely pathogenic | 0.66 |  |
| 164 | ERBB2 | c.2263_2264delTTinsCC | p.L755P | gain | Missense mutation | 2C | pathogenic | 0.45 |  |
| 164 | MSH6 | c.2335dupT | p.C779fs*6 | loss | Frameshift | 2C | likely pathogenic | 0.51 |  |
| 165 | KRAS | c.34G>A | p.G12S | gain | Missense mutation | 1A | pathogenic | 0.26 |  |
| 165 | FBXW7 | c.1394G>A | p.R465H | loss | Missense mutation | 2C | pathogenic | 0.23 |  |
| 165 | APC | c,2626C>T | p.R876* | loss | Stop Gain | 3 | pathogenic | 0.24 |  |
| 165 | APC | c.4478_4479insACAC | p.E1494fs*21 | loss | Frameshift | 3 | likely pathogenic | 0.22 |  |
| 166 | CTNNB1 | c.110C>G | p.Ser37Cys |  |  |  | pathogenic | 0.18 |  |
| 166 | PIK3CA | c.1633G>A | p.Glu545Lys |  |  |  | pathogenic | 0.06 |  |
| 166 | EGFR | c.2235_2249del | p.Glu746_Ala750del | gain |  |  | pathogenic | 0.22 |  |
| 167 | KIT | c.1660G>A | p.E554K |  |  |  | pathogenic |  |  |
| 168 | SMARCB1 | c.1121G>A | p.R374Q | loss | Missense mutation | 2C | pathogenic | 0.3 |  |
| 169 | TERT | c,.-146C>T |  | gain |  | 3 | pathogenic | 0.18 |  |
| 169 | HRAS | c.32_37dupCCGGCG | p.A11_G12dup | loss | in-frame Duplication | 2C | VUS | 0.14 |  |
| 171 | KRAS | c.35G>T | p.G12V | gain | Missense mutation | 2C | pathogenic | 0.23 |  |
| 171 | TP53 | c.395A>G | p.K132R | loss | Missense mutation | 2C | pathogenic | 0.53 |  |
| 172 | MET | c.3082G>A | p.Asp1028Asn | gain |  |  | likely pathogenic | 0.62 |  |
| 173 | AMER1 | c.853_860del | p.His285ArgfsTer36 | loss | Frameshift | 3 | likely pathogenic | 0.56 |  |
| 173 | GRIN2A | c.1903G>A | p.A635T | loss | Missense mutation | 3 | likely pathogenic | 0.23 |  |
| 174 | TP53 | c.742C>G | p.R248G | gain | Missense mutation | 2C | pathogenic | 0.76 |  |
| 174 | APC | c.3391C>T | p.Q1131Ter | loss | Stop gain | 3 | pathogenic | 0.23 |  |
| 175 | TP53 | c.560-2A>C |  | loss | Splicing | 2C | pathogenic | 0.53 |  |
| 175 | FANCA | c.3764A>C | p.E1255A | loss | Splicing | 3 | pathogenic | 0.71 |  |
| 175 | SPTA1 | c.5190-1G>T |  | loss | Splicing | 3 | pathogenic | 0.16 |  |
| 175 | RB1 | c.2590G>T | p.E864* | loss | Stop gain | 3 | likely pathogenic | 0.6 |  |
| 176 | KRAS | c.35G>T | p.G12V | gain | Missense mutation | 2C | pathogenic | 0.39 |  |
| 176 | TP53 | c.584T>C | p.I195T | loss | Missense mutation | 2C | pathogenic | 0.35 |  |
| 176 | SMAD4 | c.1324C>T | p.Q442* | loss | Stop gain | 3 | pathogenic | 0.37 |  |
| 178 | KRAS | c.34_35delinsTT | p.Gly12Phe |  |  |  | pathogenic | 0.54 |  |
| 178 | TP53 | c.810dupT | p.Glu271Ter |  |  |  | pathogenic | 0.42 |  |
| 178 | TP53 | c.711G>T | p.Met237Ile |  |  |  | likely pathogenic | 0.2 |  |
| 180 | KRAS | c.35G>A | p.G12D | gain | Missense mutation | 1A | pathogenic | 0.47 |  |
| 180 | TP53 | c.524G>A | p.R175H | gain | Missense mutation | 2C | pathogenic | 0.85 |  |
| 180 | APC | c.4175C>A | p.S1392* | loss | Stop gain | 3 | pathogenic | 0.27 |  |
| 180 | APC | c.847C>T | p.R283* | loss | Stop gain | 3 | pathogenic | 0.45 |  |
| 180 | SMAD4 | c.307delG | p.D103fs*7 | loss | Frameshift | 3 | pathogenic | 0.72 |  |
| 180 | TCF7L2 | c.1211delA | p.K404fs*20 | loss | Frameshift | 3 | pathogenic | 0.39 |  |
| 181 | KRAS | c.35G>T | p.G12V | gain | Missense mutation | 2C | pathogenic | 0.23 |  |
| 182 | TP53 | c.742C>G | p.R248G | gain | Missense mutation | 2C | pathogenic | 0.85 |  |
| 182 | SMO | c.1016G>A | p.W339* | loss | Stop gain | 3 | pathogenic | 0.63 |  |
| 183 | IDH1 | c.394C>T | p.R132C | gain | Missense mutation | 1A | pathogenic | 0.22 |  |
| 184 | EGFR | c.323G>A | p.R108K | gain | Missense mutation | 2C | pathogenic | 0.28 |  |
| 184 | KRAS | c.35G>T | p.G12V | gain | Missense mutation | 2C | pathogenic | 0.22 |  |
| 184 | CSF1R | c.1897G>A | p.E633K | loss | Missense mutation | 2C | pathogenic | 0.21 |  |
| 184 | GNAS | c.601C>T | p.R201C | gain | Missense mutation | 3 | pathogenic | 0.21 |  |
| 184 | SMAD4 | c.1082G>A | p.R361H | gain | Missense mutation | 3 | pathogenic | 0.26 |  |
| 185 | VHL | c.433C>T | p.Q145Ter | loss | Stop Gain | 1A | pathogenic | 0.08 |  |
| 185 | BAP1 | c.146T>C | p.L49P | loss | Missense mutation | 2C | likely pathogenic | 0.1 |  |
| 185 | TERT | c.-146C>T |  | gain |  | 3 | pathogenic | 0.09 |  |
| 186 | KRAS | c.35G>A | p.G12D | gain | Missense mutation | 1A | pathogenic | 0.61 |  |
| 186 | TP53 | c.725G>A | p.C242Y | loss | Missense mutation | 2C | pathogenic | 0.76 |  |
| 186 | APC | c.4009C>T | p.Q1367Ter | loss | Stop Gain | 3 | pathogenic | 0.51 |  |
| 186 | APC | c.2240C>G | p.S747Ter | loss | Stop Gain | 3 | pathogenic | 0.21 |  |
| 186 | MUTYH | c.1103G>A | p.G368D | loss | Missense mutation | 3 | pathogenic | 0.47 |  |
| 186 | PTPRT | c.1090C>T | p.R364Ter | loss | Stop Gain | 3 | likely pathogenic | 0.36 |  |
| 187 | PIK3CA | c.1633G>A | p.E545K | gain | Missense mutation | 2C | pathogenic | 0.28 |  |
| 187 | U2AF1 | c.101C>T | p.S34F | gain | Missense mutation | 3 | pathogenic | 0.25 |  |
| 188 | BRCA2 | c.7913_7917delTTCTCT | p.Phe2638Ter |  |  |  |  | 0.96 | external analysis |
| 188 | CDKN2A | c.238_248delCGACCCGTGA | p.Pro81fs |  |  |  |  | 0.5 | external analysis |
| 189 | ATRX | c.4630dupA | p.T1554fs*19 | loss | Frameshift | 2C | pathogenic | 0.3 |  |
| 189 | RAD51C | c.859A>G | p.T287A | loss | Missense mutation | 2C | pathogenic | 0.15 |  |
| 189 | BAP1 | c.1891G>T | p.E631* | loss | Splicing | 2C | likely pathogenic | 0.65 |  |
| 189 | TP53 | c.704delA | p.N235fs*12 | loss | Frameshift | 2C | likely pathogenic | 0.52 |  |
| 190 | BRAF | c.1799T>A | p.V600E | gain | Missense mutation | 1A | pathogenic | 0.69 |  |
| 190 | PIK3CA | c.1624G>A | p.E542K | gain | Missense mutation | 2C | pathogenic | 0.6 |  |
| 190 | TP53 | c.963_966dupACCA | p.L323fs*15 | loss | Frameshift | 2C | likely pathogenic | 0.6 |  |
| 190 | BMPR1A | c.176dupT | p.L59fs*12 | loss | Frameshift | 3 | likely pathogenic | 0.35 |  |
| 191 | TP53 | c.1006G>T | p.E336* | loss | Stop Gain | 2C | pathogenic | 0.78 |  |
| 191 | EP300 | c.631G>A | p.G211S | loss | Missense mutation | 2C | likely pathogenic | 0.36 |  |
| 192 | PIK3CA | c.1624G>A | p.E542K | gain | Missense mutation | 2C | pathogenic | 0.44 |  |
| 192 | APC | c.4057G>T | p.E135* | loss | Stop Gain | 2D | pathogenic | 0.82 |  |
| 192 | MUTYH | c.1103G>A | p.G368D | loss | Missense mutation | 3 | pathogenic | 0.47 |  |
| 192 | TCF7L2 | c.1391+1G>T |  | loss | Splicing | 3 | likely pathogenic | 0.34 |  |
| 193 | TP53 | c.844C>T | p.R282W | gain | Missense mutation | 2C | pathogenic | 0.63 |  |
| 195 | TP53 | c.754_762delCTCACCATC | p.L252_L254del | loss | In-frame Deletion | 2C | likely pathogenic | 0.23 |  |
| 195 | SMAD4 | c.1157G>T | p.G386V | loss | Missense mutation | 3 | likely pathogenic | 0.15 |  |
| 196 | KRAS | c.35G>A | p.G12D | gain | Missense mutation | 1A | pathogenic | 0.06 |  |
| 196 | TP53 | c.375G>A | p.T125T | loss | Splicing | 2C | pathogenic | 0.13 |  |
| 196 | CHEK2 | c.1196C>T | p.S399L | loss | Missense mutation | 2C | likely pathogenic | 0.49 |  |
| 197 | KRAS | c.34G>A | p.G12S | gain | Missense mutation | 1A | pathogenic | 0.32 |  |
| 197 | FANCA | c.3002C>A | p.S1001Ter | loss | Stop Gain | 2C | likely pathogenic | 0.49 |  |
| 197 | TP53 | c.817C>T | p.R273C | gain | Missense mutation | 2C | likely pathogenic | 0.44 |  |
| 197 | APC | c.4731delA | p.E1577fs*73 | loss | Frameshift | 3 | pathogenic | 0.23 |  |
| 198 | TP53 | c.776A>T | p.D259V | loss | Missense mutation | 2C | pathogenic | 0.46 |  |
| 198 | HGF | c.1048C>T | p.R350Ter | loss | Stop Gain | 3 | likely pathogenic | 0.17 |  |
| 198 | NKX2 | c.685dupC | p.L229fs*? | loss | Frameshift | 3 | likely pathogenic | 0.49 |  |
| 199 | KRAS | c.34G>A | p.G12S | gain | Missense mutation | 1A | pathogenic | 0.8 |  |
| 199 | NBN | c.265A>G | p.I89V | loss | Missense mutation | 2C | pathogenic | 0.73 |  |
| 199 | TP53 | c.527G>T | p.C176F | gain | Missense mutation | 2C | pathogenic | 0.77 |  |
| 199 | APC | c.4348C>T | p.R1450* | loss | Stop gain | 3 | pathogenic | 0.34 |  |
| 199 | APC | c.637C>T | p.R213* | loss | Stop gain | 3 | pathogenic | 0.27 |  |
| 199 | AR | c.203A>T | p.Q68L | loss | Missense mutation | 3 | likely pathogenic | 0.2 |  |
| 200 | HOXB13 | c.64G>T | p.G22* | loss | Stop gain | 3 | likely pathogenic | 0.15 |  |
| 200 | TRAF7 | c.64_75del | p.P22_T25del | loss | In-frame Deletion | 3 | likely pathogenic | 0.34 |  |
| 201 | TP53 | c.733G>A | p.G245S | gain | Missense mutation | 2C | pathogenic | 0.66 |  |
| 202 | KRAS | c.437C>T | p.A146V | gain | Missense mutation | 1A | pathogenic | 0.49 |  |
| 202 | ATM | c.67C>T | p.R23* | loss | Stop gain | 2C | pathogenic | 0.21 |  |
| 202 | FBXW7 | c.1393C>T | p.R465C | loss | Missense mutation | 2C | pathogenic | 0.26 |  |
| 202 | PIK3CA | c.3140A>G | p.H1047 | gain | Missense mutation | 2D | pathogenic | 0.25 |  |
| 202 | APC | c.3935delG | p.G1312fs*9 | loss | Frameshift | 3 | pathogenic | 0.23 |  |
| 202 | APC | c.834+1G>A |  | loss | Splicing | 3 | pathogenic | 0.21 |  |
| 203 | ESR1 | c.1609T>A | p.Y537N | gain | Missense mutation | 1B | pathogenic | 0.75 |  |
| 203 | PIK3CA | c.3140A>T | p.H1047L | gain | Missense mutation | 2C | pathogenic | 0.71 |  |
| 203 | EGFR | c.1244C>T | p.T415M | gain | Missense mutation | 2C | likely pathogenic | 0.19 |  |
| 204 | KRAS | c.183A>C | p.Q61H | gain | Missense mutation | 1A | pathogenic | 0.09 |  |
| 204 | STK11 | c.207_239del | p.E70L80del | loss | In-frame Deletion | 2C | likely pathogenic | 0.17 |  |
| 205 | AKT1 | c.49G>A | p.E17K | gain | Missense mutation | 2C | pathogenic | 0.79 |  |
| 205 | FANCE | c.929dupC | p.V311fs*2 | loss | Frameshift | 2C | pathogenic | 0.55 |  |
| 205 | STK11 | c.7delG | p.V3fs*15 | loss | Frameshift | 2C | pathogenic | 0.82 |  |
| 205 | ATM | c.1235+3A>G |  | loss | Intron Variant | VUS | likely pathogenic | 0.56 |  |
| 206 | CDK12 | c.1811delC | p.P604fs*6 | loss | Frameshift | 1A | likely pathogenic | 0.3 |  |
| 206 | PTEN | c.968dupA | p.N323fs*2 | loss | Frameshfit | 2C | pathogenic | 0.25 |  |
| 206 | PTEN | c.445C>T | p.Q149* | loss | Stop Gain | 2C | pathogenic | 0.16 |  |
| 206 | CREBBP | c.3790dupT | p.W1264fs*26 | loss | Frameshift | 2C | likely pathogenic | 0.2 |  |
| 206 | JAK1 | c.2580delA | p.K860fs*16 | loss | Frameshift | 2C | likely pathogenic | 0.4 |  |
| 206 | POLD1 | c.331delG | p.V111fs*58 | loss | Frameshift | 2C | likely pathogenic | 0.22 |  |
| 206 | AXIN1 | c.792delC | p.G265fs*149 | loss | Frameshift |  |  | 0.2 |  |
| 206 | ARID1B | c.2791G>T | p.G931* | loss | Stop Gain | 3 | likely pathogenic | 0.16 |  |
| 206 | AXIN1 | c.234delC | p.T79fs*5 | loss | Frameshift | 3 | likely pathogenic | 0.07 |  |
| 206 | AXIN1 | c.792dupC | p.G265fs*20 | loss | Frameshift | 3 | likely pathogenic | 0.19 |  |
| 206 | CDKN1B | c.275dupC | p.R93fs*32 | loss | Frameshift |  |  | 0.2 |  |
| 206 | NOTCH3 | c.5882delA | p.N1961fs*51 | loss | Frameshift | 3 | likely pathogenic | 0.2 |  |
| 206 | PIK3C2B | c.4096C>T | p.R1366* | loss | Stop Gain | 3 | likely pathogenic | 0.2 |  |
| 206 | PRSS8 | c.124delC | p.Q42fs*64 | loss | Frameshift | 3 | likely pathogenic | 0.26 |  |
| 207 | NRAS | c.181C>A | p.Q61K | gain | Missense mutation | 2C | pathogenic | 0.4 |  |
| 207 | PTEN | c.697C>T | p.R233* | loss | Stop gain | 2C | pathogenic | 0.31 |  |
| 207 | PTEN | c.493G>A | p.G165R | loss | Missense mutation | 2C | pathogenic | 0.43 |  |
| 207 | TP53 | c.154C>T | p.Q52Ter | loss | Stop gain | 2C | pathogenic | 0.74 |  |
| 207 | CCND1 | c.857C>G | p.T286R | gain | Missense mutation | 2C | likely pathogenic | 0.68 |  |
| 207 | RECQL4 | c1868G>A | p.R623H | loss | Missense mutation | 3 | likely pathogenic | 0.54 |  |
| 209 | KRAS | c.35G>A | p.G12D | gain | Missense mutation | 1A | pathogenic | 0.25 |  |
| 209 | TP53 | c.104del | p.L35fs*9 | loss | Frameshift | 2C | pathogenic | 0.32 |  |
| 209 | PRKN | c.823C>T | p.R275W | loss | Missense mutation | 3 | likely pathogenic | 0.48 |  |
| 209 | RB1 | c.2117_2118del | p.C706fs*14 | loss | Frameshift | 3 | likely pathogenic | 0.37 |  |
| 210 | BRCA2 | c.3847_3848delGT | p.V1283fs*2 | loss | Frameshift | 2C | pathogenic | 0.41 | SCC |
| 210 | LRP1B | c.13560+1G>A |  | loss | Splicing | 3 | likely pathogenic | 0.31 | SCC |
| 210 | NOTCH2 | c.7075C>G | p.P2359A | loss | Missense mutation | 3 | likely pathogenic | 0.47 | SCC |
| 210 | FANCL | c.343_364del | p.I115fs*17 | loss | Frameshift | 2C | likely pathogenic | 0.33 | Osteosarcoma |
| 210 | NOTCH2 | c.7075C>G | p.P2359A | loss | Missense mutation | 3 | likely pathogenic | 0.55 | Osteosarcoma |
| 210 | PIK3C2G | c.3307_3341del | p.>1103fs*13 | loss | Frameshift | 3 | likely pathogenic | 0.11 | Osteosarcoma |
| 211 | TET2 | c.4513G>A | p.A1505T | loss | Missense mutation | 2C | likely pathogenic | 0.4 |  |
| 213 | TP53 | c.453C>A | p.P151H |  | Missense mutation | 2C | pathogenic | 0.29 |  |
| 214 | KRAS | c.34G>T | p.G12C | gain | Missense mutation | 2C | pathogenic | 0.23 |  |
| 214 | TP53 | c.818G>A | p.R273H | gain | Missense mutation | 2C | pathogenic | 0.47 |  |
| 214 | ARID1A | c.1552dupT | p.Y518fs*105 | loss | Frameshift mutation | 2C | likely pathogenic | 0.19 |  |
| 214 | CDKN2A | c.281delT | p.94fs*52 | loss | Frameshift mutation | 2C | likely pathogenic | 0.37 |  |
| 214 | SMAD4 | c1495T>C | p.C499R | loss | Missense mutation | 3 | likely pathogenic | 0.29 |  |
| 215 | CDKN2A | c.45G>A | p.W15* | loss | Stop gain | 2C | pathogenic | 0.1 |  |
| 215 | MET | c.2962C>T | p.R988C | gain | Missense mutation | 2C | pathogenic | 0.43 |  |
| 215 | TP53 | c.636delT | p.R213fs*34 | loss | Frameshift mutation | 2C | pathogenic | 0.07 |  |
| 216 | ARID1A | c.671dupC | p.P225fs*175 | loss | Frameshift mutation | 2C | pathogenic | 0.55 |  |
| 216 | BAP1 | c.666delC | p.Y223fs*8 | loss | Frameshift mutation | 2C | likely pathogenic | 0.49 |  |
| 216 | FGFR2 | c.827T>G | p.F276C | gain | Missense mutation | 2C | likely pathogenic | 0.45 |  |
| 217 | ARID1A | c.6259G>A | p.G2087R | loss | Missense mutation | 2C | pathogenic | 0.17 |  |
| 217 | ARID2 | c.1924G>T | p.G642 | loss | Stop gain | 2C | likely pathogenic | 0.05 |  |
| 217 | FAT1 | c.9229+1G>A |  | loss | Splicing | 3 | likely pathogenic | 0.05 |  |
| 217 | GRM3 | c.2392-1G>T |  | loss | Splicing | 3 | likely pathogenic | 0.07 |  |
| 218 | PALB2 | c.509_510delGA | p.R170fs*14 | loss | Frameshift mutation | 1A | pathogenic | 0.69 |  |
| 218 | PIK3CA | c.1616C>G | p.539R | gain | Missense mutation | 2C | pathogenic | 0.17 |  |
| 218 | DICER1 | c.5138A>T | p.D1713V | loss | Missense mutation | 3 | likely pathogenic | 0.06 |  |
| 218 | RARA | c.888_904del | p.R297fs*46 | loss | Frameshift mutation | 3 | likely pathogenic | 0.16 |  |
| 218 | ERBB2 | c.2286A>T | p.KK762N | gain | Missense mutation | 3 | VUS | 0.06 |  |
| 219 | CDKN2A | c.143C>T | p.P48L | loss | Missense mutation | 1A | pathogenic | 0.41 |  |
| 219 | KRAS | c.35G>A | p.G12D | gain | Missense mutation | 2C | pathogenic | 0.22 |  |
| 219 | TP53 | c.394A>C | p.K132Q | loss | Missense mutation | 2C | pathogenic | 0.35 |  |
| 219 | PTEN | c.760_762dupAAA | p.K254dup | loss | In Frame Duplication | 2C | likely pathogenic | 0.43 |  |
| 220 | CHEK2 | c.1229delC | p.T410fs*15 | loss | Frameshift mutation | 2C | pathogenic | 0.43 |  |
| 220 | KRAS | c.35G>C | p.G12A | gain | Missense mutation | 2C | pathogenic | 0.05 |  |
| 222 | TP53 | c.559+1G>A |  | loss | Splicing | 2C | pathogenic | 0.26 |  |
| 223 | FBXW7 | c.2065C>T | p.Arg689Trp | loss | Missense mutation | 2C | pathogenic | 0.3 |  |
| 223 | KRAS | c.35G>T | p.Gly12Val | gain | Missense mutation | 2C | pathogenic | 0.19 |  |
| 223 | ARID1A | c.608dupA | p.His203GlnfsTer197 | loss | Frameshift mutation | 2C | likely pathogenic | 0.2 |  |
| 223 | NF1 | c.3623_3624delinsC | p.Leu1208SerfsTer7 | loss | Frameshift mutation |  | pathogenic | 0.2 |  |
| 223 | GNAS | c.602G>A | p.Arg201His | gain | Missense mutation | 3 | pathogenic | 0.14 |  |
| 223 | SMAD4 | c.1081C>T | p.Arg361Cyc | loss | Missense mutation | 3 | pathogenic | 0.19 |  |
| 225 | BRAF | c.1801A>G | p.K601E | gain | Missense mutation | 2C | pathogenic | 0.3 |  |
| 225 | STK11 | c.633_633del | p.T212fs*65 | loss | Frameshift mutation | 2C | likely pathogenic | 0.09 |  |
| 226 | TGFBR1 | c.452_454delAAG | p.E151del | loss | Frameshift mutation | 3 | likely pathogenic | 0.17 |  |
| 228 | ATM | c.8542A>T | p.K2848 | loss | Stop gain | 3 | likely pathogenic | 0.46 |  |
| 229 | PBRM1 | c.237-2A>G |  | loss | splicing |  |  | 0.32 |  |
| 232 | KRAS | c.35G>A | p.G12D | gain | Missense mutation | 2C | pathogenic | 0.09 |  |
| 234 | RAD51C | c.790G>A | p.G264S | loss | Missense mutation | 2C | pathogenic | 0.48 |  |
| 236 | TP53 | c.833C>T | p.P278L | loss | Missense mutation | 2C | pathogenic | 0.21 |  |
| 236 | NBN | c.1835C>G | p.P612R | loss | Missense mutation | 2C | likely pathogenic | 0.49 |  |
| 236 | FANCI | c.3541G>A | p.A1181T | loss | Splicing | 3 | pathogenic | 0.53 |  |
| 237 | ARID1A | c.1393C>T | p.Q465* | loss | Stop gain | 2C | pathogenic | 0.12 |  |
| 237 | KRAS | c.35G>T | p.G12V | gain | Missense mutation | 2C | pathogenic | 0.22 |  |
| 238 | BRAF | c.1801A>G | p.K601E | gain | Missense mutation | 2C | pathogenic | 0.16 |  |
| 238 | MAP2K1 | c.383G>A | p.G128D | gain | Missense mutation | 2C | pathogenic | 0.13 |  |
| 238 | TP53 | c.818G>A | p.R273H | gain | Missense mutation | 2C | pathogenic | 0.03 |  |
| 238 | ARID1A | c.6842dupT | p.L2281fs | loss | Frameshift mutation | 2C | likely pathogenic | 0.15 |  |
| 239 | BAP1 | c.1182C>A | p.Y394* | loss | Stop gain | 2C | likely pathogenic | 0.67 |  |
| 239 | FGFR2 | c.827T>G | p.F276C | gain | Missense mutation | 2D | likely pathogenic | 0.8 |  |
| 240 | ARID1A | c.4003C>T | p.R1335* | loss | Splicing | 2C | likely pathogenic | 0.14 |  |
| 241 | POLE | c.1277C>T | p.A426V | gain | Missense mutation | 2C | pathogenic | 0.43 |  |
| 241 | TP53 | c.431A>C | p.Q144P | loss | Missense mutation | 2C | likely pathogenic | 0.48 |  |
| 241 | CSF1R | c.1441C>T | p.Q481Ter | loss | Stop gain | 3 | pathogenic | 0.22 |  |
| 242 | ARID1A | c.6806C>A | p.S2269Ter | loss | Stop gain | 2C | likely pathogenic | 0.12 |  |
| 242 | CDKN2A | c.265_274del | p.G89fs*54 | loss | Frameshift mutation | 2C | likely pathogenic | 0.14 |  |
| 242 | ARID2 | c.229_230del | p.N77fs*7 | loss | Frameshift mutation | 3 | likely pathogenic | 0.21 |  |
| 243 | SMARCA4 | c.2917C>T | p.R973W | loss | Missense mutation | 2C | likely pathogenic | 0.06 |  |
| 244 | IDH1 | c.394C>T | p.R132C | gain | Missense mutation | 1A | pathogenic | 0.23 |  |
| 244 | NF1 | c.5839C>T | p.R1947* | loss | Stop gain | 2C | pathogenic | 0.23 |  |
| 244 | PTCH1 | c.3606delC | p.S1203fs*52 | loss | Frameshift mutation | 2C | pathogenic | 0.13 |  |
| 244 | TP53 | c.216delC | p.V73fs*50 | loss | Frameshift mutation | 2C | pathogenic | 0.17 |  |
| 244 | TP53 | c.916C>T | p.R306* | loss | Stop gain | 2C | pathogenic | 0.22 |  |
| 244 | ARID1A | c.4899dupC | p.M1634fs*14 | loss | Frameshift mutation | 2C | likely pathogenic | 0.3 |  |
| 244 | PIK3R1 | c.1669C>T | p.R557* | loss | Stop gain | 2C | likely pathogenic | 0.28 |  |
| 244 | PDCD1 | c.105delC | p.T36fs*9 | loss | Frameshift mutation | 3 | likely pathogenic | 0.39 |  |
| 245 | TP53 | c.493C>T | p.Q165* | loss | Stop gain | 2C | pathogenic | 0.12 |  |
| 245 | PTEN | c.47_48delAT | p.Y16fs*27 | loss | Frameshift mutation | 2C | likely pathogenic | 0.12 |  |
| 246 | KRAS | c.35G>C | p.G12A | gain | Missense mutation | 2C | pathogenic | 0.3 |  |
| 247 | CDKN2A | c.238C>T | p.R80* | loss | Stop gain | 2C | pathogenic | 0.39 |  |
| 248 | TP53 | c.646G>A | p.V216M | loss | Missense mutation | 2C | pathogenic | 0.71 |  |
| 248 | MET | c.504G>T | p.E168D | gain | Missense mutation | 3 | VUS | 0.43 |  |
| 249 | ARID1A | c.6669dupT | p.E2224* | loss | Frameshift mutation | 2C | likely pathogenic | 0.36 |  |
| 250 | KRAS | c.35G>A | p.G12D | gain | Missense mutation | 1A | pathogenic | 0.47 |  |
| 250 | CHEK2 | c.599T>C | p.I200T | loss | Missense mutation | 2C | pathogenic | 0.35 |  |
| 251 | IDH1 | c.394C>T | p.R132C | gain | Missense mutation | 1A | pathogenic | 0.22 |  |
| 251 | BAP1 | c.1153C>T | p.R385* | loss | Stop gain | 2C | pathogenic | 0.58 |  |
| 251 | NRAS | c.34G>A | p.G12S | gain | Missense mutation | 2C | pathogenic | 0.21 |  |
| 251 | PIK3CA | c.1133G>T | p.C378F | gain | Missense mutation | 2C | pathogenic | 0.24 |  |
| 251 | TP53 | c.824G>T | p.C275F | loss | Missense mutation | 2C | pathogenic | 0.14 |  |
| 251 | PIK3CA | c.3140A>G | p.H1047R | gain | Missense mutation | 2D | pathogenic | 0.25 |  |
| 251 | PBRM1 | c.3534-1G>T |  | loss | Splicing | 3 | likely pathogenic | 0.09 |  |

Clinical significance according to the AMP / ASCO /CAP guidelines[217].

**Table S5:** Detected copy number variations ≥ 2 fold-change

| **PatID** | **Gene** | **Fold-change** |
| --- | --- | --- |
| 6 | ESR1 | 9.202 |
| 6 | FGF23 | 3.523 |
| 6 | FGF6 | 4.15 |
| 6 | CDK4 | 27.316 |
| 6 | MDM2 | 37.485 |
| 7 | FGFR3 | 2.01 |
| 8 | FGFR1 | 2.763 |
| 10 | CDK4 | 2.377 |
| 11 | FGF14 | 2.141 |
| 11 | FGF7 | 3.027 |
| 14 | PDGFRA | 3.157 |
| 14 | KIT | 2.987 |
| 21 | MET | 12.266 |
| 34 | FGF23 | 2.2 |
| 34 | FGF6 | 2 |
| 36 | FGF4 | 2.37 |
| 36 | FGF3 | 2.055 |
| 39 | FGFR3 | 2.216 |
| 39 | RICTOR | 2.011 |
| 39 | FGF10 | 2.061 |
| 39 | FGFR2 | 2.132 |
| 40 | FGF10 | 2.094 |
| 45 | CDK4 | 4.347 |
| 45 | MDM2 | 5.448 |
| 46 | ERBB2 | 19.581 |
| 47 | ERBB2 | 2.342 |
| 47 | CCNE1 | 6.214 |
| 49 | RICTOR | 2.114 |
| 53 | CCND1 | 2.74 |
| 53 | FGF19 | 3.096 |
| 53 | FGF4 | 3.002 |
| 53 | FGF3 | 3.145 |
| 55 | FGFR3 | 2.476 |
| 55 | FGFR4 | 2.587 |
| 55 | MYC | 2.279 |
| 56 | BRCA2 | 2.023 |
| 57 | CCNE1 | 3.072 |
| 70 | CCNE1 | 2.267 |
| 71 | CCND1 | 3.148 |
| 71 | FGF19 | 2.674 |
| 71 | FGF4 | 2.281 |
| 71 | FGF3 | 2.394 |
| 73 | EGFR | 13.384 |
| 77 | MDM4 | 3.899 |
| 85 | FGFR2 | 5.56 |
| 90 | CCND1 | 4.338 |
| 94 | TFRC | 2.209 |
| 94 | ERBB2 | 11.896 |
| 95 | CCND1 | 3.846 |
| 95 | FGF19 | 4.584 |
| 95 | FGF4 | 4.309 |
| 95 | FGF3 | 4.664 |
| 97 | CCND3 | 2.176 |
| 99 | LAMP1 | 2.427 |
| 99 | FGF14 | 2.448 |
| 100 | RICTOR | 3.084 |
| 100 | FGF10 | 2.143 |
| 100 | CCND3 | 2.713 |
| 100 | FGF7 | 2.87 |
| 119 | MYC | 11.649 |
| 119 | FGF3 | 2.023 |
| 119 | KRAS | 11.499 |
| 123 | CCND1 | 2.667 |
| 126 | PIK3CA | 3.35 |
| 130 | AKT2 | 3.207 |
| 131 | CCND3 | 3.334 |
| 133 | MET | 2.452 |
| 136 | MDM2 | 2.983 |
| 138 | MYC | 4.112 |
| 139 | RICTOR | 2.078 |
| 139 | MET | 2.5 |
| 139 | BRAF | 2.567 |
| 139 | MYC | 2.624 |
| 139 | FGF23 | 2.527 |
| 139 | FGF6 | 2.335 |
| 139 | CCNE1 | 2.399 |
| 146 | MDM2 | 5.253 |
| 147 | MYC | 2.204 |
| 149 | CCND1 | 11.038 |
| 149 | ERBB3 | 4.989 |
| 149 | CDK4 | 3.91 |
| 152 | MET | 2.018 |
| 152 | CDK6 | 2.044 |
| 152 | FGF3 | 6.115 |
| 152 | FGF4 | 6.876 |
| 152 | CCND1 | 7.027 |
| 152 | FGF19 | 7.545 |
| 152 | MYCL | 9.522 |
| 154 | MYC | 3.29 |
| 155 | MYCl | 11.767 |
| 162 | EGFR | 9.763 |
| 162 | MYC | 7.07 |
| 162 | CCND1 | 11.156 |
| 162 | FGF19 | 6.58 |
| 162 | FGF4 | 6.127 |
| 162 | FGF3 | 7.717 |
| 174 | LAMP1 | 2.107 |
| 175 | CCND1 | 2.092 |
| 175 | MYC | 2.277 |
| 175 | MYCN | 2.553 |
| 182 | ERBB2 | 44.536 |
| 183 | CDK4 | 2.398 |
| 183 | CCND1 | 2.676 |
| 183 | MDM2 | 6.939 |
| 191 | FGFR2 | 46.137 |
| 191 | MYC | 3.241 |
| 193 | PIK3CA | 2.289 |
| 198 | MET | 7.406 |
| 198 | ERBB2 | 9.467 |
| 199 | FGF9 | 2.089 |
| 199 | MYC | 2.945 |
| 201 | ERBB2 | 57.278 |
| 203 | FGFR2 | 2.038 |
| 203 | MYCL | 2.171 |
| 203 | FGFR1 | 3.624 |
| 203 | MDM4 | 4.12 |
| 205 | MDM4 | 2.203 |
| 207 | PIK3CB | 2.042 |
| 207 | PIK3CA | 2.096 |
| 207 | MYCN | 2.484 |
| 207 | FGF10 | 2.769 |
| 207 | RICTOR | 2.876 |
| 208 | ALK | 2.037 |
| 209 | ERCC1 | 2.628 |
| 210 | MYC | 2.462 |
| 211 | KRAS | 28.606 |
| 219 | FGFR1 | 2.025 |
| 227 | MYC | 2.036 |
| 228 | RAF1 | 2.148 |
| 248 | CDK6 | 2.511 |
| 249 | EGFR | 13.582 |

**Table S6:** Detected genetic rearrangements

| **PatID** | **Gene 1** | **Gene 2** | **Gene 1 exon** | **Gene 2 exon** |
| --- | --- | --- | --- | --- |
| 3 | NAB2 | STAT6 | exon 6 | exon 16 |
| 6 | CDK4 | CNOT2 |  |  |
| 9 | KMT2A | PEX12 |  |  |
| 12 | JAK2 | INSL4 |  |  |
| 12 | U2AF1 | ERG |  |  |
| 14 | SEC16A | NOTCH1 | exon 11 | exon 11 |
| 14 | CLTC | RPS6KB1 | exon 17 | exon 2 |
| 20 | FGFR2 | ATE1 |  |  |
| 22 | FGFR2 | BICC1 |  |  |
| 27 | FGFR2 | PHGDH | exon 17 | exon 9 |
| 30 | FGFR2 | MYH16 |  |  |
| 31 | STRN | ALK | exon 3 | exon 20 |
| 39 | DHX57 | ALK | exon 1 | exon 2 |
| 47 | NOTCH1 | LCN10 |  |  |
| 49 | TANC2 | RPS6KB1 |  |  |
| 56 | ERBB2 | RAB11FIP4 | exon 14 | intron 4 |
| 75 | EEF1B2 | MET | exon 4 | exon 15 |
| 75 | MET | GRASP | exon 14 | intron 1 |
| 75 | POLA1 | MET | intron 26 | exon 15 |
| 80 | GPRC5C | MET |  |  |
| 82 | MXD4 | NUTM1 |  |  |
| 85 | FGFR2 | ABLIM1 |  |  |
| 99 | SNAPC4 | NOTCH1 | exon 21 | exon 26 |
| 117 | TMPRSS2 | ERG |  |  |
| 119 | LACE1 | ROS1 |  |  |
| 123 | EWSR1 | FLI1 |  |  |
| 124 | KMT2A | DYNC2H1 |  |  |
| 127 | ERBB2 | STARD3 |  |  |
| 133 | FAM133B | EGFR |  |  |
| 155 | RAF1 | NKTR | exon 1 | exon 9 |
| 155 | ELP4 | RAF1 | intron 9 | exon 1 |
| 157 | RPS6KB1 | VMP1 | exon 1 | exon 11 |
| 166 | BRAF | AGK | exon 7 | exon 3 |
| 166 | AGK | BRAF | exon 2 | exon 8 |
| 191 | RPS6KB1 | VMP1 | exon 1 | exon 8 |
| 201 | CAPZA2 | MET | exon 4 | exon 3 |
| 202 | TCOF1 | PDGFRB | exon 3 | exon 12 |
| 205 | ERSR1 | IRF6 | exon 4 | exon 9 |
| 212 | FGFR2 | NDC80 | exon 17 | exon 13 |
| 233 | FGFR2 | NOL4 |  |  |

**Table S7:** Overview of patients receiving alternative therapies

| **Pat ID** | **Cancer Type** | **Alternative Therapy** | **L (time of MTB/ EMA)** | **R** | **Preceding therapy lines (excluding adjuvant/ neoadjuvant therapies)** |
| --- | --- | --- | --- | --- | --- |
| 79 | Gastric cancer | FOLFIRI | on | CR | 1 |
| 62 | Urothelial carcinoma | INN-Sacituzumab | off | PR | 3 |
| 65 | eCCA | Gemcitabine + Cisplatin | on | PR | 2 |
| 68 | Colorectal Cancer | Capecitabine + bevacizumab | on | PR | 2 |
| 69 | Adrenal Carcinoma | Nivolumab | off | PR | 5 |
| 73 | Squamous esophageal carcinoma | Nivolumab | on | PR | 1 |
| 75 | iCCA | NAPOLI | off | PR | 1 |
| 78 | CUP | Doxorubicin + cisplatin | off | PR | 1 |
| 81 | iCCA | Pembrolizumab + lenvatinib | off | PR | 3 |
| 82 | Undifferentiated round cell sarcoma | Trabectedin | on | PR | 1 |
| 88 | Adenoid cystic carcinoma | Lenvatinib | off | PR | 1 |
| 90 | Urothelial carcinoma | Cabozantinib | off | PR | 2 |
| 92 | Pancreatic cancer | NAPOLI | on | PR | 4 |
| 97 | Gastric cancer | Paclitaxel + ramucirumab | on | PR | 1 |
| 99 | T cell acute lymphoblastic lymphoma | Cyclophosphamide + methotrexate + nelarabin | on | PR | 2 |
| 59 | CUP | Gemcitabine | on | MR | 2 |
| 66 | Leiomyosarcoma | Trabectedin | on | MR | 3 |
| 96 | Mixed HCC/CCA | Lenvatinib | on | MR | 1 |
| 48 | CRC | Lonsurf | on | SD | 1 |
| 49 | Breast cancer | Capecitabine | on | SD | 4 |
| 54 | Pancreatic cancer | NAPOLI | on | SD | 3 |
| 67 | Breast cancer | Eribulin | on | SD | 4 |
| 71 | HNSCC | Methotrexat | on | SD | 3 |
| 74 | Appendiceal carcinoma | FOLFIRI + ramucirumab | on | SD | 6 |
| 77 | Breast cancer | Exemestan | on | SD | 9 |
| 84 | eCCA | Lapatinib + trametinib | off | SD | 2 |
| 86 | Colorectal Cancer | Lonsurf | on | SD | 4 |
| 94 | eCCA | NAPOLI | off | SD | 2 |
| 95 | Undifferentiated esophageal carcinoma | Nivolumab | off | SD | 2 |
| 98 | Urothelial carcinoma | FOLFIRI | off | SD | 1 |
| 50 | Ovarian cancer | Gemcitabine + Treosulfan | off | PD | 3 |
| 51 | Prostate cancer | Lutetium-177-PSMA | off | PD | 4 |
| 52 | Prostate cancer | Lutetium-177-PSMA | off | PD | 4 |
| 53 | HNSCC | Methotrexat | on | PD | 1 |
| 55 | HCC | Cabozantinib | on | PD | 3 |
| 57 | Ovarian cancer | Tamoxifen | off | PD | 6 |
| 58 | Uterine leiomyosarcoma | Pazopanib | on | PD | 1 |
| 60 | Neuroendocrine tumour | Cabozantinib | off | PD | 5 |
| 61 | Malignant granular cell tumour | Pazopanib | on | PD | 2 |
| 64 | CUP | Megestrolacetat | off | PD | 4 |
| 70 | iCCA | Pembrolizumab + lenvatinib | off | PD | 1 |
| 72 | iCCA | FOLFOX6 | on | PD | 2 |
| 83 | Colorectal Cancer | Lonsurf | on | PD | 2 |
| 85 | CUP | Carboplatin-taxol | on | PD | 1 |
| 91 | Colorectal Cancer | FOLFOXIRI + bevacizumab | on | PD | 3 |
| 93 | HNSCC | Nivolumab | on | PD | 4 |
| 100 | Pancreatic cancer | FOLFIRINOX | on | PD | 1 |
| 56 | Colorectal Cancer | Lonsurf + bevacizumab | on | NA | 4 |
| 63 | Adenoid cystic carcinoma | Cisplatin + Vinorelbin | off | NA | NA |
| 76 | Osteosarcoma | Ipilimumab + nivolumab | off | NA | 2 |
| 80 | Leiomyosarcoma | Cisplatin + pemetrexed + pembrolizumab | on | NA | 1 |
| 87 | iCCA | Docetaxel + gemcitabine | off | NA | 1 |
| 89 | Gastric cancer | FLOT | on | NA | 1 |

R, response; L, label; NA, not applicable; EMA: European Medicines Agency; iCCA, intrahepatic cholangiocarcinoma; dCCA, distal cholangiocarcinoma; CUP, carcinoma of unknown primary; HNSCC, head and neck squamous cell carcinoma; mixed HCC/CCA, mixed cholangiocellular-hepatocellular carcinoma; SD, stable disease; PD, progressive disease; CR, complete remission; PR, partial remission; MR, mixed response.

**Table S8:** PFS2/1 ratios in patients receiving MTB therapies

| **Pat ID** | **Cancer Type** | **Board Recommendation** | **R to MTB therapy** | **PFS MTB therapy (PFS2)** | **Previous therapy** | **PFS previous therapy (PFS1)** | **Ratio PFS2/1** | **PFS2/1 > 1.3** |
| --- | --- | --- | --- | --- | --- | --- | --- | --- |
| 22 | iCCA | Pemigatinib | PR | 260 | FOLFIRI | 50 | 5.20 | Yes |
| 29 | Gastric adenocarcinoma,  diffuse type | Pembrolizumab | PR | 572 | FLOT | 203 | 2.82 | Yes |
| 42 | Mucosal melanoma | Imatinib | MR | 91 | Nivolumab + ipilimumab | 33 | 2.76 | Yes |
| 36 | GIST | Regorafenib | SD | 189 | Sunitinib | 76 | 2.49 | Yes |
| 25 | Peripheral T-cell lymphoma | Azacitidine + ascorbate | NA | 70 | Gemcitabine | 31 | 2.26 | Yes |
| 40 | Type B3 thymoma | Pembrolizumab | SD | 290 | VIP-E | 164 | 1.77 | Yes |
| 33 | pCCA | Pembrolizumab + lenvatinib | SD | 297 | Gemcitabin + Cisplatin | 182 | 1.63 | Yes |
| 30 | iCCA | Pemigatinib | PD | 57 | FOLFIRI + zolendronate | 35 | 1.63 | Yes |
| 6 | Dedifferentiated liposarcoma | Palbociclib | PD | 100 | Eribulin | 74 | 1.35 | Yes |
| 1 | Lung adenocarcinoma | Afatinib + paclitaxel | SD | 232 | Durvalumab | 216 | 1.07 | No |
| 43 | Adenocarcinoma of the esophagogastric junction | Trastuzumab-Deruxtecan | PR | 75 | CF-Trastuzumab | 76 | 0.99 | No |
| 28 | CUP (adenocarcinoma, pelvic and hepatic) | Binimetinib + trametinib + cetuximab | PR | 369 | Bevacizumab + FOLFOX | 426 | 0.87 | No |
| 35 | Salivary adenocarcinoma, NOS | Bicalutamide + trenantone | PD | 192 | Trastuzumab Emtansin | 222 | 0.86 | No |
| 9 | Uterine leiomyosarcoma | Pembrolizumab | SD | 93 | Carboplatin + paclitaxel | 122 | 0.76 | No |
| 15 | Nonseminomatous Germ cell tumour | Pembrolizumab | PD | 55 | Gemcitabin + oxaliplatin + paclitaxel | 83 | 0.66 | No |
| 14 | Salivary adenocarcinoma, NOS | Pembrolizumab | PD | 41 | Bicalutamid + trenantone | 79 | 0.52 | No |
| 3 | SFT | Pazopanib | NA | 98 | DTIC | 206 | 0.48 | No |
| 12 | Dedifferentiated chondrosarcoma | Pembrolizumab | PD | 67 | Doxorubicin + ifosfamid | 152 | 0.44 | No |
| 18 | Gall bladder adenocarcinoma | Pembrolizumab + lenvatinib | PD | 63 | Gemcitabin + cisplatin | 144 | 0.44 | No |
| 10 | Salivary duct  carcinoma | Bicalutamide + trenantone | SD | 105 | Cisplatin | 280 | 0.38 | No |
| 17 | iCCA | Pembrolizumab | PD | 49 | FOLFIRI | 139 | 0.35 | No |
| 32 | Breast neuroendocrine carcinoma | Palbociclib + Fulvestrant | PD | 75 | Capecitabin + temozolomide | 220 | 0.34 | No |
| 16 | iCCA | Olaparib | SD | 132 | Gemcitabin + cisplatin | 552 | 0.24 | No |
| 4 | Papillay thyroid carcinoma | Pembrolizumab | PD | 59 | Lenvatinib | 337 | 0.18 | No |
| 5 | T-PLL | Ruxolitinib | PR | 46 | Alemtuzumab | 264 | 0.17 | No |
| 19 | Lung adenocarcinoma | Mobocertinib | PR | 126 | Vinorelbin | 1399 | 0.09 | No |

R, response; NA, not applicable; iCCA, intrahepatic cholangiocarcinoma; pCCA, perihilar cholangiocarcinoma; dCCA, distal cholangiocarcinoma; SFT, solitary fibrous tumour; CUP, carcinoma of unknown primary; SD, stable disease; PD, progressive disease; PR, partial remission; MR, mixed response; PFS, progression free survival.

**Table S9:** PFS2/1 ratios in patients receiving alternative therapies

| **Pat ID** | **Cancer type** | **Alternative therapy** | **R to alternative therapy** | **PFS alternative therapy (PFS1)** | **Previous therapy** | **PFS previous therapy (PFS0)** | **Ratio PFS2/1** | **PFS2/1 > 1.3** |
| --- | --- | --- | --- | --- | --- | --- | --- | --- |
| 93 | HNSCC | Nivolumab | PD | 323 | Methotrexate | 85 | 3.80 | yes |
| 52 | Prostate cancer | Lutetium-177-PSMA | PD | 303 | Enzalutamide | 109 | 2.78 | yes |
| 61 | Malignant granular cell tumour | Pazopanib | PD | 118 | Trabectedin | 67 | 1.76 | yes |
| 70 | iCCA | Pembrolizumab + lenvatinib | PD | 92 | Gemcitabine + cisplatin | 59 | 1.56 | yes |
| 49 | Breast cancer | Capecitabine | SD | 210 | Docetaxel | 153 | 1.37 | yes |
| 50 | Ovarian cancer | Gemcitabine + Treosulfan | PD | 111 | Topotecan | 91 | 1.22 | no |
| 51 | Prostate cancer | Lutetium-177-PSMA | PD | 128 | Enzalutamide | 105 | 1.22 | no |
| 78 | CUP | Doxorubicin + cisplatin | PR | 395 | Bevacizumab + FOLFOX | 360 | 1.10 | no |
| 91 | Colorectal cancer | FOLFOXIRI + bevacizumab | PD | 180 | Cetuximab + encorafenib | 165 | 1.09 | no |
| 55 | HCC | Cabozantinib | PD | 98 | Lenvatinib | 91 | 1.08 | no |
| 60 | Neuroendocrine tumour | Cabozantinib | PD | 85 | FOLFOX | 79 | 1.08 | no |
| 58 | Uterine leiomyosarcoma | Pazopanib | PD | 61 | Trabectedin | 61 | 1.00 | no |
| 100 | Pancreatic cancer | FOLFIRINOX | PD | 133 | FOLFIRINOX | 133 | 1.00 | no |
| 79 | Gastric cancer | FOLFIRI | CR | 305 | FLOT | 335 | 0.91 | no |
| 59 | CUP | Gemcitabine | MR | 74 | Pembrolizumab | 82 | 0.90 | no |
| 72 | iCCA | FOLFOX6 | PD | 81 | Pembrolizumab + lenvatinib | 91 | 0.89 | no |
| 66 | Leiomyosarcoma | Trabectedin | MR | 33 | Gemcitabine + docetaxel | 63 | 0.52 | no |
| 65 | eCCA | Gemcitabine + cisplatin | PR | 163 | FOLFIRI | 318 | 0.51 | no |
| 84 | eCCA | Lapatinib + trametinib | SD | 61 | Gemcitabine + oxaliplatin | 146 | 0.42 | no |
| 48 | Colorectal cancer | Lonsurf | SD | 37 | FOLFIRI | 114 | 0.32 | no |
| 67 | Breast cancer | Eribulin | SD | 47 | Paclitaxel | 146 | 0.32 | no |
| 95 | Undifferentiated esophageal carcinoma | Nivolumab | SD | 56 | 5FU + cisplatin | 176 | 0.32 | no |
| 82 | Undifferentiated round cell sarcoma | Trabectedin | PR | 36 | Doxorubicin | 122 | 0.30 | no |
| 54 | Pancreatic cancer | NAPOLI | SD | 77 | Gemcitabine + nab-paclitaxel | 268 | 0.29 | no |
| 97 | Gastric cancer | Paclitaxel + ramucirumab | PR | 252 | Cisplatin + 5-FU + trastuzumab | 1051 | 0.24 | no |
| 92 | Pancreatic cancer | NAPOLI | PR | 34 | Gemcitabine + nab-paclitaxel | 169 | 0.20 | no |
| 71 | HNSCC | Methotrexat | SD | 35 | Nivolumab | 190 | 0.18 | no |
| 81 | iCCA | Pembrolizumab + lenvatinib | PR | 42 | FOLFIRI | 232 | 0.18 | no |
| 73 | Squamous esophageal carcinoma | Nivolumab | PR | 41 | Cisplatin + 5-FU | 236 | 0.17 | no |
| 94 | eCCA | NAPOLI | SD | 56 | Gemcitabine + Cisplatin | 406 | 0.14 | no |
| 75 | iCCA | NAPOLI | PR | 42 | Gemcitabine + Cisplatin | 476 | 0.09 | no |

R, response; L, label; NA, not applicable; iCCA, intrahepatic cholangiocarcinoma; dCCA, distal cholangiocarcinoma; CUP, carcinoma of unknown primary; HNSCC, head and neck squamous cell carcinoma; SD, stable disease; PD, progressive disease; CR, complete remission; PR, partial remission; MR, mixed response; PFS, progression free survival.
