# Supplementary figures and images for: Critical evaluation of molecular tumour board outcomes following 2 years of clinical practice in a Comprehensive Cancer Centre
Source: Br J Cancer. 2022 Dec 26;128(6):1134–47. doi: 10.1038/s41416-022-02120-x (PMC10006213; doi:10.1038/s41416-022-02120-x)

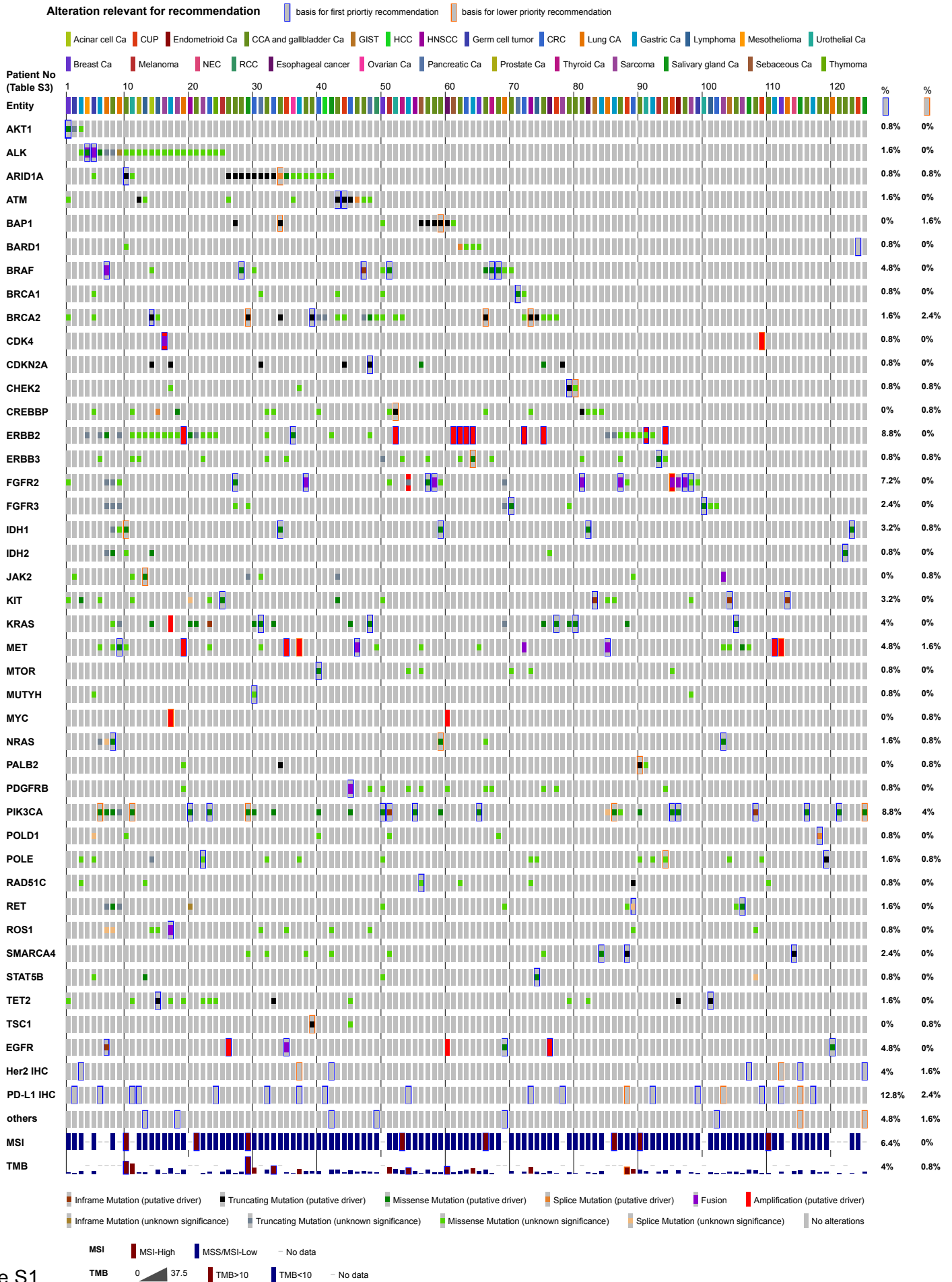

Figure S1

Supplement: Supplementary file 4 — Figure S1 [file 41416_2022_2120_MOESM4_ESM.pdf]

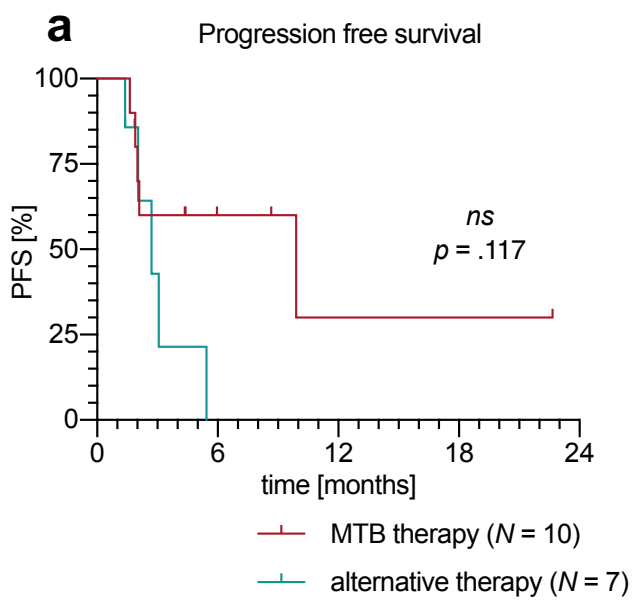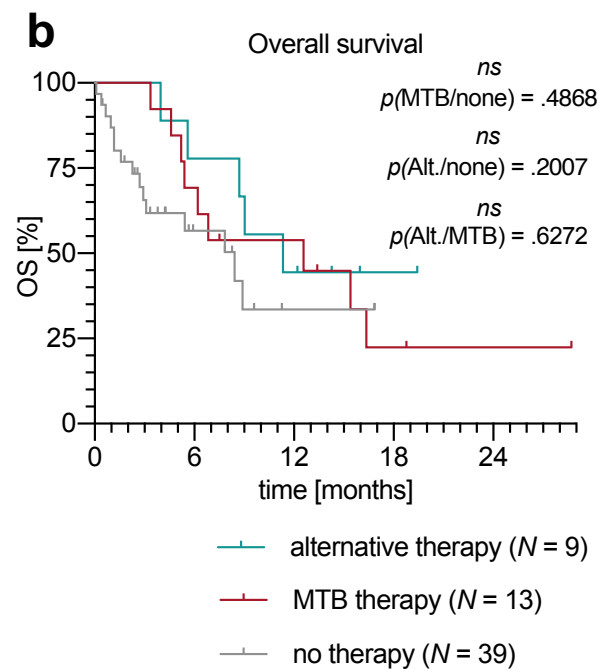

Figure S2

Supplement: Supplementary file 5 — Figure S2 [file 41416_2022_2120_MOESM5_ESM.pdf]
